# Investigation of encapsulated water wire within self-assembled hydrophilic nanochannels, in a modified γ^4^-amino acid crystals: Tracking thermally induced changes of intermolecular interactions within a crystalline hydrate

**DOI:** 10.1007/s00726-023-03372-4

**Published:** 2024-02-05

**Authors:** Krishnayan Basuroy, Jose de Jesus Velazquez-Garcia, Simone Techert

**Affiliations:** 1 Deutsches Elektronen-Synchrotron DESY, Notkestraße 85, 22607 Hamburg, Germany; 2 Institut für Röntgenphysik, Georg-August-Universität Göttingen, Friedrich-Hund-Platz 1, 37077 Göttingen, Germany

**Keywords:** Water wire, Nanochannel, Modified γ^4^-amino acids, Single-crystal X-ray diffraction, Water zipper, Self-assembly, Hydrogen bond, NBO calculation

## Abstract

Nanostructures formed by the self-assembly of modified/unmodified amino acids have the potential to be useful in several biological/nonbiological applications. In that regard, the greater conformational space provided by γ-amino acids, owing to their additional backbone torsional degrees of freedom and enhanced proteolytic stability, compared to their α-counterparts, should be explored. Though, modified single amino acid-based nanomaterials such as nanobelts or hydrogels are developed by utilizing the monosubstituted γ-amino acids derived from the backbone homologation of phenylalanine (Phe). Examples of a single γ-amino acid-based porous nanostructure capable of accommodating solvent molecules are not really known. The crystal structures of a modified γ^4^(*R*)Phe residue, Boc-γ^4^(*R*)Phe-OH, at different temperatures, showed that hydrogen-bonded water molecules are forming a wire inside hydrophilic nanochannels. The dynamics of intermolecular interactions between the water wire and the inner wall of the channel with relation to the temperature change was investigated by analyzing the natural bonding orbital (NBO) calculation results performed with the single crystal structures obtained at different temperature points. The NBO results showed that from 325 K onward, the strength of water–water interactions in the water wire are getting weaker, whereas, for the water–inner wall interactions, it getting stronger, suggesting a favorable change in the orientation of water molecules with temperatures, for the latter.

**Supplementary Information:**

The online version contains supplementary material available at 10.1007/s00726-023-03372-4.

## Introduction

Amino acids, the smallest building blocks for peptides and proteins, quite like the latter sequences, are also capable of forming several thermodynamically stable and ordered nanostructures through the process of self-assembly via non-covalent interactions such as hydrogen bonding, van der Waals interactions, π–π stacking, and electrostatic interactions (Chakraborty and Gazit 2018). Although various examples of self-assembled peptide-based nanomaterials are available (Ulijn and Smith 2008; Habibi et al. 2016), owing to their simpler structure, amino acids are less complicated compared to peptides or proteins, in terms of reliability, and accurately predicting the conformational possibilities (Chakraborty and Gazit 2018). Apart from being intrinsically biocompatible, amino acids are also water soluble, easier to synthesize, and are considered cheaper compared to peptides. Due to these reasons, over time, a “bottom-up” approach has been employed to design several hierarchical, complex nanomaterials, such as hydrogels of nanoscale order (Irwansyah et al. 2015), nanowires/nanorods (Koley and Pramanik 2014), nanotubes (Babar and Sarkar 2017; Shirvan et al. 2020), and nanofibrils (Adler-Abramovich et al. 2012) by the self-assembly of amino acids, that are tunable at the molecular level. The creation of various nanostructures is facilitated by the higher-order supramolecular secondary structures, such as helices, sheets, and turns, formed by the self-assembling amino acids through different non-covalent interactions (Chakraborty and Gazit 2018; Ren et al. 2021). The external stimuli such as pH, the polarity of the solvent, the reducing or oxidizing environment of the solution, and ionic strength, influence the self-assembly process of amino acids greatly (Shirvan et al. 2020; Bauri et al. 2018). This also allows the design of stimuli-responsive, highly tunable, single amino acid-based smart nanomaterials (Bauri et al. 2018).

Interestingly, most of the unmodified, single amino acid-based nanostructures, are developed with aromatic amino acids, with the exception of glycine-based fibrous nanostructures that are related to several genetic disorders (Chakraborty and Gazit 2018; Banik et al. 2016). In the beginning, the self-assembly of amyloid β (Aβ) was studied from a pathological perspective rather than an attempt to develop functional nanomaterials, due to the closeness of the phenomena with Alzheimer’s disease (Lesné et al. 2006). The self-assembly of phenylalanine (Phe) amino acids was also found to be responsible for phenylketonuria (PKU), an inherited, congenital metabolic disorder (Adler-Abramovich et al. 2012). Phenylalanine and its derivatives, due to the pH-sensitivity of their self-assembly process, have been also utilized in developing pH-responsive nanomaterials that show reversible changes in solubility and conformations depending on the pH level of the solution (Shirvan et al. 2020; Bauri et al. 2018). The intrinsic ability of Phe amino acid-based molecules to self-assemble into amyloid-like structures has been elaborately studied, from the perspective of thermodynamic and kinetic driving factors (Zaguri et al. 2021). Even the backbone homologated, monosubstituted γ-analogues of Phe amino acid, γ^3^Phe and γ^4^Phe residues are utilized in engineering nanobelts and hydrogels, respectively, and can be categorized as single amino acid-based nanostructures (Jeon et al. 2019; Misra et al. 2022). Except for these reports, there is almost no example of supramolecular nanostructures formed by the self-assembly of modified/unmodified, backbone homologated, monosubstituted γ-amino acids that have been closely investigated for their self-assembly patterns in crystals.

**Fig. 1:**
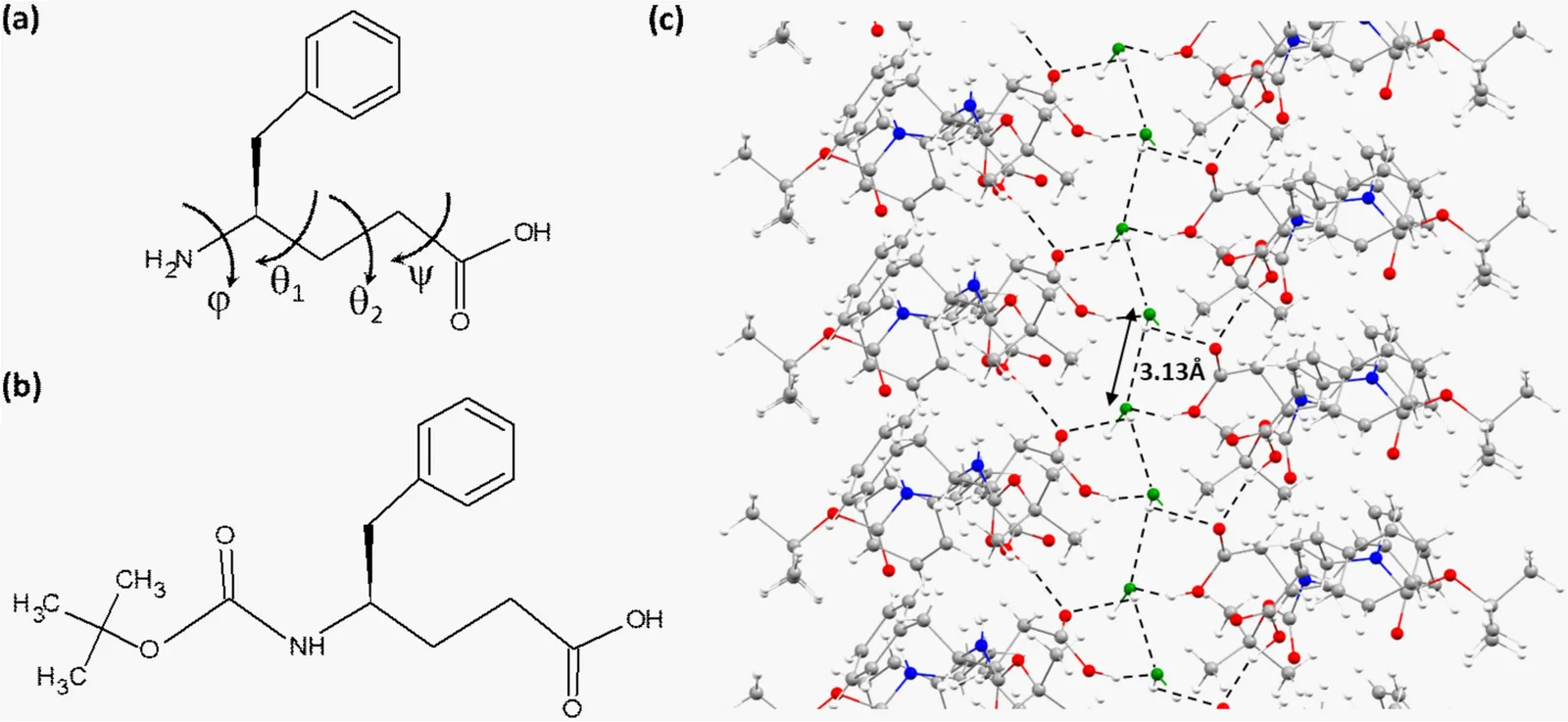
**a** Chemical structure of γ^4^Phe residue with the backbone torsion angles defined. **b** Chemical structure of Boc-γ^4^(*R*)Phe-OH. **c** Packing of molecules in Boc-γ^4^(*R*)Phe-OH crystals at 80 K showing the chain of water molecules in a hydrophilic channel, forming a zipper-like structure

In recent times, γ-amino acids derived from genetically coded α-amino acid residues by backbone homologation have garnered substantial interest owing to their superior proteolytic stability (Frackenpohl et al. 2001) and access to a larger conformational space compared to their α-counterparts (Vasudev et al. 2011; Basuroy et al. 2012, 2013a, b). The access to a diverse conformational space in terms of backbone torsion angles is introduced by the incorporation of two additional backbone atoms, C^β^ and C^γ^, that resulted in two additional torsional degrees of freedom, θ_1_ and θ_2_ (Fig. 1a). The monosubstituted γ-residues also proved to promote the adaption of helical conformation, both in homo-oligomers, (γγ)_n_, and in (αγ)_n_ hybrid hetero-oligomers (Basuroy et al. 2012, 2013a, b). The greater conformational diversity in the γ-residues is considered important for its utilization in mimicking secondary structures that occurs in proteins and also to explore unique secondary structures/ conformational features that are otherwise not precedent in nature (Vasudev et al. 2011). Apart from the earlier mentioned nanobelts and hydrogels developed from the modified/unmodified γ^4^ and γ^3^ amino acids, so far, the γ^4^ residues derived by the backbone homologation of α-amino acids have been successfully utilized by Gopi et al*.* in peptide oligomers to design thermoresponsive nanofibers, hydrogels, organogels, and nanotubes where in most of the cases the crystal structures of the peptides obtained from X-ray diffraction were also available to do a detailed analysis of the molecular conformation and self-assembly pattern (Jadhav and Gopi 2013; Jadhav et al. 2014; Misra et al. 2016, 2017).

For the present study, we have investigated a modified-protected γ^4^(*R*)Phe residue derivative, Boc-γ^4^(*R*)Phe-OH (BGPHEOH), where the N-terminus is protected by tert-butyloxycarbonyl (Boc) due to its assembling selectivity (Xu et al. 2009) (Fig. 1b). The idea was to explore the conformational features of a lone amino acid derivative and the nature of self-assembly in close aggregation, preferably, in a crystalline state when the backbone torsional degrees of freedom are not under a strong influence of intramolecular interactions, and intermolecular interactions such as intermolecular hydrogen bonds and van der Waals interactions/crystal contact forces are crucial. It was expected that the self-assembly process of the modified amino acids through the non-covalent interactions would facilitate the formation of either supramolecular helices, sheets, or turns. However, serendipitously, an encapsulated water wire was formed while the molecule was crystallized (Fig. 1c). The same molecule has been studied earlier in single crystals in both of its enantiomeric forms, Boc-γ^4^(*R*)Phe-OH and Boc-γ^4^(*S*)Phe-OH by Jędrzejczak and co-workers (Jędrzejczak et al. 2017). Gopi and co-workers reported the single-crystal structure of the zwitterionic form of γ^4^Phe amino acid which also like the modified γ^4^Phe enantiomers did not have any pore or channel structures in their crystals (Jadhav et al. 2013). These crystal structures do not contain any co-crystallized water or any other solvents and have a very tightly packed structure. We have collected single crystal X-ray diffraction (SCXRD) datasets for Boc-γ^4^(*R*)Phe-OH, at different temperatures to access the temperature-depended features of the hydrate crystals, especially beyond room temperature. We have also performed natural bonding orbital (NBO) analysis with all the SCXRD data sets collected at different temperatures. This would help to do a quantitative analysis of the intermolecular interaction energies involving the water wire in different directions, at different temperatures, in the crystals.

## Materials and methods

*Synthesis*: We have purchased ~100 mg of Boc-γ^4^(*R*)Phe-OH also known as (4R)-4-(tert-butoxycarbonylamino)-5-phenyl-pentanoic acid, in solid powder form from ChiroBlock GmbH, with known impurities content of <2%. 1H-NMR and QC MS spectra of the compound supplied by ChiroBlock are provided in a separate pdf file, mentioned as the certificate of analysis for BGPHEOH.

*Single-crystal XRD*: The solid powder of Boc-γ^4^(R)Phe-OH was used to set up for crystallization, where ~10 mg of the compound was dissolved in 500 μL of methanol and then a few drops of water were added to the solution. The solution was then left undisturbed for 3–4 weeks for the crystals to grow by slow evaporation of the solvents, inside a fume hood. The crystals obtained by the above-mentioned method were then utilized to collect X-ray diffraction data sets on undulator synchrotron radiation with *λ* = 0.61990 Å at P11 beamline in PETRA III, DESY, Hamburg, Germany. First, we collected only a dataset at 80 K for a crystal taken from the same batch of crystals. The structural features observed in the same prompted us to go for SCXRD data collection at room temperature and beyond. The variable temperature SCXRD measurements were performed at different temperatures, starting from 296 to 365 K, first at 10 K intervals with a 2nd crystal and then at 1–2 K intervals with a 3rd crystal. The ramping rate of the temperature was 360 K/hour. The indexing of the X-ray diffraction pattern, unit cell refinement, and spot integration was performed with the XDS program (Kabsch 1993). The crystal structures were solved and subsequently refined using the X-ray diffraction datasets collected at different temperatures. All the X-ray diffraction data sets were collected in phi scan type mode. All the crystal structure was solved using direct methods in SHELXS (Sheldrick 1997a). The structures were refined against F^2^ isotropically, followed by full matrix anisotropic least-squares refinement using SHELXL-97 (Sheldrick 1997b). Except for the hydrogen atoms belonging to the water molecules, mostly, the rest of the hydrogen atoms were fixed geometrically, in idealized positions, and allowed to ride with the respective C or N atoms to which each was bonded, in the final cycles of refinement. CCDC deposition numbers for the compounds are 2239361 (BGPHE1_80K), 2231852 (BGPHE2_RT), 2231853 (BGPHE2_305K), 2231854 (BGPHE2_315K), 2231855 (BGPHE2_325K), 2231856 (BGPHE2_335K), 2231857 (BGPHE3_335K), 2231858 (BGPHE3_337K), 2231859 (BGPHE3_337K_2), 2231860 (BGPHE3_339K_2), and 2231861 (BGPHE3_340K_2) which contain the supplementary crystallographic data for this paper and can be obtained free of charge from The Cambridge Crystallographic Data Centre via www.ccdc.cam.ac.uk/data_request/cif. Crystal data and structure refinement parameters for all the datasets collected at different temperatures are listed in Tables S1–S5.

*Natural bonding orbitals calculation*: The Natural Bond Orbital (NBO) method (Glendening et al. 2012) has been utilized to analyze the intermolecular interactions with a focus on the water wire. The NBO calculations were performed using the geometries obtained from single-crystal XRD measurements. The NBO calculations are carried out in Gaussian16 (Frisch et al. 2016) at M062X/6-311G** (Zhao and Truhlar 2008) level of theory. Tables S9–S10 list the details of the outcome from the NBO analysis of BGPHEOH with single-crystal XRD data collected at different temperatures.

## Results and discussion

**Fig. 2:**
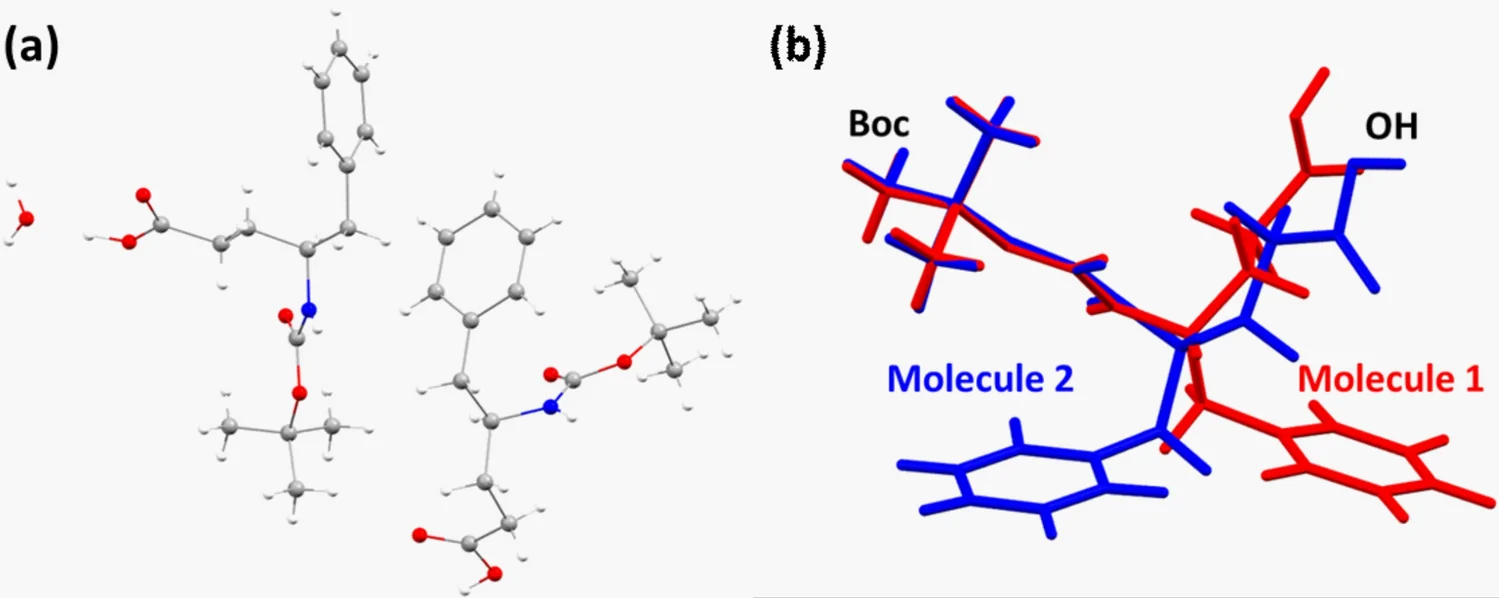
**a** Crystallographic asymmetric unit of BGPHEOH crystals at 80 K. **b** Superposition of the two co-crystallized molecules in the asymmetric unit

The SCXRD dataset of the BGPHEOH crystal at 80 K was collected first, which showed that the BGPHEOH is crystallized in the monoclinic space group *P*2_1_ with two BGPHEOH molecules and a co-crystallized water molecule in the crystallographic asymmetric unit (Fig. 2a). The backbone conformation of the two symmetry-independent molecules was quite different. While, molecule 1 adopts *gauche*^*—*^*-gauche*^*—*^*-trans-gauche*^*—*^ (*g-, g-, t, g-*) conformation about the N–C^γ^ (*φ*), C^γ^–C^β^ (*θ*_1_), C^β^–C^α^ (*θ*_2_), and C^α^–CO (*ψ*) bonds with the values of −80.63°, −64.27°, 177.07°, and −55.13°, for molecule 2 the corresponding values were −133.23°, 64.14°, 89.72°, and −172.06°. The difference in their relative backbone conformation was also quite evident in the superposition of the two molecules presented here (Fig. 2b). While *gauche* (*g*), *gauche* (*g*) conformation for θ_1_ and θ_2_ observed in molecule 2 suggests a helical conformation, in molecule 1 it is in *gauche–*(*g-*), *trans* (*t*+) semi-extended conformation, suitable for *β*-sheet-like structures (Reddy et al. 2015).

**Fig. 3:**
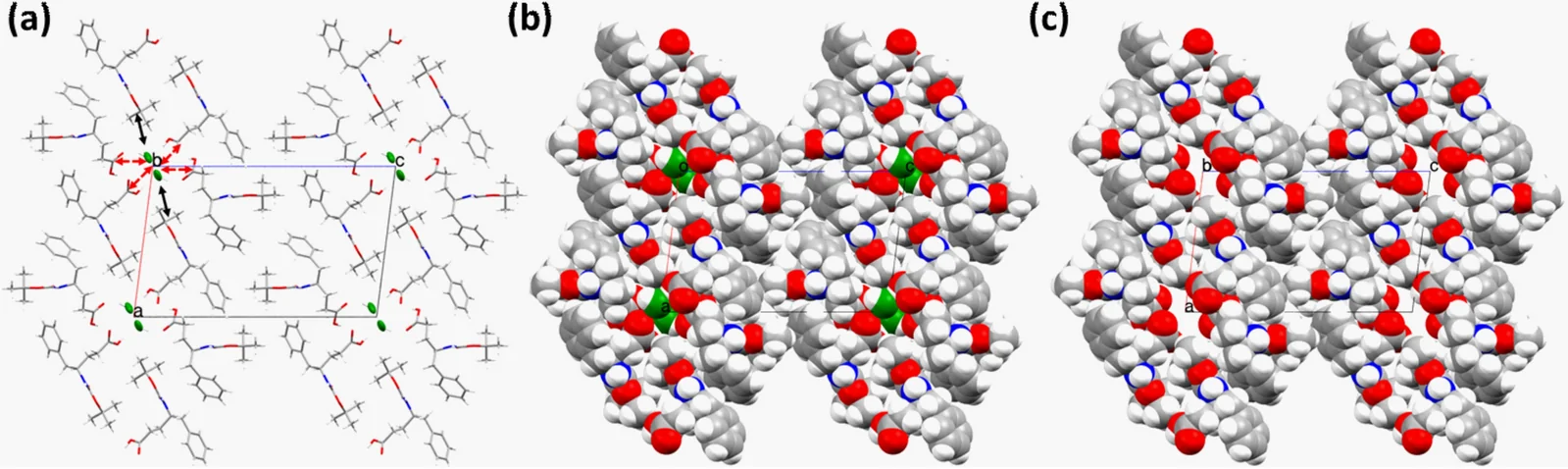
A view down the crystallographic ‘*b*’ axis showing the directions of interactions between the channel wall formed by the amino acids and the water wire. **a** The directions corresponding to van der Waals interactions are highlighted in *black arrows*, whereas the hydrogen bond interactions, are shown in *red dashed arrows*. **b** A space-fill model of Fig. 2a with the water wire is shown in *green*. **c** A space-fill model of Fig. 2a without the water wire, clearly shows the nanochannel formation along the 2_1_-screw axis (color figure online)

The intermolecular hydrogen bond network involving the modified amino acids and water molecules stabilized the entire packing in the crystals. The water wire, formed by the hydrogen-bonded chain of water molecules along the 2_1_-screw axis within hydrophilic channels in the crystals, also interacts with the amino acids, giving an impression of a zipper-like structure when looked along the crystallographic ‘*a*’ axis (Fig. 1c). The impression of a zipper within a channel may sound contradictory but if we investigate the intermolecular hydrogen bonds that stabilize the packing in directions perpendicular to the ‘*b*’ axis, along the ‘*c*’ axis the waters forming hydrogen bonds with the C-terminus of the amino acids that form the inner wall of the channel in the respective directions (Fig. 3a). While in the other direction, mostly along the axis ‘*a*’, the interaction between the inner wall of the channel and the water chain can be considered van der Waal’s interaction where the *tert*-Boc group, protecting the N-terminus of the γ^4^(*R*)Phe amino acids, are protruding inward to form the inner wall of the channels (Fig. 3a). As the packing pattern of the molecules in the crystal suggests, the co-crystallized water molecule played a key role in holding the entire crystal structure. Since along the axis ‘*b*’ and ‘*c*’, it takes part in crucial intermolecular attractive interactions. Therefore, it was perceived that the removal of the water wire from the nanochannel would create a void big enough to disrupt the hydrogen bond network formed by the formation of the water wire in the crystals (Fig. 3b, c). At 80 K, the distance between the successive oxygen atoms in the water molecules in the water wire was 3.13 Å which is quite perfect for considering a strong hydrogen bond in terms of a donor–acceptor distance.

As mentioned earlier, the presence of co-crystallized water molecules in BGPHEOH crystals was absent in an earlier report which analyzed the crystal structure of both Boc-γ^4^(*R*)Phe-OH and Boc-γ^4^(*S*)Phe-OH at 100 K (Jędrzejczak et al. 2017). The melting point for the Boc-γ^4^(*R*)Phe-OH was found to be 122–124 °C, in that report. Although with opposite signs, the backbone torsion angles for γ^4^Phe in both *R* and *S*-configuration have a *gauche*, *trans* (*g*, *t*) conformational combination for the θ_1_ and θ_2_ torsion angles. Whereas, in our present report, both *gauche, trans* (*g, t*) and *gauche, gauche* (*g, g*) conformations were observed for the same set of torsion angles. The difference in the backbone torsion angles is also accompanied by the incorporation of a co-crystallized water molecule in the asymmetric unit and the crystallographic space group symmetry which was *P*2_1_2_1_2_1_ in the previously published report for Boc-γ^4^(*R*)Phe-OH, is *P*2_1_ in the present study.

**Fig. 4:**
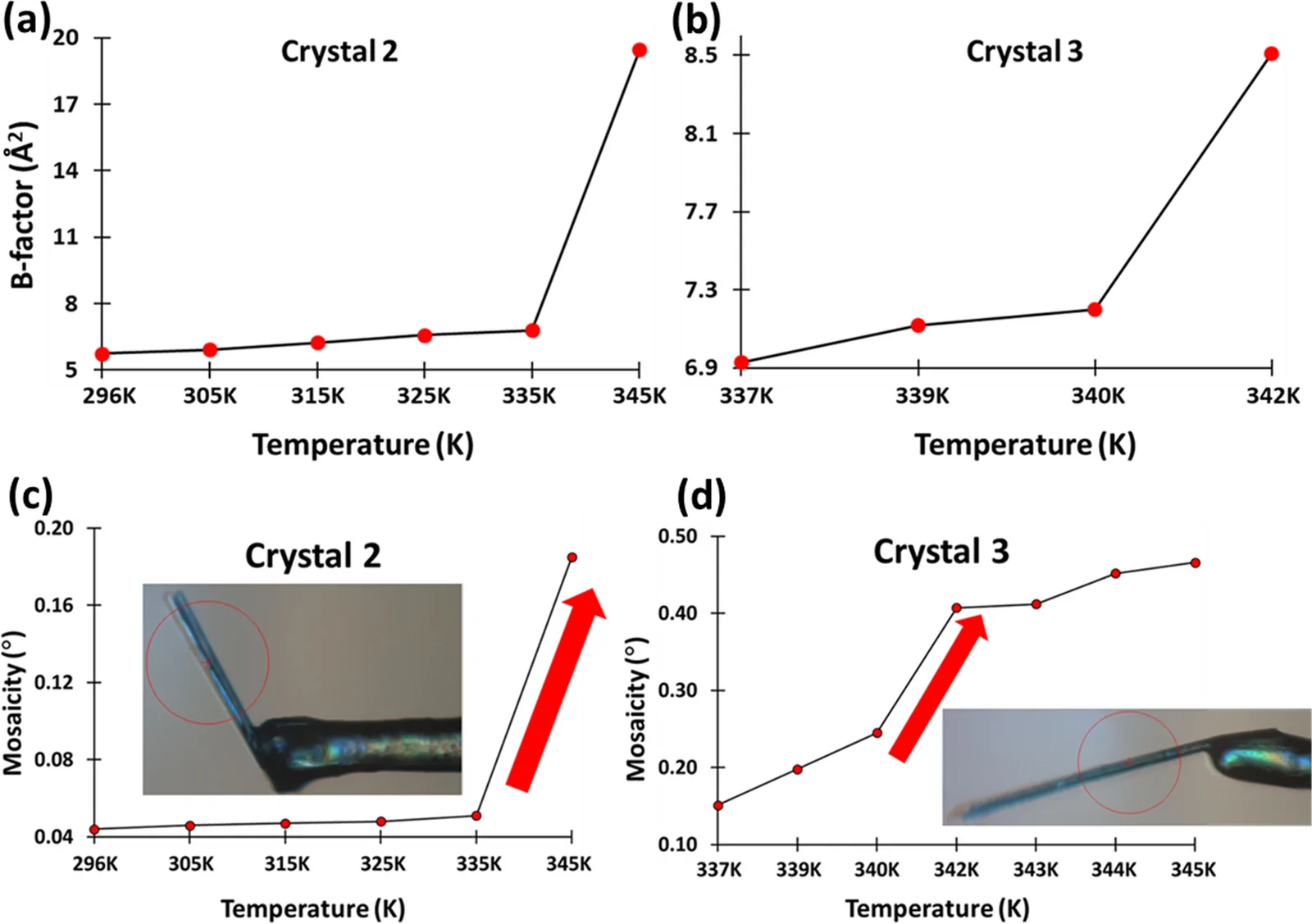
Temperature vs. Debye–Waller B-factor obtained from SCXRD studies for **a** crystal2 and **b** crystal3. Temperature vs. mosaicity obtained from SCXRD studies for **c** crystal2 and **d** crystal3

The intermolecular hydrogen bond network in the crystals of BGPHEOH at 80 K encouraged us to investigate the zipper-like network formed by the hydrogen-bonded water wire at different temperatures. The variable temperature SCXRD measurements were performed with two different crystals from the same batch of crystallization setup. The 2nd crystal, which we denoted as “crystal 2” from this point, was used to collect the SCXRD data starting from 296 K up to 365 K with 10 K intervals and then went back to 296 K. As we increase the temperature by approximately 10 K from 296 K, the Debye–Waller factor (B) and the mosaicity of the crystal increase gradually up to 335 K (Fig. 4a and c). But as we move from 335 to 345 K, both parameters have increased drastically suggesting a 1st order phase transition (Fig. 4a and c). A gradual decrease in the spot intensity as well as the disappearance of some of the high-resolution spots was also observed as we went up in temperature (Figure S1). At 345 K, some of the diffraction spots still remain well separated and circular-shaped, though the indexing of the diffraction pattern for the first time suggested a triclinic unit cell, with a slight change of the dimensions (Table S3). Although it could be also possible to force the indexing into a monoclinic unit cell, as the unit cell dimensions suggest (Table S3). Moreover, the unit cell volume remains more or less the same only keeping up the same trend of gradual increment with increasing temperature as expected in the absence of any other transition or solvent evaporation (Tables S2 and S3). At 355 and 365 K, the diffraction patterns, along with well-separated circular spots also contain semi-circular arcs suggesting the collapse of the crystalline order in a particular direction (Figure S1). While reverting to the 296 K, the condition does not improve, suggesting an irreversible change impacted the crystal lattice at 345 K. There was no evidence of a single-crystal-to-single-crystal transition within the above-mentioned temperature range. Nonetheless, all the measurements up to 345 K suggest no significant change in the unit cell volume inferring that the water molecules were still present in the lattice (See the column of unit cell volume in Tables S2–S5).

Finally, a 3rd crystal was mounted from the same batch and will be denoted as “crystal 3” from this point onward, to carefully collect SCXRD datasets beyond 335 K with 1–2 K intervals in temperature. We were aware that the transition temperature may vary from crystal to crystal even if they are taken from the same batch of crystallization setup and have the same crystallographic symmetries and more or less the same unit cell dimensions and morphology. After mounting crystal 3, the SCXRD datasets at 335 and 337 K were collected but due to the irregular diffraction spot shapes, a different portion of the same crystal was focused for data collection. Since we did not observe the appearance of any line or ring structures on the diffraction patterns due to the increased mosaicity caused by the increased temperature at 337 K (Mosaicity at this point was 0.232°), we decided to continue the gradual increment of temperature, starting with 337 K, with this new focusing region on the same crystal. We have collected the SCXRD datasets at 337, 339, 340, and 342 K but while moving from 340 to 342 K, the worsening of the crystallinity was indicated by the appearance of a number of distorted spot shapes and ring structures, along with some existing well-separated and circularly-shaped spots (Figure S2). This also marked the transition temperature since the mosaicity and Debye–Waller (B) factor at 342 K showed a drastic increment in values compared to 340 K (Figs. 4b and d). Subsequently, when we moved to 343, 344, and 345 K, high-resolution spots start either disappearing or their intensity becomes so low that they become difficult to notice (Figure S2). Moreover, well-separated spots are largely replaced by long straight/curved lines, a feature corresponding to the polycrystalline nature of the crystals that starts appearing. The diffraction pattern remained the same while reverting the temperature to 295 K. While the faint rings are evidence of a complete collapse of periodicity in certain directions with respect to specific Miller planes, the line patterns remain a constant feature, suggesting while the periodicity may have collapsed in one direction, it is certainly maintained in another direction within the crystal lattice. The crystalline order or the 3D periodicity of the single crystals has irreversibly changed due to the gradual increment of temperature beyond 340 K temperature, in crystal 3. The introduction of this partial collapse of the crystal lattice which resulted due to the compromised periodicity in particular directions as a result of the gradual increment of temperature can be analyzed closely in terms of the intermolecular interactions that stabilize the packing of molecules in the first place.

It is important to note that the overall packing and intermolecular interactions that allow the formation of water wire in hydrophilic channels to provide the zipper-like structures remain quite similar in all the crystal structures that could be solved, evidently only up to 340 K, with crystal 3. Throughout this range, the space group symmetry remained unchanged. The backbone torsion angles and the hydrogen bond parameters for all the intermolecular hydrogen bonds also more or less remain the same at different temperature points, across the crystals (Table 1, S6, S7, S8). The average backbone torsion angle values for all the structures across the temperatures and crystals are *φ* = (−79.04 ± 1.60)°, *θ*_1_ = (−64.56 ± 1.99)°, *θ*_2_ = (177.27 ± 1.17)°, *ψ* = (−60.46 ± 3.16)° for molecule 1 and *φ* = (−131.11 ± 1.04)°, *θ*_1_ = (65.10 ± 1.03)°, *θ*_2_ = (91.24 ± 1.03)°, *ψ* = (−175.00 ± 1.40)° for molecule 2 (Table 1). The backbone torsion angles *θ*_1_ and *θ*_2_ in molecule 2 adopted the values that are quite close to the helical conformation *gauche*, *gauche* usually adopted by γ-residues in (αγ)_*n*_ (Vasudev et al. 2011) or (γγ)_*n*_ helices (Basuroy et al. 2013b). Whereas, molecule 1 adopted a *gauche*, *trans* conformation about the same set of bonds which is mostly adopted by γ-residues incorporated in short extended peptides that do not form any intramolecular hydrogen bonds (Reddy et al. 2015). If we closely look at the intermolecular hydrogen bonds formed at different temperatures then we can see that from 296 to 325 K, there were only two hydrogen bond interactions where the water molecule took part as the donor whereas from 335 K onward to 340 K, the number was three (Figure S3, Table S7).

**Table 1:** Backbone torsion angles of the **γ**^**4**^**(*R*)Phe** residues obtained from the single crystal X-ray diffraction measurements performed at different temperature points with different single crystals

Datasets	Backbone torsion angle of the **γ**^**4**^**(*R*)Phe** residues			
	(C0-N1-C1G-C1B) *φ*(°)	(N1-C1G-C1B-C1A) *θ*_1_(°)	(C1G-C1B-C1A-C1) *θ*_2_(°)	(C1B-C1A-C1-O2) *ψ*(°)
*Molecule 1*				
Crystal 2				
BGPHEOH2_296K	−81.06	−64.04	177.37	−57.68
BGPHEOH2_305K	−80.43	−64.89	177.98	−57.78
BGPHEOH2_315K	−79.97	−64.35	178.07	−59.34
BGPHEOH2_325K	−79.71	−64.34	178.56	−60.93
BGPHEOH2_335K	−78.15	−63.95	178.14	−63.27
*BGPHEOH2_345K dataset was collected but was not good enough to get a structure*				
Crystal 3 position 1				
BGPHEOH3_335K	−77.87	−65.20	178.32	−61.75
BGPHEOH3_337K	−76.81	−69.43	176.34	−57.80
Crystal 3 position 2				
BGPHEOH3_337K_2	−77.13	−64.46	177.35	−62.81
BGPHEOH3_339K_2	−77.92	−64.09	176.37	−62.09
BGPHEOH3_340K_2	−76.66	−65.03	177.49	−63.19
*BGPHEOH3_342K_2 dataset was collected but was not good enough to get a structure*				
Molecule 2				
Crystal 2				
BGPHEOH2_296K	−131.82	64.80	89.69	−173.22
BGPHEOH2_305K	−130.80	63.93	91.52	−174.37
BGPHEOH2_315K	−130.78	64.13	91.00	−174.04
BGPHEOH2_325K	−131.78	66.14	90.18	−174.47
BGPHEOH2_335K	−130.48	65.55	91.36	−176.31
Crystal 3 position 1				
BGPHEOH3_335K	−131.79	66.67	90.66	−175.28
BGPHEOH3_337K	−132.35	62.90	93.56	−176.43
Crystal 3 position 2				
BGPHEOH3_337K_2	−130.72	66.00	91.04	−176.29
BGPHEOH3_339K_2	−130.99	65.52	91.43	−175.98
BGPHEOH3_340K_2	−131.64	66.47	90.65	−176.52

**Fig. 5:**
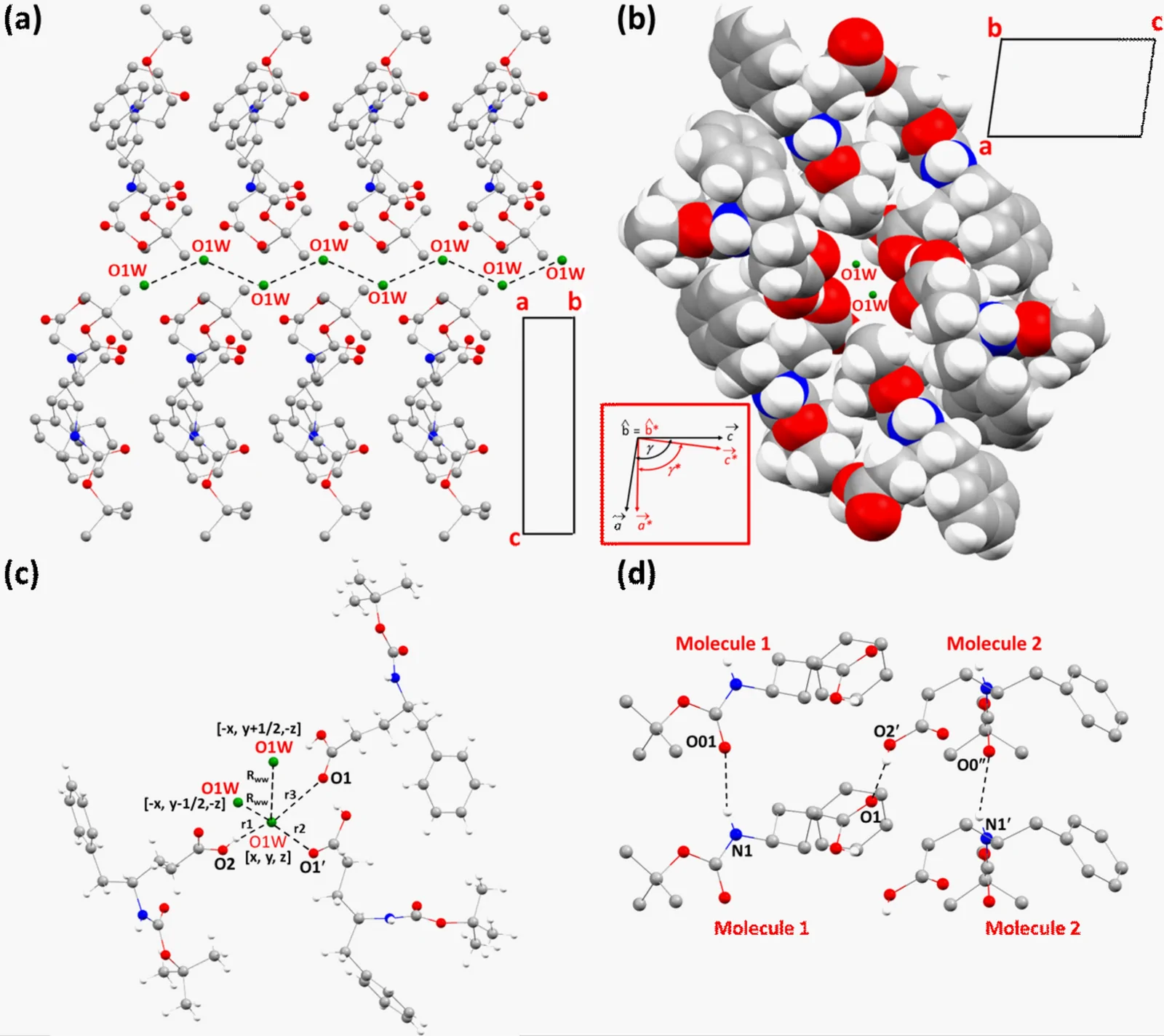
**a** A view of the chain of water molecules within the single crystals of BGPHEOH. **b** A view along the crystallographic ‘*b*’ axis showing the water wire formation in a hydrophilic channel. **c** Intermolecular interactions involving a water molecule in the crystals (hydrogens of the water molecule are removed). **d** Intermolecular hydrogen bonds formed between the unit-translated molecule 1’s and unit-translated molecule 2’s and also between the molecule 1 and 2

Figures 5a and b exhibits two perpendicular views of a zig-zag water wire formation along the crystallographic ‘*b*’ axis that coincides with the direction of the signature 2_1_-screw axis of the monoclinic crystal system. Especially, Fig. 5b shows the formation of a water wire within a hydrophilic channel formed by the modified amino acids. It is important to note that the inner wall of the channel is formed by either the N- or C-terminus of the amino acids and the sidechains are not protruding inwards. Figure 5c also provides a closer look at the intermolecular interactions between a water molecule with the amino acid molecules. While, the interactions between the symmetry-independent molecules as well as the interactions between the unit-translated molecules of the same type along the ‘*b*’ axis, were presented in Fig. 5d.

Although the overall trend in molecular conformations and the hydrogen bond parameters throughout all the structures remain similar, it was interesting to have a closer look at the change in the interaction energy values between the molecules with temperatures. In order to know the interaction energies between the molecules keeping a focus on the water wire, natural bond orbital (NBO) analysis at M06-2X/6-311G** level of theory in the gas phase, using the geometries, obtained from X-ray diffraction datasets collected at different temperatures, was performed. The NBO calculations help us to understand the relative strength of various intermolecular interactions in different directions and also allow us to draw a conclusion on the probable site of stress in the lattice due to disorders introduced by gradually increased temperature. Mostly, the NBO analysis provides the donor–acceptor interaction energies from 2nd order perturbation theory analysis of the Fock matrix in NBO basis, with *E*(2) > 0.05 kcal/mol as listed in Tables S9–S10. The interaction energies provide a measure of the strength of the intermolecular interactions.

**Fig. 6:**
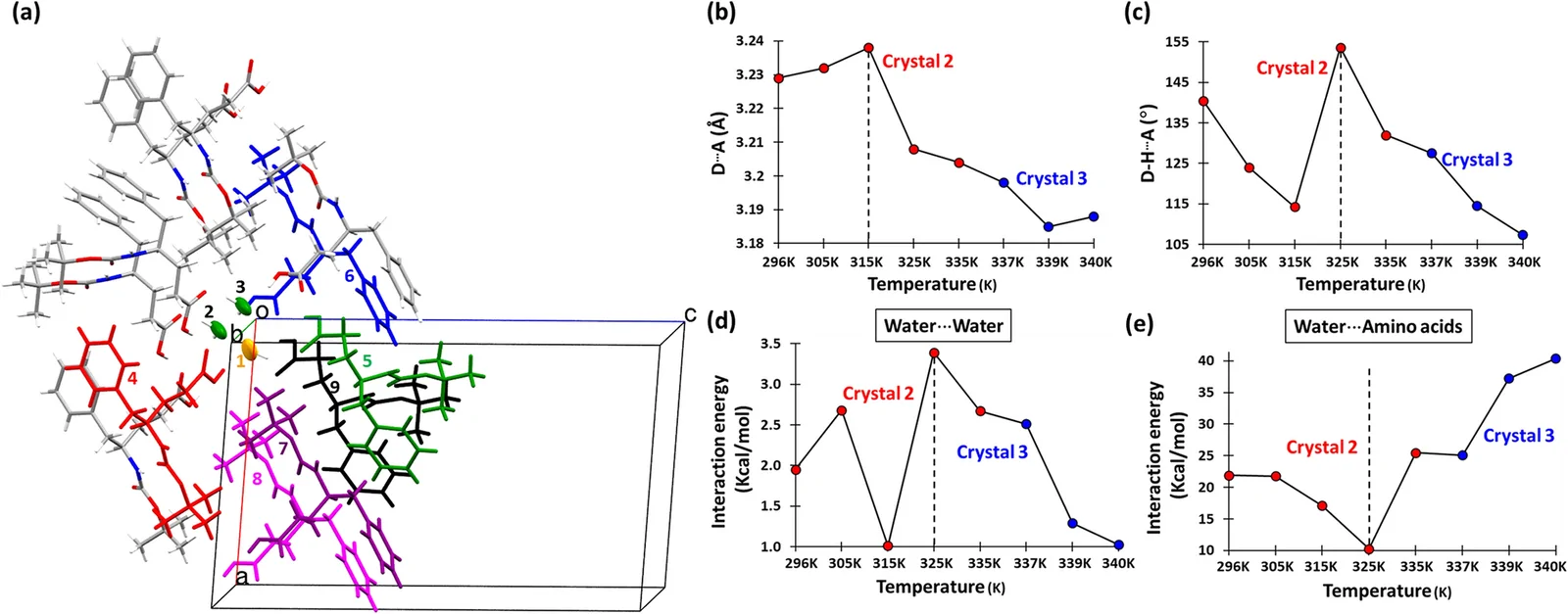
**a** The cluster of 15 molecules chosen from the crystal structures for the NBO calculation to find out the strength of intermolecular interactions (The molecules with significant interactions with the water molecule are painted in *red*, *green*, *blue*, *purple*, and *magenta*) (the oxygen atom of the water molecule of the interest is colored in orange with no. 1). **b** The distance between a hydrogen bond donor and acceptor vs. temperature while water molecules are forming a chain. **c** The donor–hydrogen–acceptor angle vs. temperature for the hydrogen-bonded water molecules in the formation of the water wire. **d** Interaction energies vs. temperature calculated by NBO methods for water–water hydrogen bond interactions. **e** Interaction energies vs. temperature calculated by NBO methods for interactions between a water molecule with the amino acids (color figure online)

The NBO calculation was performed using a cluster of molecules that consists of 3 water molecules and 12 surrounding molecules (Fig. 6a). The molecules having significant interactions with the water molecule of our interest are colored as a whole. The interaction energy between the water molecules and the interactions between the water molecule and the derivative molecules are listed in Tables S9. The water–water interaction is mostly OH⋅⋅⋅O hydrogen bond and by nature a dipole–dipole interaction, is strongly directional and heavily depends on the O–H⋅⋅⋅O/Donor-Hydrogen⋅⋅⋅Acceptor (D–H⋅⋅⋅A) angle which follows a trend as we investigate the structures obtained at different temperatures. As we increase the temperature beyond 296 K, the D⋅⋅⋅A distance starts increasing but after 315 K it shows a steady decrease, whereas the D-H⋅⋅⋅A angle which shows a steady decrease up to 315 K shows a sudden increment at 325 K (Fig. 6b and c). But after 325 K, the D-H⋅⋅⋅A angle value again shows a steady decrement till the end. The interaction energy between the waters also exactly matches the trend of the D-H⋅⋅⋅A angle value from 325 K onward (Fig. 6d). This also proves again the directional nature of the hydrogen bond interactions. Where the shortest D⋅⋅⋅A distance does not have the strongest interaction but the highest D-H⋅⋅⋅A angle value has the strongest interaction between the waters. The interaction energy between a water molecule and the surrounding derivative molecules also showed a trend where beyond 325 K there is a steady increment in the value (Fig. 6e). The interaction energies involving all the molecules along the crystallographic ‘*b*’ axis also do not show any particular trend and only fluctuate about a mean value. The complementary nature of the interaction energy could be explained in terms of the change in the orientation of water molecules within the hydrophilic channel. As more heat is applied, one of the weaker interactions i.e. between the waters gets affected more strongly than the other interactions. This is quite understandable given the size of the water molecules and how easy it is to perturb the orientation of a water molecule than much bigger derivative molecules. With the increment of temperature, the OH groups of the water start orienting more favorably towards the wall of the channel than towards the symmetry-related water molecules. As we mentioned earlier, from 325 K onwards the water molecules started forming three hydrogen bonds as a donor which was only two up to that point (Table S7). This also suggests the change in orientation of the water molecules that takes place beyond 315 K. It is exciting to see that these trends are maintained across the crystals, which is helpful in drawing conclusions regarding the nature of the interactions that we are addressing.

**Fig. 7:**
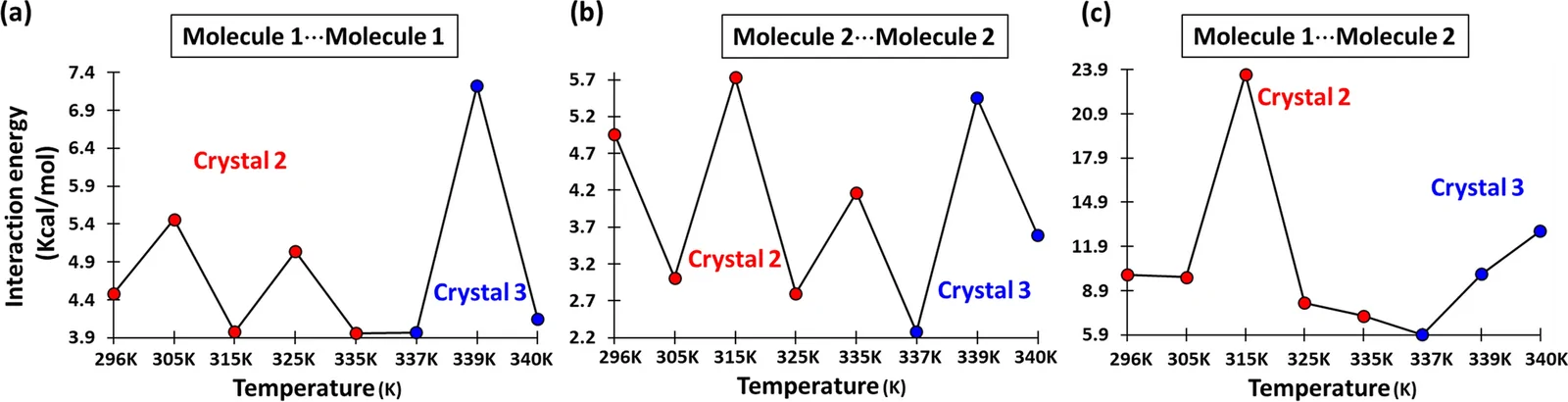
The interaction energy between the unit-translated molecules along the crystallographic ‘b’ axis, **a** Molecule 1⋅⋅⋅Molecule 1; **b** Molecule 2⋅⋅⋅Molecule 2. **c** Interaction energy between the symmetry-independent molecules

The NBO-calculated interaction energies between the symmetry-independent amino acids and also between the unit-translated amino acids belonging to the same type, at different temperatures, are also presented in the form of scatter plots (Fig. 7). Figure 7 exhibits that with temperatures the interaction between the unit translated derivatives along the crystallographic ‘*b*’ axis, more or less remains the same and only shows minor fluctuations about a mean value. Whereas, the interaction energy between the hydrogen-bonded, symmetry-independent pairs fluctuated greatly ranging from 5.93 kcal/mol at 337 K in crystal 3 to 23.59 kcal/mol at 315 K in crystal 2 (Fig. 5c).

**Fig. 8:**
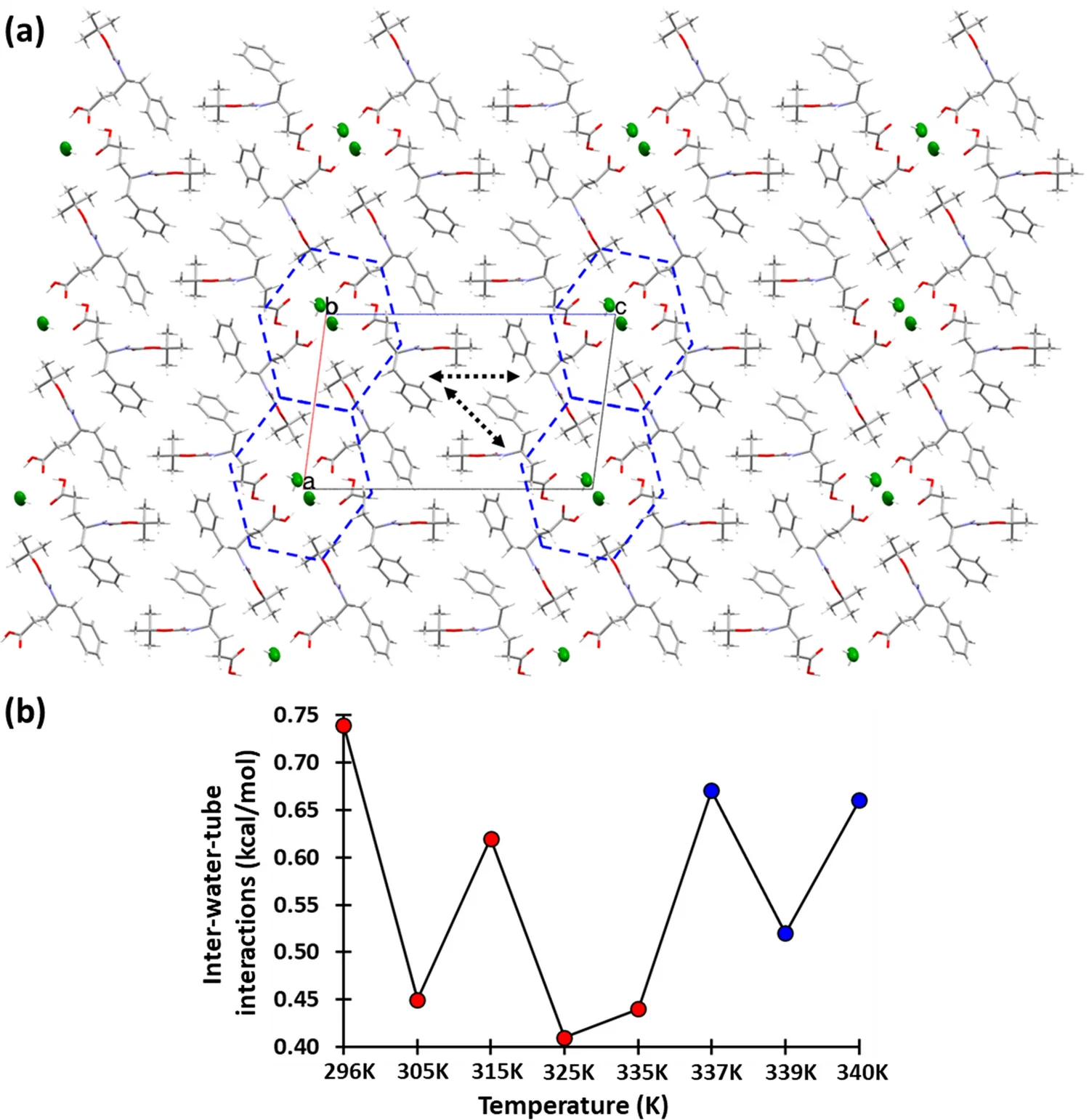
**a** The packing of molecules in BGPHEOH crystals at 80 K showed in a projection along ‘*b*’ axis showcasing the hydrophilic channel that holds the water wire. The honeycomb-like structures with a water molecule surrounded by 6 derivatives are highlighted with *blue-colored dashed lines*. **b** The interaction energy vs. temperature for the interactions between the honey-comb-like structures in the crystals, as pointed out by *two dashed arrows* in Figure **a**, in *blue* (color figure online)

A projection down the crystallographic ‘*b*’ axis shows that the lateral arrangement of the amino acid nanochannels resembles a honeycomb pattern in the ‘*ac*’ plane (Fig. 8a). The interactions between these honeycomb patterns mostly depended on two symmetry-independent interactions as shown by dashed arrows in Fig. 8a. The NBO-calculated interaction energies for the above-mentioned pair of interactions are plotted at different temperatures in Fig. 8b, which shows that very weak interactions of merely 0.40–0.75 kcal/mol energy are responsible for providing the binding energy between the hexagonal-shaped cells for holding this entire honeycomb pattern in the crystallographic ‘*ac*’ plane, along the directions, *a* + *c* and *c*. Whereas, along the direction *a–c*, the honeycomb pattern is tightly held through strong water–amino acid hydrogen bond interactions.

**Fig. 9:**
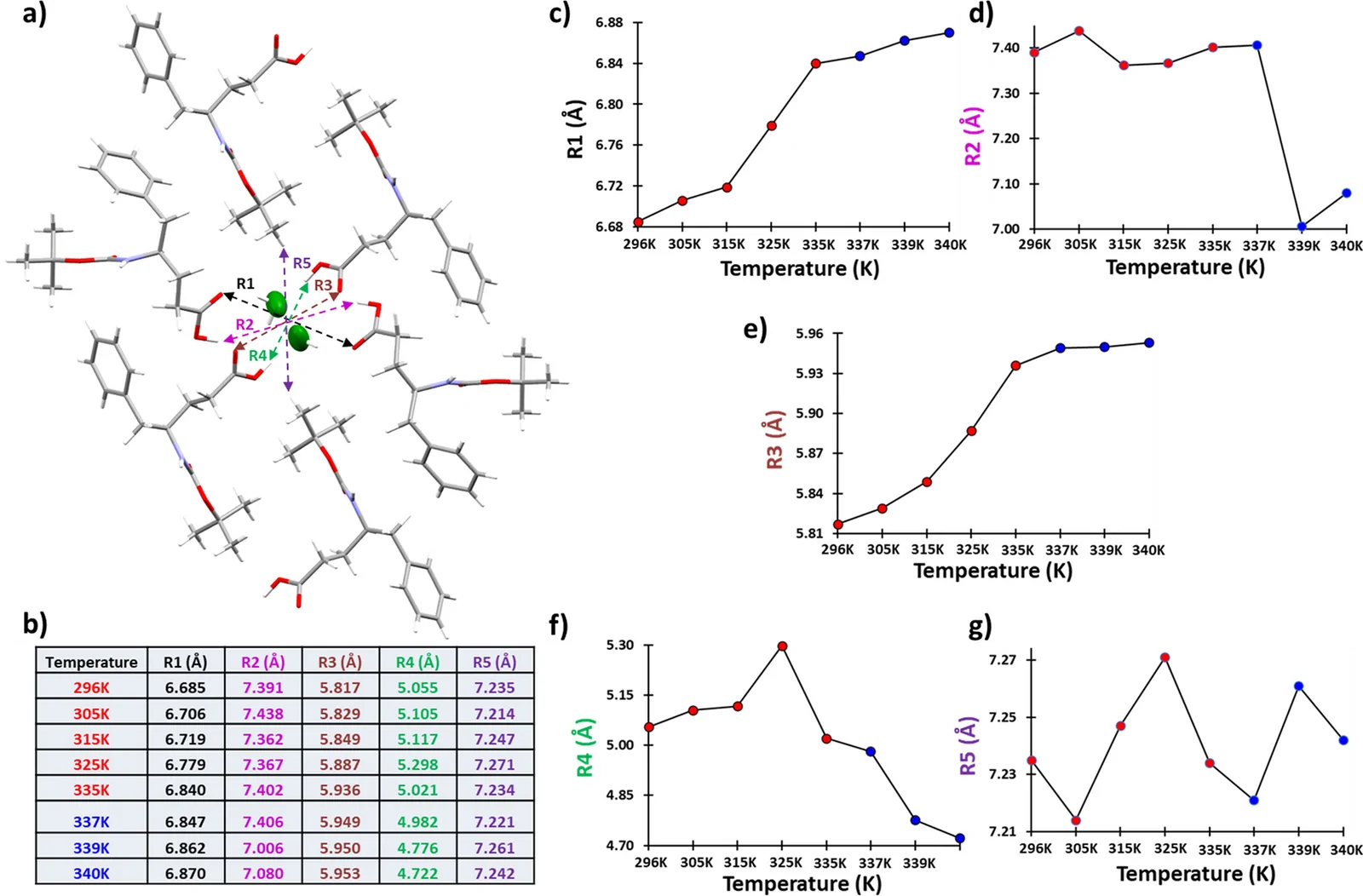
An approximate calculation of the diameter of the hydrophilic channel by checking the inter-atomic distances across the channel cross-section. **a** The interatomic distances indicated that used as a rough measure of the channel diameter. **b** The interatomic distances across the channel at different temperatures obtained from the SCXRD measurements are listed. The *scatter plots* corresponding to the table provided in **b** are exhibited in **c**, **d**, **e**, **f**, and **g**

In the course of our search for a weak link in terms of intermolecular interaction strength, we have also investigated the change in channel diameter with temperatures (Fig. 9a). The channel cross-section has an approximately hexagonal shape and the five interatomic distances across the channel diameter were identified, measured, and tabulated for all the crystal structures belonging to different temperatures (Fig. 9b). The measurement is in no way exhaustive but more like an approximate method of checking the diameter of the nanochannel. While the distances R1 and R3 gradually increase with the temperature, R2 shows a sudden drop beyond 337 K (Fig. 9c, d, e). Whereas, the distance R4 showed a gradual decrement beyond 325 K (Fig. 9f). Only the distance R5 does not show a drastic change in the trend and showed minor fluctuation about a mean distance (Fig. 9g). Overall, the diameter of the channel cannot be said to have a significant change over temperatures up to 340 K.

**Fig. 10:**
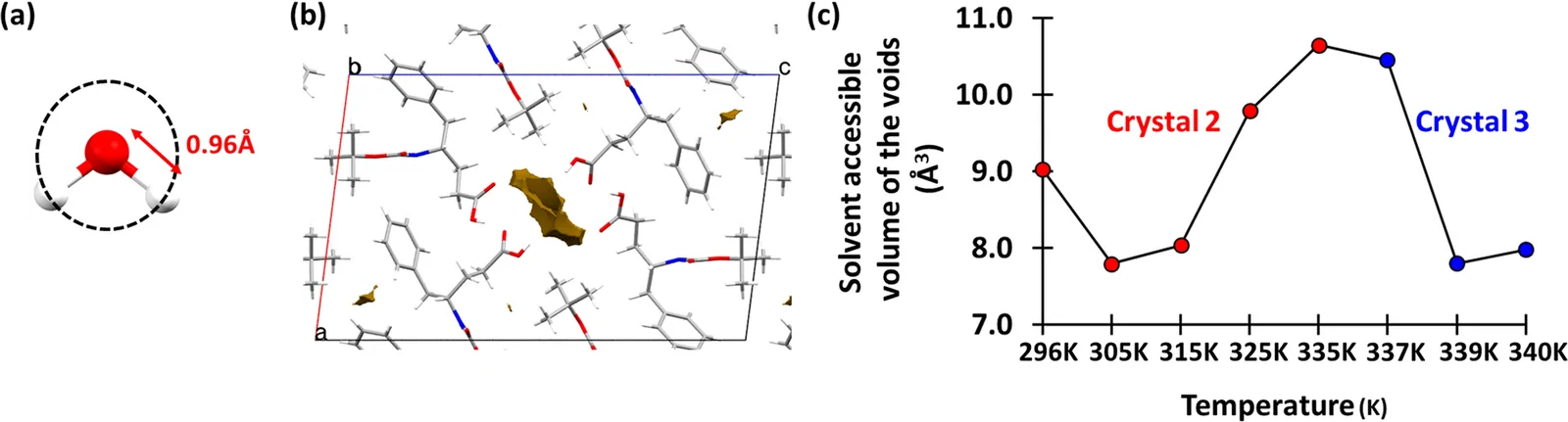
**a** The spherical probe of radius 0.96 Å where 0.96 Å is the average O–H bond length of the water molecules. **b** The solvent-accessible voids that would accommodate a spherical probe with a 0.96 Å radius are shown for the 296 K SCXRD dataset. **c** The *scatter plot* shows the volume of the solvent-accessible void within the unit cell at different temperatures

Apart from the above-mentioned channel size analysis at different temperatures, we have also calculated the solvent-accessible surface volume within the unit cell at different temperatures by using the Mercury 2020.2.0. We have searched for the void within the unit cell that would accommodate a spherical probe with a radius of 0.96 Å, which is the average H–O bond length for the water molecules. The search for a void at different temperature crystal lattice also provided the percentage of the unit cell volume that the void would have and the actual volume of the void (Table S10). For this analysis, we have moved the lattice on the *ac* plane in such a way that the water wire-containing channel sits at the center of the unit cell. The overall results suggested no particular trend with either gradually increasing or decreasing values of the volume of the void available for solvent accessibility, with the temperatures (Table S10; Fig. 10).

After a thorough analysis of the NBO-calculated interaction energies between the several pairs of molecules in the cluster of 15 molecules in different directions, it can be said that the inter-cell interactions in the honeycomb pattern in the *ac* plane along the crystallographic ‘*c*’ axis and along the direction *a* + *c* can be considered are the weakest amongst the lot. Therefore, it is highly likely that the increment temperature beyond a certain point would affect these interactions first and would cause the degradation of the crystalline order in the crystals. The intermolecular interaction energy between the symmetry-related water molecules is also amongst the weaker ones and ranges somewhere between 3.39 kcal/mol at 325 K and 1.01 kcal/mol at 315 K. But the water–water hydrogen-bonded chain is also placed parallel to the much stronger N–H⋅⋅⋅O interaction between the unit-translated amino acids along the crystallographic ‘*b*’ axis. Therefore, the impact of the change in hydrogen bond strength between the waters would not be significant enough to disrupt the crystalline order along the crystallographic ‘*b*’ axis. Moreover, the intermolecular interaction energy along axis ‘b’ shows that although the energy values fluctuate a lot in the temperature range 337–340 K, there was no specific trend was observed (Figure S3). Although one can argue that at 340 K, the intermolecular interaction energy strength along the ‘*b*’ axis was to lowest with 8.75 kcal/mol and it could be that from this point onwards it takes a big dip which eventually disrupts the periodicity in the crystal in a particular direction. Especially, when the interactions between the unit-translated amino acids along the ‘*b*’ axis are also showing a drop after 339 K. Therefore, it is safe to say that the strong sign of degradation of the crystalline order that was observed starting from 345 K in crystal 3, could be either due to the weakening of intermolecular interaction along the ‘*b*’ axis or due to the impact of heating on the already weak interactions between the hexagonal cells of the honeycomb pattern along the ‘*c*’ axis and the direction *a* + *c*.

We would also like to draw a parallel between the nanochannel formed in the present study with a zwitterionic dipeptide structure presented by Görbitz (2001), where two water molecules are co-crystallized with two peptide molecules in the crystallographic asymmetric unit. The low symmetry space group of the compound, named LF (Leu-Phe) has allowed forming a nanotube with two parallel chains of water molecules within a non-uniform pore of variable diameter. The Leu-Phe dipeptide was also crystallized in a monoclinic space group *P*2_1_. There was also the pore was not uniform in terms of not having a single average pore diameter but rather a range from ∼5.4 to ∼7.4 Å. The inner wall of that nanotube is also hydrophilic in nature which forms hydrogen bonds with the water chains.

## Conclusions

The encapsulation of a water wire in a channel is quite unique in the context of a self-assembled nanostructure in crystals of modified γ-amino acids. Apart from their superior proteolytic stability, the greater conformational degrees of freedom compared to their α-counterparts, enables the self-assembling γ-amino acids with a better opportunity to tune the channel cross-section size and shape in order to modify the physical and chemical properties of the porous structures for suitable biological and non-biological applications.

The presence of strong hydrogen bond acceptor group C = O in the *tert*-Boc and strong hydrogen bond donor groups NH and OH facilitate strong intermolecular hydrogen bonds between the BGPHEOH molecules when they are packed in close proximity and forms single crystals with 3D periodicity. The modified γ-amino acids are usually known to adopt *gauche*, *trans* conformation about the C^γ^–C^β^ (*θ*_1_) and C^β^–C^α^ (*θ*_2_) bonds, respectively, while forming parallel/anti-parallel *β*-sheet structures (Reddy et al. 2015). But in the present study, one of the two symmetry-independent molecules in the crystallographic asymmetric unit has adopted helical *gauche*, *gauche* conformation about the C^γ^–C^β^ (*θ*_1_), and C^β^–C^α^ (*θ*_2_) bonds, respectively. Although a projection down the crystallographic ‘*b*’ axis would show that the water wire was encapsulated inside a nanochannel, the water wire is strongly hydrogen bonded with the inner wall of the channel from two opposite directions, in a zipper-like configuration, while the interactions in other two directions are only of van der Waals in nature.

While the appearance of line and ring patterns in the 355 and 365 K datasets certainly suggest the loss of periodicity either in certain directions or in a certain domain, the unit cell volumes at all these temperatures more or less remained the same, suggesting the water molecules still remained in the lattice till 365 K. Although, it is quite possible that the intermolecular hydrogen bond may have been stressed and also possibly partially disrupted due to the increased lattice temperature. The volume of the solvent-accessible void within the unit cells also suggests that up to 340 K, there was no appreciable change. The NBO-calculated intermolecular interaction energies at different temperatures gave crucial insight into the change in interaction energies between the channel wall and the water wire. The results showed the change in the orientation of the water molecules in the water wire with temperatures which plays a crucial role in the intermolecular hydrogen bond interactions owing to the directional nature of it. As we go higher in temperature the change in orientation of the water molecules prefers the zipper interactions over the water–water hydrogen bond interactions. This was also reflected, since 325 K onward the water molecules in the wire started forming more hydrogen bonds as donors.

The distance and angles regarding the intermolecular hydrogen bonds between the water molecules in Fig. 6 suggest a change in terms of D**⋅⋅⋅**A distances from 315 K but for D-H**⋅⋅⋅**A angles the changes are from 325 K. Whereas, Fig. 4 shows the change in overall mosaicity and the Debye–Waller B-factor with temperature. This also proves that the drastic change in those two parameters is not entirely dependent on the stability of the water wire inside the channel. It also suggests that while the integrity of the water wire started going away slowly after 325 K, the sudden collapse of the overall order of the crystal happens after 340 K. Therefore, the integrity of the channel was not entirely dependent on the integrity of the water wire as long as the water molecules still remain in the crystal lattice. This shows that this crystal structure is not only held together by the hydrogen bonding between the waters of hydration, as we believed at the beginning of the study.

Overall, the idea was to study the intermolecular interactions involving the water wire up to the point at which structure calculation from X-ray diffraction was not feasible at all due to deteriorating crystalline order by increased temperature. The study allowed us to provide a detailed picture of the temperature-dependent dynamics of the encapsulated water wire within the hydrophilic nanochannel of a self-assembling acyclic, modified γ-amino acid crystal, which however, we feel necessary in order to utilize these amino acid nanochannels for any serious application in the field of designing tunable nanostructures with desired biochemical or biophysical properties.

## Supplementary Information

Below is the link to the electronic supplementary material.

Supplementary file1 See the certificate of analysis for BGPHEOH for 1H-NMR and QC-MS spectra of BGPHEOH, as supplied by ChiroBlock GmbH. The crystal data and structure refinement parameters, conformational analysis of the molecular structures, intermolecular hydrogen bond parameters, packing of molecules in single crystals, and NBO calculation results are provided in the supplementary file (PDF 328 KB)

Supplementary file2 (DOCX 2682 KB)

## Data Availability

The supplementary crystallographic data presented in this study are openly available and can be obtained free of charge from The Cambridge Crystallographic Data Centre via www.ccdc.cam.ac.uk/data_request/cif by using the CCDC numbers provided in the main text.
