# Supplementary material for: Investigation of encapsulated water wire within self-assembled hydrophilic nanochannels, in a modified γ4-amino acid crystals: Tracking thermally induced changes of intermolecular interactions within a crystalline hydrate
Source: Amino Acids. 2024 Feb 5;56(1):9. doi: 10.1007/s00726-023-03372-4 (PMC10844418; doi:10.1007/s00726-023-03372-4)

Certificate of Analysis

Chemical Name: (4R)-4-(tert-butoxycarbonylamino)-5-phenyl-pentanoic acid

Chemical Structure:

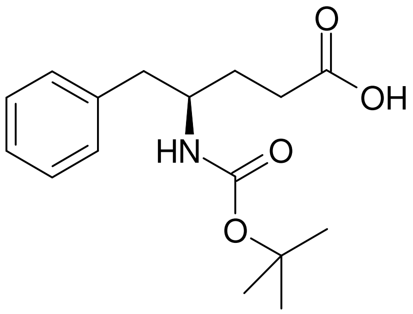

Formula: $C_{16}H_{23}NO_4$

M.W. [g/mol]: 293.37

**Product Work Name**	-
**ChiroBlock Product Number**	-
**Version Control Number of Manufacturing Procedure**	-
**ChiroBlock Lot No.**	UDY 4718 w1 III
**Date of Lot Manufacturing**	01.04.2019
**Date of Lot Testing**	02.04.2019
**Date of Lot Re-testing**	02.04.2021

ChiroBlock GmbH®
Amtsgericht Stendal
HRB 16 169
Ust.-IdNr.: DE 812 756 800

Geschäftsführer:
Dr. Oliver Seidelmann
Dr. Volkmar Wendisch

Tel.: +49 (0) 3494/63 83 23
Fax: +49 (0) 3494/63 83 24
e-mail: contact@chiroblock.de
www.chiroblock.de

Adresse:
Andresenstr. 1a
06766 Wolfen
Germany

BIC: HYVE DE MM462
IBAN: DE 968 002
00 870 603 953 010

Parameter	Analytical Principle & Method[1]	Specified Result	Experimental Result	Unit
Appearance	Visual inspection	n.a.	white solid	n.a.
Identity	MS (ESI, pos. mode)	conforms to structure	316 [M+Na]+, 194 [MH-BOC]+	m/z
Identity	^{1}H-NMR	conforms to structure	conforms to structure	ppm
Purity	^{1}H-NMR	known impurities < 2	no known impurities	mass-% by integral

Wolfen, 03.04.2019

A. Müller
Quality Control

[1] If the method is not specified in detail, ChiroBlock's standard methods apply (derivable from the analytical raw data [file] referenced in the project records).

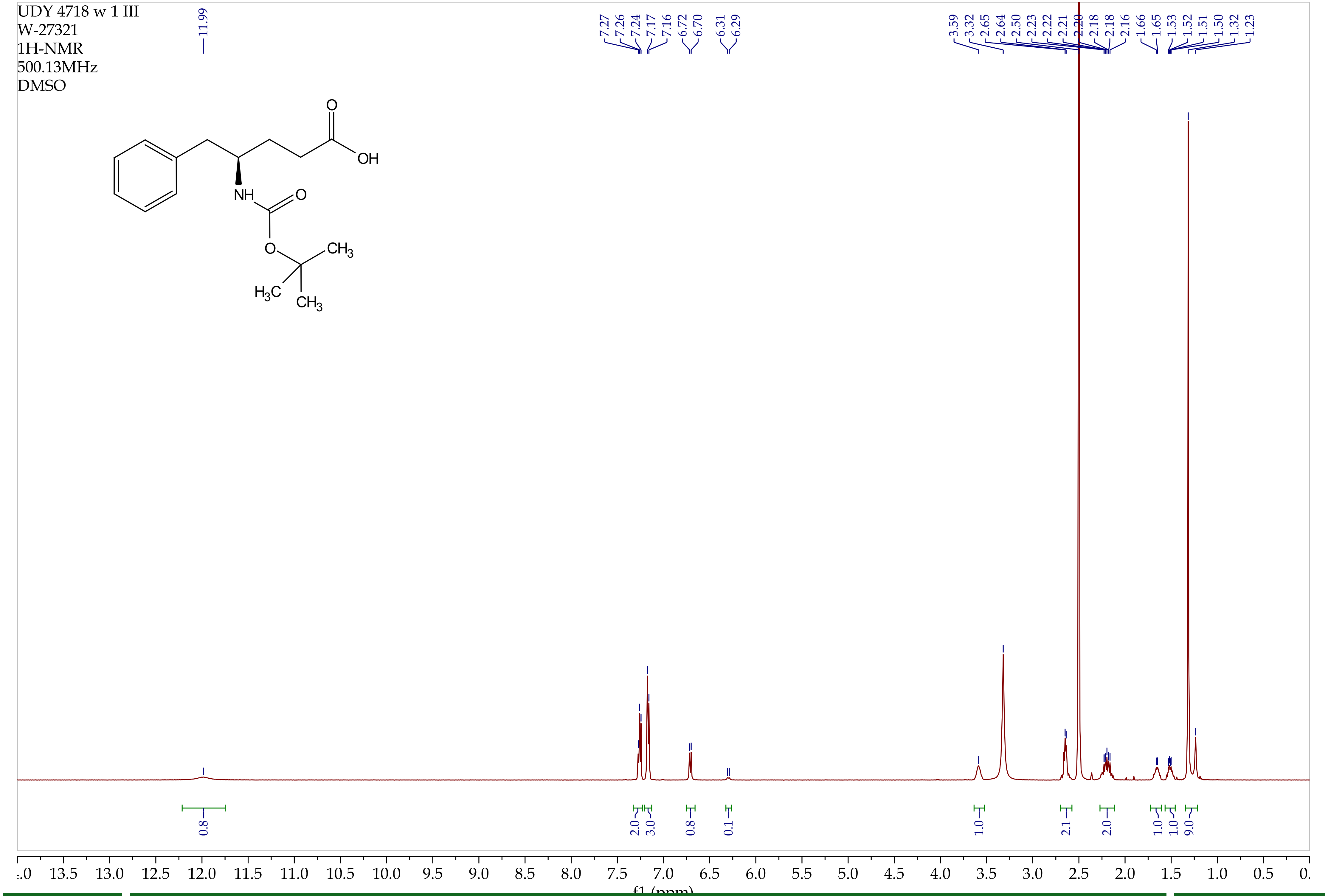
UDY 4718 w 1 III
W-27321
1H-NMR
500.13MHz
DMSO
O
OH
NH
O
O
CH3
H3C
CH3
11.99
7.27
7.26
7.24
7.17
7.16
6.72
6.70
6.31
6.29
3.59
3.32
2.65
2.64
2.50
2.23
2.22
2.21
2.20
2.18
2.18
2.16
1.66
1.65
1.53
1.52
1.51
1.50
1.32
1.23
0.8
2.0
3.0
0.8
0.1
1.0
2.1
2.0
1.0
1.0
9.0
13.5
13.0
12.5
12.0
11.5
11.0
10.5
10.0
9.5
9.0
8.5
8.0
7.5
7.0
6.5
6.0
5.5
5.0
4.5
4.0
3.5
3.0
2.5
2.0
1.5
1.0
0.5
f1 (ppm)

ChiroBlock® GmbH, Andresenstraße 1a, 06766 Wolfen
Tel.: ++49 / 3494 / 63 83 23 Fax: ++49 / 3494 / 63 83 24

Internal Analysis for QC
MS

Sample Information

UDY 4718 w1 III

Shimadzu CLASS-VP V6.12 SP5 Area % Report

Acquisition Date:	01.04.2019
Method Name:	Iso 20%A, 210nm, 0.2ml, M50-2000, -10V + 10V.lcm
Data Name:	Z:\Labor\LC\LC-An\LC-An-HPL-02\LC-MS\LC-MS-Läufe\15151-16000LC+LCMS\15151.lcd
Column:	direct injection
Eluents:	A) H2O B) acetonitrile
Flow rate:	0.2 ml/min
Detection:	UV, 210 nm
Probe voltage:	+4,5 kV (ESI-positive mode)

MS Chromatogram
Z:\Labor\LC\LC-An\LC-An-HPL-02\LC-MS\LC-MS-Läufe\15151-16000LC+LCMS\15151.lcd

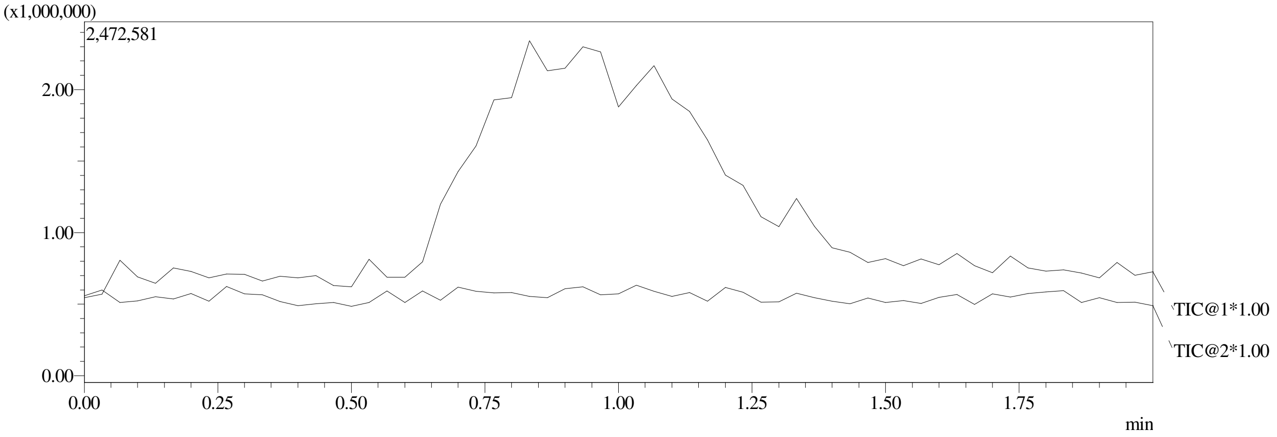

MS Spectrum Graph

#:1 Ret.Time:Averaged 0.667-1.333(Scan#:41-81)
BG Mode:Averaged 0.000-0.529(1-33)
Mass Peaks:1225 Base Peak:315.90(398480) Polarity:Pos Segment1 - Event1

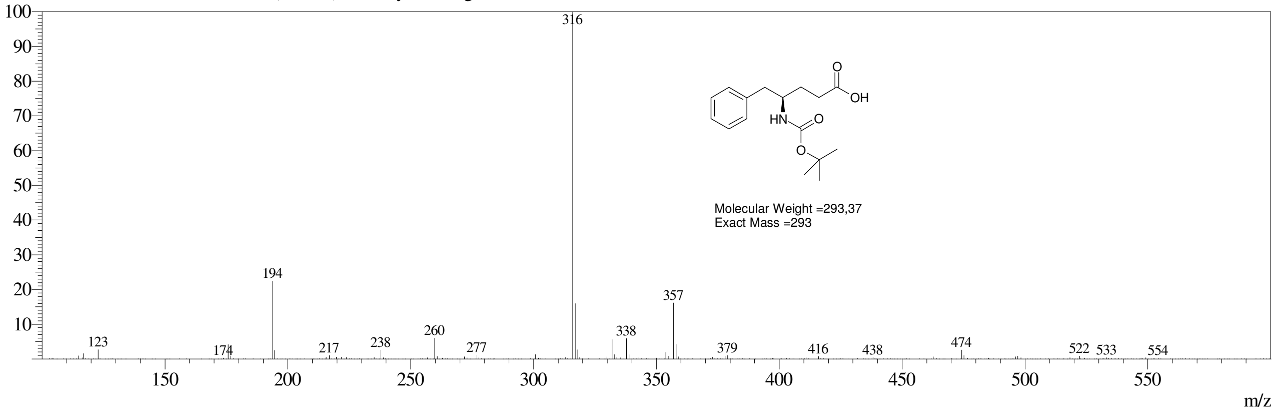

Supplement: Supplementary file 1 — Supplementary file1 See the certificate of analysis for BGPHEOH for 1H-NMR and QC-MS spectra of BGPHEOH, as supplied by ChiroBlock GmbH. The crystal data and structure refinement parameters, conformational analysis of the molecular structures, intermolecular hydrogen bond parameters, packing of molecules in single crystals, and NBO calculation results are provided in the supplementary file (PDF 328 KB) [file 726_2023_3372_MOESM1_ESM.pdf]
