# Supplementary material for: Investigation of encapsulated water wire within self-assembled hydrophilic nanochannels, in a modified γ4-amino acid crystals: Tracking thermally induced changes of intermolecular interactions within a crystalline hydrate
Source: Amino Acids. 2024 Feb 5;56(1):9. doi: 10.1007/s00726-023-03372-4 (PMC10844418; doi:10.1007/s00726-023-03372-4)
Supplement: Supplementary file 2 — Supplementary file2 (DOCX 2682 KB) [file 726_2023_3372_MOESM2_ESM.docx]

**Table S1.** Crystal data and structure refinement parameters for the datasets collected with **crystal 1**, X-ray focused on position 1.

| Compounds | **BGPHEOH1_80K** |
| --- | --- |
| Empirical formula | C16 H24 N1 O4.5 |
| Formula weight | 302.36 |
| Crystal color/habit | Colorless/ Needle |
| Crystal size (mm) | (0.310 × 0.016 × 0.013) |
| Crystallizing solvent | Methanol/Water |
| Crystal system/Space group | Monoclinic / *P*2_1_ |
| *a* (Å) | 13.9700(2) |
| *b* (Å) | 5.2000(3) |
| *c* (Å) | 22.2600(3) |
| *β* (°) | 96.9500(10) |
| Volume (Å^3^) | 1605.17(10) |
| CCDC No. | **2239361** |
| Z/Z′ | 4/2 |
| Calculated density (g/cm^3^) | 1.251 |
| F (000) | 652 |
| Radiation | Synchrotron (λ = 0.61990 Å) |
| Temperature (K) | 80(2) |
| θ range (˚) | 0.804-26.926 |
| Scan type | φ |
| Measured reflections | 31147 |
| Unique reflections | 10189 |
| Observed reflection [│F│ > 4σ(F)] | 9439 |
| Final R (%) | 6.42 |
| *w*R2 (%) | 17.09 |
| Goodness-of-fit on F^2^ (S) | 1.079 |
| Δρ max (e.Å^-3^) | 0.750 |
| Δρ min(e.Å^-3^) | -0.959 |
| No. of restraints/parameters | 6/410 |
| Data[│F│ > 4σ(F)]-to-parameter ratio | 23.02: 1 |

**Table S2.** Crystal data and structure refinement parameters for the datasets collected with **crystal 2**.

| Compounds | **BGPHEOH2_RT** | **BGPHEOH2_305K** | **BGPHEOH2_315K** | **BGPHEOH2_325K** |
| --- | --- | --- | --- | --- |
| Empirical formula | C16 H24 N1 O4.5 | C16 H24 N1 O4.5 | C16 H24 N1 O4.5 | C16 H24 N1 O4.5 |
| Formula weight | 302.36 | 302.36 | 302.36 | 302.36 |
| Crystal color/habit | Colorless/ Needle | | | |
| Crystal size (mm) | (0.220 × 0.013 × 0.010) | | | |
| Crystallizing solvent | Methanol/Water | | | |
| Crystal system/Space group | Monoclinic / *P*2_1_ | | | |
| *a* (Å) | 14.0700(2) | 14.0700(2) | 14.0600(2) | 14.0400(2) |
| *b* (Å) | 5.2600(3) | 5.2600(3) | 5.2700(3) | 5.2700(3) |
| *c* (Å) | 22.5900(3) | 22.6400(3) | 22.6900(3) | 22.7800(3) |
| *β* (°) | 97.2400(10) | 97.2600(10) | 97.3000(10) | 97.3600(10) |
| Volume (Å^3^) | 1658.52(10) | 1662.11(10) | 1667.62(10) | 1671.62(10) |
| CCDC No. | **2231852** | **2231853** | **2231854** | **2231855** |
| Z/Z′ | 4/2 | | | |
| Calculated density (g/cm^3^) | 1.207 | 1.204 | 1.204 | 1.201 |
| F (000) | 648 | 648 | 652 | 652 |
| Radiation | Synchrotron (λ = 0.61990 Å) | | | |
| Temperature (K) | 296(2) | 305(2) | 315(2) | 325(2) |
| θ range (˚) | 0.792-27.001 | 0.791-27.015 | 0.789-26.997 | 1.275-27.008 |
| Scan type | φ | | | |
| Measured reflections | 34360 | 34732 | 34928 | 34830 |
| Unique reflections | 10770 | 10851 | 10898 | 10916 |
| Observed reflection [│F│ > 4σ(F)] | 5224 | 5057 | 4880 | 4539 |
| Final R (%) | 10.09 | 10.21 | 9.67 | 10.25 |
| *w*R2 (%) | 25.97 | 26.30 | 24.79 | 26.54 |
| Goodness-of-fit on F^2^ (S) | 0.877 | 0.927 | 0.918 | 0.909 |
| Δρ max (e.Å^-3^) | 0.383 | 0.454 | 0.372 | 0.559 |
| Δρ min(e.Å^-3^) | -0.337 | -0.325 | -0.302 | -0.313 |
| No. of restraints/parameters | 4/414 | 21/449 | 31/460 | 28/449 |
| Data[│F│ > 4σ(F)]-to-parameter ratio | 12.62: 1 | 11.26:1 | 10.61: 1 | 10.11:1 |

**Table S3.** Crystal data and structure refinement parameters for the datasets collected with **crystal 2**.

| Compounds | **BGPHEOH2_335K** | **BGPHEOH2_345K** |
| --- | --- | --- |
| Empirical formula | C16 H24 N1 O4.5 | C16 H23 N1 O4.5 |
| Formula weight | 301.35 | 301.35 |
| Crystal color/habit | Colorless/ Needle | |
| Crystal size (mm) | (0.220 × 0.013 × 0.010) | |
| Crystallizing solvent | Methanol/Water | |
| Crystal system/Space group | Monoclinic / *P*2_1_ | Triclinic / *P*1 |
| *a* (Å) | 14.0200(2) | 5.2600(2) |
| *b* (Å) | 5.2800(3) | 13.9400(3) |
| *c* (Å) | 22.8700(3) | 22.8800(3) |
| *α* (°) |  | 97.5500(10) |
| *β* (°) | 97.4300(10) | 89.9400(10) |
| *γ*(°) |  | 89.8000(10) |
| Volume (Å^3^) | 1678.75(10) | 1663.11(10) |
| CCDC No. | **2231856** |  |
| Z/Z′ | 4/2 | 4/4 |
| Calculated density (g/cm^3^) | 1.192 |  |
| F (000) | 648 |  |
| Radiation | Synchrotron (λ = 0.61990 Å) | |
| Temperature (K) | 335(2) | 345(2) |
| θ range (˚) | 0.783-27.008 |  |
| Scan type | φ | |
| Measured reflections | 35315 |  |
| Unique reflections | 10972 |  |
| Observed reflection [│F│ > 4σ(F)] | 4275 |  |
| Final R (%) | 10.36 |  |
| *w*R2 (%) | 27.98 |  |
| Goodness-of-fit on F^2^ (S) | 0.934 |  |
| Δρ max (e.Å^-3^) | 0.710 |  |
| Δρ min(e.Å^-3^) | -0.337 |  |
| No. of restraints/parameters | 10/399 |  |
| Data[│F│ > 4σ(F)]-to-parameter ratio | 10.71: 1 |  |

**Table S4.** Crystal data and structure refinement parameters for the datasets collected with **crystal 3**, X-ray focused on position 1.

| Compounds | **BGPHEOH3_335K** | **BGPHEOH3_337K** |
| --- | --- | --- |
| Empirical formula | C16 H24 N1 O4.5 | C16 H23 N1 O4.5 |
| Formula weight | 301.35 | 301.35 |
| Crystal color/habit | Colorless/ Needle | |
| Crystal size (mm) | (0.350 × 0.014 × 0.012) | |
| Crystallizing solvent | Methanol/Water | |
| Crystal system/Space group | Monoclinic / *P*2_1_ | |
| *a* (Å) | 14.0200(2) | 14.0400(2) |
| *b* (Å) | 5.2800(3) | 5.2900(3) |
| *c* (Å) | 22.8400(3) | 22.9000(3) |
| *β* (°) | 97.4200(10) | 97.3900(10) |
| Volume (Å^3^) | 1676.59(10) | 1686.69(10) |
| CCDC No. | **2231857** | **2231858** |
| Z/Z′ | 4/2 | 4/2 |
| Calculated density (g/cm^3^) | 1.194 | 1.187 |
| F (000) | 648 | 648 |
| Radiation | Synchrotron (λ = 0.61990 Å) | |
| Temperature (K) | 335(2) | 337(2) |
| θ range (˚) | 1.277-27.010 | 1.276-27.059 |
| Scan type | φ | |
| Measured reflections | 35493 | 33294 |
| Unique reflections | 10236 | 10341 |
| Observed reflection [│F│ > 4σ(F)] | 4646 | 2936 |
| Final R (%) | 7.29 | 10.67 |
| *w*R2 (%) | 19.10 | 29.04 |
| Goodness-of-fit on F^2^ (S) | 0.999 | 0.975 |
| Δρ max (e.Å^-3^) | 0.656 | 0.698 |
| Δρ min(e.Å^-3^) | -0.337 | -0.395 |
| No. of restraints/parameters | 28/453 | 13/391 |
| Data[│F│ > 4σ(F)]-to-parameter ratio | 10.26: 1 | 7.51: 1 |

**Table S5.** Crystal data and structure refinement parameters for the datasets collected with **crystal 3**, X-ray focused on position 2.

| Compounds | **BGPHEOH3_337K_2** | **BGPHEOH3_339K_2** | **BGPHEOH3_340K_2** | **BGPHEOH3_342K_2** |
| --- | --- | --- | --- | --- |
| Empirical formula | C16 H24 N1 O4.5 | C16 H24 N1 O4.5 | C16 H24 N1 O4.5 | C16 H24 N1 O4.5 |
| Formula weight | 302.36 | 302.36 | 302.36 | 302.36 |
| Crystal color/habit | Colorless/ Needle | | | |
| Crystal size (mm) | (0.350 × 0.014 × 0.012) | | | |
| Crystallizing solvent | Methanol/Water | | | |
| Crystal system/Space group | Monoclinic / *P*2_1_ | | | Triclinic / *P*1 |
| *a* (Å) | 14.0100(2) | 14.0000(2) | 13.9900(2) | 5.3000(2) |
| *b* (Å) | 5.2800(3) | 5.2800(3) | 5.2800(3) | 14.0300(3) |
| *c* (Å) | 22.8900(3) | 22.9300(3) | 22.9400(3) | 22.9700(3) |
| *α* (°) |  |  |  | 97.5500(10) |
| *β* (°) | 97.4500(10) | 97.4300(10) | 97.4400(10) | 90.0500(10) |
| *γ*(°) |  |  |  | 90.0100(10) |
| Volume (Å^3^) | 1678.94(10) | 1680.75(10) | 1680.25(10) | 1693.22(10) |
| CCDC No. | **2231859** | **2231860** | **2231861** |  |
| Calculated density (g/cm^3^) | 1.192 | 1.191 | 1.191 |  |
| F (000) | 648 | 648 | 648 |  |
| Radiation | Synchrotron (λ = 0.61990 Å) | | | |
| Temperature (K) | 337(2) | 339(2) | 340(2) | 342(2) |
| θ range (˚) | 1.278-27.018 | 0.781-27.017 | 1.280-27.108 |  |
| Scan type | φ | | | |
| Measured reflections | 35072 | 34847 | 34376 |  |
| Unique reflections | 10742 | 10751 | 10747 |  |
| Observed reflection [│F│ > 4σ(F)] | 5252 | 4770 | 4236 |  |
| Final R (%) | 7.65 | 7.84 | 8.65 |  |
| *w*R2 (%) | 21.60 | 20.59 | 23.67 |  |
| Goodness-of-fit on F^2^ (S) | 1.027 | 1.117 | 0.981 |  |
| Δρ max (e.Å^-3^) | 0.891 | 0.905 | 0.801 |  |
| Δρ min(e.Å^-3^) | -0.384 | -0.411 | -0.251 |  |
| No. of restraints/parameters | 10/405 | 33/449 | 41/437 |  |
| Data[│F│ > 4σ(F)]-to-parameter ratio | 12.97: 1 | 10.62:1 | 9.52: 1 |  |

**Table S6.** Hydrogen bond parameters in the crystal structures for the dataset were collected at **80K** with **crystal 1**.

| Donor | Acceptor | D⋅⋅⋅A (Å) | H⋅⋅⋅A (Å) | D−H⋅⋅⋅A (°) |
| --- | --- | --- | --- | --- |
| **BGPHEOH1_80K** | | | | |
| N1-H1 | O01 [ x, y+1, z] | 3.049 | 2.277 | 149.55 |
| N1'-H1' | O01' [ x, y+1, z] | 3.037 | 2.203 | 163.41 |
| **O1W** | **O1W [ -x, y+1/2, -z]** | **3.127** | **2.470** | **124.52** |
| O2' | O1W | 2.594 | 1.626 | 166.48 |
| O2 | O1' [ x+1, y-1, z] | 2.684 | 1.761 | 158.14 |
| O1W | O1' [ -x-1, y-1/2, -z+1] | 2.879 | 2.345 | 113.74 |

**Table S7.** Hydrogen bond parameters in the crystal structures for the datasets collected with **crystal 2**.

| Donor | Acceptor | D⋅⋅⋅A (Å) | H⋅⋅⋅A (Å) | D−H⋅⋅⋅A (°) |
| --- | --- | --- | --- | --- |
| **BGPHEOH2_296K** | | | | |
| N1 | O01 [ x, y-1, z] | 3.093 | 2.329 | 162.36 |
| N1' | O0'' [ x, y-1, z] | 3.101 | 2.257 | 165.38 |
| **O1W** | **O1W [ -x, y+1/2, -z]** | **3.229** | **2.424** | **140.42** |
| O1W | O1' | 2.816 | 1.999 | 141.53 |
| O2 | O1W [ -x+1, y-1/2, -z] | 2.611 | 1.806 | 166.54 |
| O2' | O1 [ x-1, y+1, z] | 2.694 | 1.886 | 168.37 |
| **BGPHEOH2_305K** | | | | |
| N1 | O01 [ x, y-1, z] | 3.104 | 2.286 | 159.59 |
| N1' | O01' [ x, y-1, z] | 3.107 | 2.262 | 167.37 |
| O2 | O1W [ -x, y+1/2, -z] | 2.619 | 1.751 | 145.99 |
| **O1W** | **O1W [ -x, y+1/2, -z]** | **3.232** | **2.598** | **123.98** |
| O1W | O1 | 2.936 | 2.574 | 102.21 |
| O2' | O1 | 2.698 | 1.891 | 167.88 |
| **BGPHEOH2_315K** | | | | |
| **O1W** | **O1W [ -x, y+1/2, -z]** | **3.238** | **2.720** | **114.26** |
| O1W | O1 [ -x, y+1/2, -z] | 2.937 | 2.560 | 103.25 |
| N1' | O01' [ x, y-1, z] | 3.117 | 2.263 | 174.66 |
| O2' | O1 [ x-1, y+1, z] | 2.708 | 1.733 | 174.51 |
| N1 | O01 [ x, y-1, z] | 3.100 | 2.278 | 160.82 |
| O2 | O1W | 2.626 | 1.838 | 136.38 |
| **BGPHEOH2_325K** | | | | |
| N1' | O0'' [ x, y-1, z] | 3.120 | 2.342 | 150.01 |
| N1 | O01 [ x, y-1, z] | 3.092 | 2.297 | 154.57 |
| O2 | O1W [ -x, y+1/2, -z] | 2.634 | 2.010 | 120.09 |
| O2' | O1 | 2.711 | 1.925 | 159.99 |
| **O1W** | **O1W [ -x, y+1/2, -z]** | **3.208** | **2.316** | **153.61** |
| O1W | O1 | 2.954 | 2.500 | 108.78 |
| O1W | O1' | 2.824 | 2.126 | 129.10 |
| **BGPHEOH2_335K** | | | | |
| N1' | O0" [ x, y-1, z] | 3.129 | 2.345 | 151.76 |
| O2' | O1 [ x+1, y+1, z] | 2.724 | 1.949 | 157.17 |
| N1 | O01 [ x, y-1, z] | 3.099 | 2.275 | 160.30 |
| O2 | O1W | 2.628 | 1.815 | 171.69 |
| **O1W** | **O1W [-x, y+1/2, -z+2]** | **3.204** | **2.481** | **132.02** |
| O1W | O1 [-x, y+1/2, -z] | 2.975 | 2.180 | 139.36 |
| O1W | O1' [-x+1, y-1/2, -z] | 2.828 | 1.913 | 160.31 |

**Table S8.** Hydrogen bond parameters in the crystal structures for the datasets collected with **crystal 3**.

| Donor | Acceptor | | D⋅⋅⋅A (Å) | H⋅⋅⋅A (Å) | D−H⋅⋅⋅A (°) |
| --- | --- | --- | --- | --- | --- |
| **Crystal 3** position 1 | | | | | |
| **BGPHEOH3_335K** | | | | | |
| N1 | O01 [ x, y-1, z] | | 3.114 | 2.300 | 160.40 |
| N1' | O0'' [ x, y-1, z] | | 3.135 | 2.287 | 167.59 |
| **O1W** | **O1W [ -x, y+1/2, -z]** | | **3.186** | **2.501** | **126.81** |
| O1W | O1 [-x, y+1/2, -z] | | 2.951 | 2.608 | 100.68 |
| O1W | O1' [-x, y+1/2, -z] | | 2.825 | 1.947 | 149.46 |
| O2 | O1W | | 2.641 | 1.798 | 142.61 |
| O2' | O1 | | 2.731 | 1.792 | 159.56 |
| **BGPHEOH3_337K** | | | | | |
| N1 | O01 [ x, y-1, z] | | 3.120 | 2.301 | 159.14 |
| N1' | O01' [ x, y-1, z] | | 3.147 | 2.370 | 150.43 |
| O2 | O1W | | 2.585 | 1.776 | 168.80 |
| **O1W** | **O1W [ -x, y+1/2, -z+2]** | | **3.217** |  |  |
| O1W | O1 [-x, y+1/2, -z+2] | | 2.988 |  |  |
| O1W | O1' [-x, y+1/2, -z+2] | | 2.853 |  |  |
| O2' | O1 | | 2.742 | 2.172 | 126.69 |
| **Crystal 3** position 2 | | | | | |
| **BGPHEOH3_337K_2** | | | | | |
| N1 | | O01 [ x, y-1, z] | 3.102 | 2.283 | 159.06 |
| N1' | | O0'' [ x, y-1, z] | 3.131 | 2.348 | 151.54 |
| **O1W** | | **O1W [-x, y+1/2, -z]** | **3.198** | **2.515** | **127.53** |
| O1W | | O1 | 2.985 | 2.192 | 138.33 |
| O1W | | O1' | 2.826 | 1.908 | 165.32 |
| O2' | | O1 | 2.748 | 1.997 | 151.93 |
| O2 | | O1W [ -x, y+1/2, -z] | 2.620 | 1.812 | 168.28 |
| **BGPHEOH3_339K_2** | | | | | |
| N1 | | O01 [ x, y-1, z] | 3.099 | 2.311 | 154.07 |
| N1' | | O0'' [ x, y-1, z] | 3.134 | 2.354 | 151.05 |
| O2 | | O1W | 2.625 | 1.718 | 151.27 |
| **O1W** | | **O1W [-x, y+1/2, -z]** | **3.185** | **2.656** | **114.51** |
| O1W | | O1 [-x, y+1/2, -z] | 2.974 | 2.072 | 153.48 |
| O1W | | O1' [-x, y+1/2, -z] | 2.819 | 1.901 | 161.56 |
| O2' | | O1 | 2.736 | 1.912 | 139.78 |
| **BGPHEOH3_340K_2** | | | | | |
| N1 | | O01 [ x, y-1, z] | 3.103 | 2.337 | 149.00 |
| N1' | | O0'' [ x, y-1, z] | 3.128 | 2.261 | 172.07 |
| O2 | | O1W [-x, y+1/2, -z] | 2.610 | 1.648 | 165.66 |
| **O1W** | | **O1W [-x, y+1/2, -z]** | **3.183** | **2.723** | **109.80** |
| O1W | | O1 | 2.984 | 2.129 | 146.74 |
| O1W | | O1' | 2.824 | 1.901 | 159.22 |
| O2' | | O1 | 2.754 | 1.879 | 146.98 |

**Figure S1**. A particular frame from single crystals X-ray diffraction measurements with **crystal 2** collected at different temperatures are exhibited.

**Figure S2**. A particular frame from single crystals X-ray diffraction measurements with **crystal 3** collected at different temperatures are exhibited.

**Figure S3. The water molecules involved in the hydrogen bond interactions as a donor. a) At 325K water molecules form 2 hydrogen bonds as donor. b) At 335K water molecules form 3 hydrogen bonds as donor.**

**Figure S4.** Total interaction energy along the crystallographic ‘*b*’ axis.

**Table S9.** The intermolecular interaction energy of the interactions involving a water molecule present in the water chain. NBO calculation with BGPHEOH structures obtained at different temperatures from single crystal XRD. Second Order Perturbation Theory Analysis of Fock Matrix in NBO Basis. Threshold for printing: E(2)>0.50 kcal/mol.

| **Donor NBO’s (i)** | **Acceptor NBO’s (j)** | **E(2) (kcal/mol)** | **E(j) – E(i)**  **(a.u.)** | | **F(i, j)**  **(a.u.)** |
| --- | --- | --- | --- | --- | --- |
| **296K (Crystal 2)** | | | | | |
| from unit 1 to unit 2 | | | | | |
| 868. LP ( 1) O 91 | /***. RY*( 3) H 92 | 0.21 | 2.27 | | 0.02 |
| 868. LP ( 1) O 91 | /***. RY*( 1) O 94 | 0.06 | 1.91 | | 0.01 |
| 868. LP ( 1) O 91 | /***. BD*( 1) H 92 - O 94 | 0.79 | 1.18 | | 0.03 |
| **total interaction between unit 1 and 2** | | **1.06** | **5.36** | | **0.06** |
| from unit 3 to unit 1 | | | | | |
| 881. LP ( 1) O 141 | /***. RY*( 3) H 89 | 0.12 | 2.38 | | 0.02 |
| 881. LP ( 1) O 141 | /***. BD*( 1) H 89 - O 91 | 0.77 | 1.18 | | 0.03 |
| **total interaction between unit 1 and 3** | | **0.89** | **3.56** | | **0.04** |
| from unit 1 to unit 4 | | | | | |
| 99. BD ( 1) H 89 - O 91 | /***. RY*( 4) O 240 | 0.19 | 2.14 | | 0.02 |
| 99. BD ( 1) H 89 - O 91 | /***. RY*( 1) H 241 | 0.26 | 1.91 | | 0.02 |
| 99. BD ( 1) H 89 - O 91 | /***. RY*( 2) H 241 | 0.06 | 2.61 | | 0.01 |
| 99. BD ( 1) H 89 - O 91 | /***. RY*( 4) H 241 | 0.09 | 2.86 | | 0.01 |
| 99. BD ( 1) H 89 - O 91 | /***. BD*( 1) O 240 - H 241 | 0.67 | 1.72 | | 0.03 |
| 100. BD ( 1) H 90 - O 91 | /***. RY*( 1) H 241 | 0.07 | 1.93 | | 0.01 |
| 868. LP ( 1) O 91 | /***. RY*( 4) H 241 | 0.07 | 2.69 | | 0.01 |
| 868. LP ( 1) O 91 | /***. BD*( 1) O 240 - H 241 | 0.61 | 1.55 | | 0.03 |
| 869. LP ( 2) O 91 | /***. RY*( 4) O 240 | 0.56 | 1.80 | | 0.03 |
| 869. LP ( 2) O 91 | /***. RY*( 9) O 240 | 0.06 | 4.07 | | 0.01 |
| 869. LP ( 2) O 91 | /***. RY*( 1) H 241 | 0.16 | 1.56 | | 0.01 |
| 869. LP ( 2) O 91 | /***. RY*( 3) H 241 | 0.09 | 2.19 | | 0.01 |
| 869. LP ( 2) O 91 | /***. RY*( 4) H 241 | 0.17 | 2.51 | | 0.02 |
| 869. LP ( 2) O 91 | /***. BD*( 1) O 240 - H 241 | 13.05 | 1.37 | | 0.12 |
| 869. LP ( 2) O 91 | /***. BD*( 1) O 240 - C 248 | 0.42 | 0.94 | | 0.02 |
| **total interaction between unit 1 and 4** | | **16.53** | **31.85** | | **0.37** |
| from unit 1 to unit 5 | | | | | |
| 100. BD ( 1) H 90 - O 91 | /***. BD*( 1) C 15 - O 22 | 0.25 | 1.54 | | 0.02 |
| from unit 5 to unit 1 | | | | | |
| 19. BD ( 1) C 12 - C 15 | /***. BD*( 1) H 90 - O 91 | 0.15 | 1.27 | | 0.01 |
| 857. LP ( 1) O 22 | /***. BD*( 1) H 90 - O 91 | 2.25 | 1.34 | | 0.05 |
| 858. LP ( 2) O 22 | /***. BD*( 1) H 90 - O 91 | 2.35 | 0.90 | | 0.04 |
| **total interaction between unit 1 and 5** | | **5.00** | **5.05** | | **0.12** |
| from unit 1 to unit 6 | | | | | |
| 869. LP ( 2) O 91 | /***. BD*( 1) C 336 - O 340 | 0.20 | 1.17 | | 0.01 |
| from unit 6 to unit 1 | | | | | |
| 927. LP ( 2) O 340 | /***. BD*( 1) H 89 - O 91 | 0.06 | 0.88 | | 0.01 |
| total interaction between unit 1 and 6 | | 0.26 | 2.05 | | 0.02 |
| from unit 1 to unit 7 | | | | | |
| 869. LP ( 2) O 91 | /***. RY*( 1) H 80 | 0.06 | 1.23 | | 0.01 |
| from unit 1 to unit 8 | | | | | |
| 868. LP ( 1) O 91 | /***. RY*( 1) H 493 | 0.05 | 1.51 | | 0.01 |
| **305K (Crystal 2)** | | | | | |
| from unit 2 to unit 1 | | | | | |
| 99. BD ( 1) H 89 - O 91 | /***. RY*( 2) H 93 | 0.07 | 2.63 | | 0.01 |
| 99. BD ( 1) H 89 - O 91 | /***. BD*( 1) H 93 - O 94 | 0.28 | 1.30 | | 0.02 |
| 868. LP ( 1) O 91 | /***. RY*( 1) H 93 | 0.40 | 1.31 | | 0.02 |
| 868. LP ( 1) O 91 | /***. RY*( 1) O 94 | 0.07 | 1.65 | | 0.01 |
| 868. LP ( 1) O 91 | /***. BD*( 1) H 93 - O 94 | 0.27 | 1.13 | | 0.02 |
| from unit 1 to unit 2 | | | | | |
| 101. BD ( 1) H 92 - O 94 | /***. BD*( 1) H 89 - O 91 | 0.10 | 1.37 | | 0.01 |
| 102. BD ( 1) H 93 - O 94 | /***. BD*( 1) H 89 - O 91 | 0.24 | 1.36 | | 0.02 |
| 870. LP ( 1) O 94 | /***. BD*( 1) H 89 - O 91 | 0.09 | 1.20 | | 0.01 |
| **total interaction between 1 and 2** | | **1.52** | **11.95** | | **0.11** |
| from unit 1 to unit 3 | | | | | |
| 101. BD ( 1) H 92 - O 94 | /***. RY*( 3) H 96 | 0.08 | 2.58 | | 0.01 |
| 101. BD ( 1) H 92 - O 94 | /***. BD*( 1) H 96 - O 97 | 0.26 | 1.30 | | 0.02 |
| 870. LP ( 1) O 94 | /***. RY*( 1) H 96 | 0.07 | 1.19 | | 0.01 |
| 870. LP ( 1) O 94 | /***. BD*( 1) H 96 - O 97 | 0.27 | 1.13 | | 0.02 |
| from unit 3 to unit 1 | | | | | |
| 103. BD ( 1) H 95 - O 97 | /***. BD*( 1) H 92 - O 94 | 0.12 | 1.36 | | 0.01 |
| 104. BD ( 1) H 96 - O 97 | /***. BD*( 1) H 92 - O 94 | 0.27 | 1.35 | | 0.02 |
| 872. LP ( 1) O 97 | /***. BD*( 1) H 92 - O 94 | 0.09 | 1.17 | | 0.01 |
| **total interaction between 1 and 3** | | **1.16** | **10.08** | | **0.09** |
| from unit 1 to unit 4 | | | | | |
| 101. BD ( 1) H 92 - O 94 | /994. RY*( 1) H 7 | 0.10 | 1.71 | | 0.01 |
| 101. BD ( 1) H 92 - O 94 | /***. RY*( 4) O 15 | 0.25 | 2.24 | | 0.02 |
| 101. BD ( 1) H 92 - O 94 | /***. BD*( 1) H 7 - O 15 | 2.21 | 1.33 | | 0.05 |
| 102. BD ( 1) H 93 - O 94 | /995. RY*( 2) H 7 | 0.09 | 2.87 | | 0.01 |
| 638. CR ( 1) O 94 | /***. BD*( 1) H 7 - O 15 | 0.14 | 19.90 | | 0.05 |
| 870. LP ( 1) O 94 | /995. RY*( 2) H 7 | 0.19 | 2.71 | | 0.02 |
| 870. LP ( 1) O 94 | /997. RY*( 4) H 7 | 0.05 | 2.61 | | 0.01 |
| 870. LP ( 1) O 94 | /***. BD*( 1) H 7 - O 15 | 0.95 | 1.16 | | 0.03 |
| 871. LP ( 2) O 94 | /996. RY*( 3) H 7 | 0.07 | 2.32 | | 0.01 |
| 871. LP ( 2) O 94 | /***. RY*( 4) O 15 | 0.14 | 1.90 | | 0.02 |
| 871. LP ( 2) O 94 | /***. BD*( 1) H 7 - O 15 | 16.56 | 0.99 | | 0.11 |
| 871. LP ( 2) O 94 | /***. BD*( 1) O 15 - C 19 | 0.11 | 1.02 | | 0.01 |
| 871. LP ( 2) O 94 | /***. BD*( 1) C 19 - C 35 | 0.08 | 0.97 | | 0.01 |
| 871. LP ( 2) O 94 | /***. BD*( 1) C 19 - O 36 | 0.06 | 1.26 | | 0.01 |
| from unit 4 to unit 1 | | | | | |
| 7. BD ( 1) H 7 - O 15 | /***. BD*( 1) H 93 - O 94 | 0.07 | 1.38 | | 0.01 |
| **total interaction between 1 and 4** | | **21.07** | **44.37** | | **0.38** |
| **from unit 1 to unit 5** | | | | | |
| **870. LP ( 1) O 94** | **/***. BD*( 1) C 109 - O 119** | **0.28** | **1.42** | | **0.02** |
| from unit 1 to unit 6 | | | | | |
| 102. BD ( 1) H 93 - O 94 | /***. BD*( 1) C 204 - O 221 | 0.06 | 1.59 | | 0.01 |
| 871. LP ( 2) O 94 | /***. BD*( 1) C 204 - O 221 | 0.16 | 1.26 | | 0.01 |
| from unit 6 to unit 1 | | | | | |
| 228. BD ( 2) C 204 - O 221 | /***. BD*( 1) H 93 - O 94 | 0.05 | 0.96 | | 0.01 |
| 900. LP ( 2) O 221 | /***. BD*( 1) H 92 - O 94 | 0.07 | 0.84 | | 0.01 |
| 900. LP ( 2) O 221 | /***. BD*( 1) H 93 - O 94 | 0.06 | 0.83 | | 0.01 |
| **total interaction between 1 and 6** | | **0.40** | **5.48** | | **0.04** |
| **315K (Crystal 2)** | | | | | |
| from unit 2 to unit 1 | | | | | |
| 1. BD ( 1) O 1 - H 2 | /***. RY*( 1) H 5 | 0.09 | 1.60 | | 0.01 |
| 1. BD ( 1) O 1 - H 2 | /***. RY*( 4) H 6 | 0.07 | 3.09 | | 0.01 |
| 1. BD ( 1) O 1 - H 2 | /***. BD*( 1) O 4 - H 6 | 0.07 | 1.33 | | 0.01 |
| 850. LP ( 1) O 1 | /***. RY*( 4) H 5 | 0.05 | 2.97 | | 0.01 |
| from unit 1 to unit 2 | | | | | |
| 4. BD ( 1) O 4 - H 6 | /979. RY*( 3) H 2 | 0.06 | 2.58 | | 0.01 |
| 852. LP ( 1) O 4 | /977. RY*( 1) H 2 | 0.11 | 1.38 | | 0.01 |
| **total interaction between unit 1 and 2** | | **0.45** | **12.95** | | **0.07** |
| from unit 1 to unit 3 | | | | | |
| 3. BD ( 1) O 4 - H 5 | /***. RY*( 1) H 316 | 0.05 | 1.71 | | 0.01 |
| 3. BD ( 1) O 4 - H 5 | /***. RY*( 4) H 317 | 0.07 | 3.15 | | 0.01 |
| 3. BD ( 1) O 4 - H 5 | /***. BD*( 1) O 315 - H 317 | 0.06 | 1.35 | | 0.01 |
| 852. LP ( 1) O 4 | /***. RY*( 4) H 316 | 0.05 | 2.97 | | 0.01 |
| from unit 3 to unit 1 | | | | | |
| 349. BD ( 1) O 315 - H 317 | /***. RY*( 3) H 5 | 0.06 | 2.62 | | 0.01 |
| 349. BD ( 1) O 315 - H 317 | /***. BD*( 1) O 4 - H 5 | 0.05 | 1.30 | | 0.01 |
| 917. LP ( 1) O 315 | /987. RY*( 1) O 4 | 0.06 | 1.73 | | 0.01 |
| 917. LP ( 1) O 315 | /***. RY*( 1) H 5 | 0.16 | 1.48 | | 0.01 |
| **total interaction between unit 1 and 3** | | **0.56** | **16.31** | | **0.08** |
| from unit 1 to unit 4 | | | | | |
| 3. BD ( 1) O 4 - H 5 | /***. RY*( 1) H 140 | 0.09 | 1.67 | | 0.01 |
| 3. BD ( 1) O 4 - H 5 | /***. RY*( 4) O 154 | 0.15 | 2.15 | | 0.02 |
| 3. BD ( 1) O 4 - H 5 | /***. BD*( 1) H 140 - O 154 | 0.92 | 1.32 | | 0.03 |
| 4. BD ( 1) O 4 - H 6 | /***. RY*( 1) H 140 | 0.08 | 1.67 | | 0.01 |
| 4. BD ( 1) O 4 - H 6 | /***. RY*( 3) H 140 | 0.11 | 2.71 | | 0.02 |
| 4. BD ( 1) O 4 - H 6 | /***. RY*( 4) O 154 | 0.17 | 2.15 | | 0.02 |
| 4. BD ( 1) O 4 - H 6 | /***. BD*( 1) H 140 - O 154 | 0.93 | 1.32 | | 0.03 |
| 596. CR ( 1) O 4 | /***. BD*( 1) H 140 - O 154 | 0.06 | 19.89 | | 0.03 |
| 852. LP ( 1) O 4 | /***. RY*( 2) H 140 | 0.15 | 2.74 | | 0.02 |
| 852. LP ( 1) O 4 | /***. BD*( 1) H 140 - O 154 | 0.63 | 1.24 | | 0.03 |
| 853. LP ( 2) O 4 | /***. RY*( 3) H 140 | 0.14 | 2.29 | | 0.02 |
| 853. LP ( 2) O 4 | /***. BD*( 1) H 140 - O 154 | 12.61 | 0.90 | | 0.10 |
| 853. LP ( 2) O 4 | /***. BD*( 1) O 154 - C 160 | 0.12 | 0.90 | | 0.01 |
| from unit 4 to unit 1 | | | | | |
| 889. LP ( 2) O 165 | /987. RY*( 1) O 4 | 0.06 | 1.38 | | 0.01 |
| **total interaction between unit 1 and 4** | | **16.22** | **42.33** | | **0.34** |
| from unit 1 to unit 5 | | | | | |
| 3. BD ( 1) O 4 - H 5 | /***. RY*( 1) O 292 | 0.06 | 2.09 | | 0.01 |
| 3. BD ( 1) O 4 - H 5 | /***. RY*( 3) O 292 | 0.06 | 2.35 | | 0.01 |
| 852. LP ( 1) O 4 | /***. BD*( 1) C 287 - O 292 | 0.22 | 1.45 | | 0.02 |
| from unit 5 to unit 1 | | | | | |
| 915. LP ( 1) O 292 | /***. BD*( 1) O 4 - H 5 | 0.06 | 1.26 | | 0.01 |
| 916. LP ( 2) O 292 | /***. BD*( 1) O 4 - H 5 | 0.06 | 0.81 | | 0.01 |
| **total interaction between unit 1 and 5** | | **0.46** | **7.96** | | **0.05** |
| from unit 1 to unit 6 | | | | | |
| 4. BD ( 1) O 4 - H 6 | /***. BD*( 1) C 28 - O 33 | 0.08 | 1.58 | | 0.01 |
| 853. LP ( 2) O 4 | /***. BD*( 1) C 28 - O 33 | 0.20 | 1.16 | | 0.01 |
| from unit 6 to unit 1 | | | | | |
| 862. LP ( 2) O 33 | /***. BD*( 1) O 4 - H 6 | 0.12 | 0.86 | | 0.01 |
| **total interaction between unit 1 and 6** | | **0.40** | **3.60** | | **0.03** |
| **325K (Crystal 2)** | | | | | |
| from unit 1 to unit 2 | | | | | |
| 298. BD ( 1) H 269 - O 270 | /***. RY*( 4) H 265 | 0.05 | 3.06 | | 0.01 |
| 906. LP ( 1) O 270 | /***. RY*( 3) H 265 | 0.14 | 2.21 | | 0.02 |
| 906. LP ( 1) O 270 | /***. BD*( 1) H 265 - O 267 | 1.27 | 1.27 | | 0.04 |
| 907. LP ( 2) O 270 | /***. BD*( 1) H 265 - O 267 | 0.26 | 0.94 | | 0.01 |
| **total interaction between 1 and 2** | | **1.72** | **7.48** | | **0.08** |
| from unit 3 to unit 1 | | | | | |
| 299. BD ( 1) H 271 - O 273 | /***. RY*( 4) H 268 | 0.06 | 3.00 | | 0.01 |
| 300. BD ( 1) H 272 - O 273 | /***. RY*( 4) H 268 | 0.06 | 3.02 | | 0.01 |
| 908. LP ( 1) O 273 | /***. RY*( 2) H 268 | 0.05 | 2.01 | | 0.01 |
| 908. LP ( 1) O 273 | /***. BD*( 1) H 268 - O 270 | 1.24 | 1.28 | | 0.04 |
| 909. LP ( 2) O 273 | /***. BD*( 1) H 268 - O 270 | 0.26 | 0.94 | | 0.01 |
| **total interaction between 1 and 3** | | **1.67** | **10.25** | | **0.08** |
| from unit 1 to unit 4 | | | | | |
| 297. BD ( 1) H 268 - O 270 | /984. RY*( 1) H 5 | 0.08 | 1.52 | | 0.01 |
| 297. BD ( 1) H 268 - O 270 | /***. BD*( 1) H 5 - O 24 | 0.54 | 1.30 | | 0.02 |
| 298. BD ( 1) H 269 - O 270 | /984. RY*( 1) H 5 | 0.13 | 1.53 | | 0.01 |
| 298. BD ( 1) H 269 - O 270 | /***. BD*( 1) H 5 - O 24 | 0.11 | 1.32 | | 0.01 |
| 906. LP ( 1) O 270 | /987. RY*( 4) H 5 | 0.05 | 2.73 | | 0.01 |
| 906. LP ( 1) O 270 | /***. BD*( 1) H 5 - O 24 | 0.48 | 1.20 | | 0.02 |
| 906. LP ( 1) O 270 | /***. BD*( 1) O 24 - C 29 | 0.06 | 1.22 | | 0.01 |
| 906. LP ( 1) O 270 | /***. BD*( 1) O 28 - C 29 | 0.05 | 1.51 | | 0.01 |
| 906. LP ( 1) O 270 | /***. BD*( 1) C 29 - C 38 | 0.05 | 1.14 | | 0.01 |
| 907. LP ( 2) O 270 | /986. RY*( 3) H 5 | 0.09 | 2.18 | | 0.01 |
| 907. LP ( 2) O 270 | /***. BD*( 1) H 5 - O 24 | 5.46 | 0.86 | | 0.06 |
| 907. LP ( 2) O 270 | /***. BD*( 1) O 24 - C 29 | 0.11 | 0.88 | | 0.01 |
| from unit 4 to unit 1 | | | | | |
| 5. BD ( 1) H 5 - O 24 | /***. RY*( 1) O 270 | 0.05 | 2.02 | | 0.01 |
| 5. BD ( 1) H 5 - O 24 | /***. RY*( 2) O 270 | 0.06 | 2.03 | | 0.01 |
| **total interaction between 1 and 4** | | **7.32** | **21.44** | | **0.21** |
| from unit 1 to unit 5 | | | | | |
| 298. BD ( 1) H 269 - O 270 | /***. BD*( 1) C 236 - O 242 | 0.20 | 1.56 | | 0.02 |
| 907. LP ( 2) O 270 | /***. BD*( 1) C 236 - O 242 | 0.08 | 1.11 | | 0.01 |
| from unit 5 to unit 1 | | | | | |
| 267. BD ( 1) C 236 - C 239 | /***. BD*( 1) H 269 - O 270 | 0.08 | 1.30 | | 0.01 |
| 902. LP ( 1) O 242 | /***. BD*( 1) H 269 - O 270 | 0.97 | 1.35 | | 0.03 |
| 903. LP ( 2) O 242 | /***. BD*( 1) H 269 - O 270 | 0.93 | 0.91 | | 0.03 |
| **Total interaction between 1 and 5** | | **2.26** | **6.23** | | **0.09** |
| from unit 1 to unit 6 | | | | | |
| 297. BD ( 1) H 268 - O 270 | /***. BD*( 1) O 160 - C 161 | 0.07 | 1.62 | | 0.01 |
| 907. LP ( 2) O 270 | /***. BD*( 1) O 160 - C 161 | 0.19 | 1.18 | | 0.01 |
| from unit 6 to unit 1 | | | | | |
| 885. LP ( 2) O 160 | /***. BD*( 1) H 268 - O 270 | 0.27 | 0.89 | | 0.01 |
| **Total interaction between 1 and 6** | | **0.53** | **3.69** | | **0.04** |
| from unit 1 to unit 7 | | | | | |
| 298. BD ( 1) H 269 - O 270 | /***. RY*( 1) H 308 | 0.08 | 1.60 | | 0.01 |
| 907. LP ( 2) O 270 | /***. RY*( 1) H 308 | 0.07 | 1.14 | | 0.01 |
| **Total interaction between 1 and 7** | | **0.15** | **2.74** | | **0.02** |
| **335K (Crystal 2)** | | | | | |
| from unit 1 to unit 2 | | | | | |
| 850. LP ( 1) O 1 | /987. RY*( 1) O 4 | 0.10 | 1.55 | | 0.01 |
| 850. LP ( 1) O 1 | /***. RY*( 1) H 5 | 0.19 | 1.56 | | 0.02 |
| 850. LP ( 1) O 1 | /***. BD*( 1) O 4 - H 5 | 0.97 | 1.00 | | 0.03 |
| 851. LP ( 2) O 1 | /***. BD*( 1) O 4 - H 5 | 0.06 | 1.21 | | 0.01 |
| **total interaction between fragment 1 and 2** | | **1.32** | **5.32** | | **0.06** |
| from unit 1 to unit 3 | | | | | |
| 1. BD ( 1) O 1 - H 2 | /***. RY*( 2) O 7 | 0.06 | 2.00 | | 0.01 |
| from unit 3 to unit 1 | | | | | |
| 854. LP ( 1) O 7 | /964. RY*( 1) O 1 | 0.10 | 1.56 | | 0.01 |
| 854. LP ( 1) O 7 | /977. RY*( 1) H 2 | 0.18 | 1.55 | | 0.02 |
| 854. LP ( 1) O 7 | /***. BD*( 1) O 1 - H 2 | 0.95 | 1.00 | | 0.03 |
| 855. LP ( 2) O 7 | /***. BD*( 1) O 1 - H 2 | 0.06 | 1.22 | | 0.01 |
|  |  | 1.29 | 5.33 | | 0.06 |
| **total interaction between fragment 1 and 3** | | **1.35** | **7.33** | | **0.07** |
| from unit 1 to unit 4 | | | | | |
| 1. BD ( 1) O 1 - H 2 | /***. RY*( 2) H 256 | 0.14 | 2.62 | | 0.02 |
| 1. BD ( 1) O 1 - H 2 | /***. RY*( 3) H 256 | 0.13 | 2.53 | | 0.02 |
| 1. BD ( 1) O 1 - H 2 | /***. RY*( 4) H 256 | 0.07 | 2.81 | | 0.01 |
| 2. BD ( 1) O 1 - H 3 | /***. RY*( 2) H 256 | 0.13 | 2.64 | | 0.02 |
| 850. LP ( 1) O 1 | /***. BD*( 1) O 255 - H 256 | 0.16 | 1.35 | | 0.01 |
| 851. LP ( 2) O 1 | /***. RY*( 4) O 255 | 0.70 | 1.97 | | 0.03 |
| 851. LP ( 2) O 1 | /***. RY*( 10) O 255 | 0.06 | 4.26 | | 0.02 |
| 851. LP ( 2) O 1 | /***. RY*( 1) H 256 | 0.30 | 1.79 | | 0.02 |
| 851. LP ( 2) O 1 | /***. RY*( 4) H 256 | 0.14 | 2.64 | | 0.02 |
| 851. LP ( 2) O 1 | /***. BD*( 1) O 255 - H 256 | 12.19 | 1.56 | | 0.12 |
| 851. LP ( 2) O 1 | /***. BD*( 1) O 255 - C 261 | 0.33 | 1.11 | | 0.02 |
| from unit 4 to unit 1 | | | | | |
| 907. LP ( 2) O 255 | /964. RY*( 1) O 1 | 0.05 | 1.53 | | 0.01 |
| **total interaction between fragments 1 and 4** | | **14.40** | **26.81** | | **0.31** |
| from unit 1 to unit 5 | | | | | |
| 2. BD ( 1) O 1 - H 3 | /***. BD*( 1) C 112 - O 127 | 0.28 | 1.52 | | 0.02 |
| 851. LP ( 2) O 1 | /***. BD*( 1) C 112 - O 127 | 0.08 | 1.33 | | 0.01 |
| from unit 5 to unit 1 | | | | | |
| 125. BD ( 1) C 112 - C 124 | /***. BD*( 1) O 1 - H 3 | 0.20 | 1.33 | | 0.02 |
| 126. BD ( 1) C 112 - O 127 | /983. RY*( 2) H 3 | 0.06 | 3.31 | | 0.01 |
| 881. LP ( 1) O 127 | /***. BD*( 1) O 1 - H 3 | 3.63 | 1.38 | | 0.06 |
| 882. LP ( 2) O 127 | /964. RY*( 1) O 1 | 0.08 | 1.49 | | 0.01 |
| 882. LP ( 2) O 127 | /983. RY*( 2) H 3 | 0.08 | 2.45 | | 0.01 |
| 882. LP ( 2) O 127 | /***. BD*( 1) O 1 - H 3 | 3.78 | 0.95 | | 0.06 |
| **total interaction between fragment 1 and 5** | | **8.19** | **13.76** | | **0.20** |
| from unit 1 to unit 6 | | | | | |
| 1. BD ( 1) O 1 - H 2 | /***. BD*( 1) C 349 - O 354 | 0.12 | 1.65 | | 0.01 |
| 851. LP ( 2) O 1 | /***. RY*( 3) O 354 | 0.09 | 1.87 | | 0.01 |
| 851. LP ( 2) O 1 | /***. BD*( 1) C 349 - O 354 | 0.11 | 1.47 | | 0.01 |
| from unit 6 to unit 1 | | | | | |
| 389. BD ( 2) C 349 - O 354 | /***. BD*( 1) O 1 - H 2 | 0.07 | 1.07 | | 0.01 |
| 390. BD ( 1) C 349 - C 359 | /***. BD*( 1) O 1 - H 2 | 0.12 | 1.24 | | 0.01 |
| 926. LP ( 1) O 354 | /***. BD*( 1) O 1 - H 2 | 0.83 | 1.34 | | 0.03 |
| 927. LP ( 2) O 354 | /***. BD*( 1) O 1 - H 2 | 1.41 | 0.92 | | 0.03 |
| **total interaction between fragments 1 and 6** | | **2.75** | **9.56** | | **0.12** |
| **from unit 1 to unit 7** | | | | | |
| **850. LP ( 1) O 1** | **/***. RY*( 1) H 395** | **0.09** | **1.17** | | **0.01** |
| **from unit 1 to unit 7** | | | | | |
| **850. LP ( 1) O 1** | **/***. RY*( 1) H 154** | **0.05** | **1.15** | | **0.01** |
| **337K (Crystal 3)** | | | | | |
| **from unit 1 to unit 2** | | | | | |
| 850. LP ( 1) O 3 | /992. RY*( 1) H 5 | 0.15 | 1.39 | | 0.01 |
| 850. LP ( 1) O 3 | /997. RY*( 1) O 6 | 0.14 | 1.53 | | 0.01 |
| 850. LP ( 1) O 3 | /***. BD*( 1) H 4 - O 6 | 0.06 | 1.00 | | 0.01 |
| 850. LP ( 1) O 3 | /***. BD*( 1) H 5 - O 6 | 0.71 | 0.92 | | 0.02 |
| 851. LP ( 2) O 3 | /***. BD*( 1) H 5 - O 6 | 0.15 | 1.23 | | 0.01 |
| **total interaction between units 1 and 2** | | **1.21** | **6.07** | | **0.07** |
| **from unit 1 to unit 3** | | | | | |
| 2. BD ( 1) H 2 - O 3 | /***. RY*( 2) O 317 | 0.05 | 2.00 | | 0.01 |
| **from unit 3 to unit 1** | | | | | |
| 917. LP ( 1) O 317 | /969. RY*( 1) H 2 | 0.19 | 1.40 | | 0.02 |
| 917. LP ( 1) O 317 | /974. RY*( 1) O 3 | 0.14 | 1.57 | | 0.01 |
| 917. LP ( 1) O 317 | /***. BD*( 1) H 1 - O 3 | 0.06 | 1.00 | | 0.01 |
| 917. LP ( 1) O 317 | /***. BD*( 1) H 2 - O 3 | 0.71 | 0.92 | | 0.02 |
| 918. LP ( 2) O 317 | /***. BD*( 1) H 2 - O 3 | 0.15 | 1.23 | | 0.01 |
| **total interaction between units 1 and 3** | | **1.30** | **8.12** | | **0.08** |
| from unit 1 to unit 4 | | | | | |
| 1. BD ( 1) H 1 - O 3 | /***. RY*( 2) H 206 | 0.23 | 2.65 | | 0.02 |
| 1. BD ( 1) H 1 - O 3 | /***. RY*( 4) H 206 | 0.09 | 2.85 | | 0.01 |
| 2. BD ( 1) H 2 - O 3 | /***. RY*( 2) H 206 | 0.20 | 2.61 | | 0.02 |
| 2. BD ( 1) H 2 - O 3 | /***. RY*( 3) H 206 | 0.07 | 2.55 | | 0.01 |
| 2. BD ( 1) H 2 - O 3 | /***. RY*( 4) H 206 | 0.08 | 2.81 | | 0.01 |
| 850. LP ( 1) O 3 | /***. BD*( 1) O 205 - H 206 | 0.08 | 1.30 | | 0.01 |
| 851. LP ( 2) O 3 | /***. RY*( 4) O 205 | 0.60 | 1.96 | | 0.03 |
| 851. LP ( 2) O 3 | /***. RY*( 10) O 205 | 0.05 | 4.33 | | 0.01 |
| 851. LP ( 2) O 3 | /***. RY*( 1) H 206 | 0.34 | 1.82 | | 0.02 |
| 851. LP ( 2) O 3 | /***. RY*( 4) H 206 | 0.10 | 2.69 | | 0.02 |
| 851. LP ( 2) O 3 | /***. BD*( 1) O 205 - H 206 | 11.68 | 1.61 | | 0.12 |
| 851. LP ( 2) O 3 | /***. BD*( 1) O 205 - C 223 | 0.31 | 1.15 | | 0.02 |
| from unit 4 to unit 1 | | | | | |
| 896. LP ( 2) O 205 | /974. RY*( 1) O 3 | 0.05 | 1.60 | | 0.01 |
| 896. LP ( 2) O 205 | /***. BD*( 1) H 2 - O 3 | 0.05 | 0.95 | | 0.01 |
| **total interaction between units 1 and 4** | | **13.93** | **30.88** | | **0.33** |
| from unit 1 to unit 5 | | | | | |
| 1. BD ( 1) H 1 - O 3 | /***. BD*( 1) C 24 - O 28 | 0.22 | 1.55 | | 0.02 |
| 851. LP ( 2) O 3 | /***. BD*( 1) C 24 - O 28 | 0.06 | 1.39 | | 0.01 |
| from unit 5 to unit 1 | | | | | |
| 24. BD ( 1) C 18 - C 24 | /***. BD*( 1) H 1 - O 3 | 0.20 | 1.35 | | 0.02 |
| 29. BD ( 2) C 24 - O 28 | /965. RY*( 2) H 1 | 0.08 | 2.38 | | 0.01 |
| 29. BD ( 2) C 24 - O 28 | /***. BD*( 1) H 1 - O 3 | 0.48 | 1.09 | | 0.02 |
| 861. LP ( 1) O 28 | /974. RY*( 1) O 3 | 0.05 | 1.98 | | 0.01 |
| 861. LP ( 1) O 28 | /***. BD*( 1) H 1 - O 3 | 3.38 | 1.41 | | 0.06 |
| 862. LP ( 2) O 28 | /964. RY*( 1) H 1 | 0.06 | 1.79 | | 0.01 |
| 862. LP ( 2) O 28 | /966. RY*( 3) H 1 | 0.05 | 2.16 | | 0.01 |
| 862. LP ( 2) O 28 | /974. RY*( 1) O 3 | 0.09 | 1.55 | | 0.01 |
| 862. LP ( 2) O 28 | /***. BD*( 1) H 1 - O 3 | 3.70 | 0.98 | | 0.06 |
| ***. BD*( 2) C 24 - O 28 | /***. BD*( 1) H 1 - O 3 | 0.13 | 0.56 | | 0.02 |
| **total interaction between unit 1 and 5** | | **8.50** | **18.19** | | **0.25** |
| from unit 1 to unit 6 | | | | | |
| 2. BD ( 1) H 2 - O 3 | /***. BD*( 1) O 300 - C 311 | 0.13 | 1.65 | | 0.01 |
| 851. LP ( 2) O 3 | /***. RY*( 3) O 300 | 0.08 | 1.87 | | 0.01 |
| 851. LP ( 2) O 3 | /***. BD*( 1) O 300 - C 311 | 0.10 | 1.53 | | 0.01 |
| from unit 6 to unit 1 | | | | | |
| 337. BD ( 2) O 300 - C 311 | /***. BD*( 1) H 2 - O 3 | 0.16 | 1.04 | | 0.01 |
| 345. BD ( 1) C 311 - C 312 | /***. BD*( 1) H 2 - O 3 | 0.11 | 1.22 | | 0.01 |
| 915. LP ( 1) O 300 | /***. BD*( 1) H 2 - O 3 | 0.79 | 1.31 | | 0.03 |
| 916. LP ( 2) O 300 | /***. BD*( 1) H 2 - O 3 | 1.21 | 0.90 | | 0.03 |
| **total interaction between units 1 and 6** | | **2.58** | **9.52** | | **0.12** |
| **from unit 1 to unit 7** | | | | | |
| **850. LP ( 1) O 3** | **/***. RY*( 1) H 129** | **0.09** | **1.12** | | **0.01** |
| **339K (Crystal 3)** | | | | | |
| **from unit 1 to unit 2** | | | | | |
| 854. LP ( 1) O 7 | /964. RY*( 1) O 1 | 0.09 | | 1.56 | 0.01 |
| 854. LP ( 1) O 7 | /982. RY*( 1) H 3 | 0.12 | | 1.52 | 0.01 |
| 854. LP ( 1) O 7 | /***. BD*( 1) O 1 - H 2 | 0.07 | | 1.01 | 0.01 |
| 854. LP ( 1) O 7 | /***. BD*( 1) O 1 - H 3 | 0.36 | | 0.96 | 0.02 |
| **total interaction between units 1 and 2** | | **0.64** | | **5.05** | **0.05** |
| **from unit 3 to unit 1** | | | | | |
| 852. LP ( 1) O 4 | /***. RY*( 1) O 7 | 0.09 | | 1.58 | 0.01 |
| 852. LP ( 1) O 4 | /***. RY*( 1) H 9 | 0.14 | | 1.58 | 0.01 |
| 852. LP ( 1) O 4 | /***. BD*( 1) O 7 - H 8 | 0.07 | | 1.02 | 0.01 |
| 852. LP ( 1) O 4 | /***. BD*( 1) O 7 - H 9 | 0.35 | | 0.96 | 0.02 |
| **total interaction between units 1 and 3** | | **0.65** | | **5.14** | **0.05** |
| **from unit 1 to unit 4** | | | | | |
| 5. BD ( 1) O 7 - H 8 | /***. RY*( 2) H 232 | 0.22 | | 2.88 | 0.02 |
| 5. BD ( 1) O 7 - H 8 | /***. BD*( 1) H 232 - O 260 | 0.19 | | 1.33 | 0.01 |
| 6. BD ( 1) O 7 - H 9 | /***. RY*( 2) H 232 | 0.36 | | 2.85 | 0.03 |
| 6. BD ( 1) O 7 - H 9 | /***. RY*( 3) H 232 | 0.05 | | 2.57 | 0.01 |
| 597. CR ( 1) O 7 | /***. BD*( 1) H 232 - O 260 | 0.20 | | 19.87 | 0.06 |
| 854. LP ( 1) O 7 | /***. RY*( 2) H 232 | 0.05 | | 2.46 | 0.01 |
| 854. LP ( 1) O 7 | /***. RY*( 4) H 232 | 0.06 | | 2.44 | 0.01 |
| 854. LP ( 1) O 7 | /***. BD*( 1) H 232 - O 260 | 0.40 | | 0.91 | 0.02 |
| 854. LP ( 1) O 7 | /***. BD*( 1) C 261 - O 262 | 0.05 | | 1.26 | 0.01 |
| 855. LP ( 2) O 7 | /***. RY*( 2) H 232 | 0.10 | | 2.70 | 0.02 |
| 855. LP ( 2) O 7 | /***. RY*( 4) H 232 | 0.06 | | 2.68 | 0.01 |
| 855. LP ( 2) O 7 | /***. RY*( 5) H 232 | 0.10 | | 3.16 | 0.02 |
| 855. LP ( 2) O 7 | /***. RY*( 4) O 260 | 0.26 | | 2.48 | 0.02 |
| 855. LP ( 2) O 7 | /***. RY*( 5) O 260 | 0.08 | | 4.32 | 0.02 |
| 855. LP ( 2) O 7 | /***. BD*( 1) H 232 - O 260 | 21.12 | | 1.15 | 0.14 |
| 855. LP ( 2) O 7 | /***. BD*( 1) O 260 - C 261 | 0.12 | | 1.11 | 0.01 |
| **from unit 4 to unit 1** | | | | | |
| 254. BD ( 1) H 232 - O 260 | /***. RY*( 2) O 7 | 0.05 | | 2.12 | 0.01 |
| 254. BD ( 1) H 232 - O 260 | /***. BD*( 1) O 7 - H 9 | 0.20 | | 1.40 | 0.02 |
| 907. LP ( 2) O 260 | /***. RY*( 1) O 7 | 0.06 | | 1.54 | 0.01 |
| 907. LP ( 2) O 260 | /***. BD*( 1) O 7 - H 9 | 0.08 | | 0.92 | 0.01 |
| **Total interaction between units 1 and 4** | | **23.81** | | **60.15** | **0.45** |
| **from unit 1 to unit 5** | | | | | |
| 5. BD ( 1) O 7 - H 8 | /***. BD*( 1) C 290 - O 309 | 0.28 | | 1.53 | 0.02 |
| 855. LP ( 2) O 7 | /***. RY*( 1) H 281 | 0.08 | | 1.84 | 0.01 |
| 855. LP ( 2) O 7 | /***. BD*( 1) C 290 - O 309 | 0.10 | | 1.35 | 0.01 |
| **from unit 5 to unit 1** | | | | | |
| 322. BD ( 1) C 290 - C 292 | /***. BD*( 1) O 7 - H 8 | 0.20 | | 1.32 | 0.02 |
| 324. BD ( 1) C 290 - O 309 | /***. RY*( 1) H 8 | 0.07 | | 2.90 | 0.01 |
| 325. BD ( 2) C 290 - O 309 | /***. RY*( 1) H 8 | 0.05 | | 2.15 | 0.01 |
| 325. BD ( 2) C 290 - O 309 | /***. BD*( 1) O 7 - H 8 | 0.14 | | 1.05 | 0.01 |
| 917. LP ( 1) O 309 | /***. BD*( 1) O 7 - H 8 | 4.05 | | 1.38 | 0.07 |
| 918. LP ( 2) O 309 | /***. RY*( 1) O 7 | 0.07 | | 1.51 | 0.01 |
| 918. LP ( 2) O 309 | /***. RY*( 2) O 7 | 0.06 | | 1.61 | 0.01 |
| 918. LP ( 2) O 309 | /***. RY*( 1) H 8 | 0.05 | | 2.05 | 0.01 |
| 918. LP ( 2) O 309 | /***. BD*( 1) O 7 - H 8 | 3.66 | | 0.94 | 0.05 |
| **Total interaction between units 1 and 5** | | **8.81** | | **19.63** | **0.24** |
| **from unit 1 to unit 6** | | | | | |
| 6. BD ( 1) O 7 - H 9 | /***. BD*( 1) C 85 - O 86 | 0.16 | | 1.66 | 0.02 |
| 855. LP ( 2) O 7 | /***. RY*( 3) O 86 | 0.06 | | 1.93 | 0.01 |
| 855. LP ( 2) O 7 | /***. BD*( 1) C 85 - O 86 | 0.08 | | 1.51 | 0.01 |
| **from unit 6 to unit 1** | | | | | |
| 95. BD ( 2) C 85 - O 86 | /***. BD*( 1) O 7 - H 9 | 0.28 | | 1.03 | 0.02 |
| 96. BD ( 1) C 85 - C 95 | /***. BD*( 1) O 7 - H 9 | 0.18 | | 1.21 | 0.01 |
| 872. LP ( 1) O 86 | /***. BD*( 1) O 7 - H 9 | 2.00 | | 1.30 | 0.05 |
| 873. LP ( 2) O 86 | /***. BD*( 1) O 7 - H 9 | 1.58 | | 0.89 | 0.04 |
| ***. BD*( 2) C 85 - O 86 | /***. BD*( 1) O 7 - H 9 | 0.09 | | 0.44 | 0.02 |
| **Total interaction between units 1 and 6** | | **4.43** | | **9.97** | **0.16** |
| **from unit 1 to unit 7** | | | | | |
| 854. LP ( 1) O 7 | /***. RY*( 1) H 132 | **0.07** | | **1.17** | **0.01** |
| **from unit 3 to unit 4** | | | | | |
| 854. LP ( 1) O 7 | /***. RY*( 1) H 12 | **0.06** | | **1.38** | **0.01** |
| **340K (Crystal 3)** | | | | | |
| **from unit 1 to unit 2** | | | | | |
| 904. LP ( 1) O 267 | /***. RY*( 1) H 269 | 0.11 | 1.35 | | 0.01 |
| 904. LP ( 1) O 267 | /***. RY*( 1) O 270 | 0.16 | 1.43 | | 0.01 |
| 904. LP ( 1) O 267 | /***. BD*( 1) H 268 - O 270 | 0.09 | 0.95 | | 0.01 |
| 904. LP ( 1) O 267 | /***. BD*( 1) H 269 - O 270 | 0.13 | 0.93 | | 0.01 |
| **total interaction between unit 1 and 2** | | **0.49** | **4.66** | | **0.04** |
| **from unit 3 to unit 1** | | | | | |
| 926. LP ( 1) O 361 | /***. RY*( 1) H 266 | 0.15 | 1.40 | | 0.01 |
| 926. LP ( 1) O 361 | /***. RY*( 1) O 267 | 0.16 | 1.45 | | 0.01 |
| 926. LP ( 1) O 361 | /***. BD*( 1) H 265 - O 267 | 0.09 | 0.95 | | 0.01 |
| 926. LP ( 1) O 361 | /***. BD*( 1) H 266 - O 267 | 0.13 | 0.93 | | 0.01 |
| **total interaction between unit 1 and 3** | | **0.53** | **4.73** | | **0.04** |
| **from unit 4 to unit 1** | | | | | |
| 247. BD ( 1) H 222 - O 249 | /***. RY*( 2) O 267 | 0.06 | 2.19 | | 0.01 |
| 247. BD ( 1) H 222 - O 249 | /***. BD*( 1) H 266 - O 267 | 0.37 | 1.41 | | 0.02 |
| 900. LP ( 1) O 249 | /***. BD*( 1) H 266 - O 267 | 0.06 | 1.17 | | 0.01 |
| 901. LP ( 2) O 249 | /***. RY*( 1) O 267 | 0.07 | 1.45 | | 0.01 |
| 901. LP ( 2) O 249 | /***. BD*( 1) H 266 - O 267 | 0.06 | 0.93 | | 0.01 |
| **from unit 1 to unit 4** | | | | | |
| 295. BD ( 1) H 265 - O 267 | /***. RY*( 2) H 222 | 0.22 | 2.73 | | 0.02 |
| 295. BD ( 1) H 265 - O 267 | /***. BD*( 1) H 222 - O 249 | 0.56 | 1.33 | | 0.03 |
| 296. BD ( 1) H 266 - O 267 | /***. RY*( 1) H 222 | 0.17 | 1.95 | | 0.02 |
| 296. BD ( 1) H 266 - O 267 | /***. RY*( 2) H 222 | 0.71 | 2.72 | | 0.04 |
| 296. BD ( 1) H 266 - O 267 | /***. RY*( 5) H 222 | 0.07 | 3.29 | | 0.01 |
| 721. CR ( 1) O 267 | /***. BD*( 1) H 222 - O 249 | 0.34 | 19.89 | | 0.08 |
| 905. LP ( 2) O 267 | /***. RY*( 2) H 222 | 0.22 | 2.61 | | 0.02 |
| 905. LP ( 2) O 267 | /***. RY*( 4) H 222 | 0.10 | 2.79 | | 0.02 |
| 905. LP ( 2) O 267 | /***. RY*( 5) H 222 | 0.13 | 3.18 | | 0.02 |
| 905. LP ( 2) O 267 | /***. RY*( 4) O 249 | 0.40 | 2.28 | | 0.03 |
| 905. LP ( 2) O 267 | /***. BD*( 1) H 222 - O 249 | 27.37 | 1.21 | | 0.16 |
| 905. LP ( 2) O 267 | /***. BD*( 1) O 249 - C 261 | 0.13 | 1.17 | | 0.01 |
| **total interaction between unit 1 and 4** | | **31.04** | **52.30** | | **0.50** |
| **from unit 5 to unit 1** | | | | | |
| 23. BD ( 1) C 18 - C 20 | /***. BD*( 1) H 265 - O 267 | 0.13 | 1.29 | | 0.01 |
| 25. BD ( 1) C 18 - O 40 | /***. RY*( 3) H 265 | 0.06 | 3.08 | | 0.01 |
| 26. BD ( 2) C 18 - O 40 | /***. RY*( 2) H 265 | 0.08 | 2.49 | | 0.01 |
| 26. BD ( 2) C 18 - O 40 | /***. BD*( 1) H 265 - O 267 | 0.45 | 1.03 | | 0.02 |
| 857. LP ( 1) O 40 | /***. BD*( 1) H 265 - O 267 | 2.51 | 1.35 | | 0.05 |
| 858. LP ( 2) O 40 | /***. RY*( 1) O 267 | 0.06 | 1.41 | | 0.01 |
| 858. LP ( 2) O 40 | /***. BD*( 1) H 265 - O 267 | 1.70 | 0.91 | | 0.04 |
| ***. BD*( 2) C 18 - O 40 | /***. BD*( 1) H 265 - O 267 | 0.13 | 0.50 | | 0.02 |
| **from unit 1 to unit 5** | | | | | |
| 295. BD ( 1) H 265 - O 267 | /***. BD*( 1) C 18 - O 40 | 0.24 | 1.55 | | 0.02 |
| 905. LP ( 2) O 267 | /***. RY*( 1) H 9 | 0.06 | 1.95 | | 0.01 |
| 905. LP ( 2) O 267 | /***. BD*( 1) C 18 - O 40 | 0.09 | 1.43 | | 0.01 |
| **total interaction between units 1 and 5** | | **5.51** | **16.99** | | **0.21** |
| **from unit 6 to unit 1** | | | | | |
| 389. BD ( 1) O 350 - C 355 | /***. RY*( 4) H 266 | 0.05 | 3.44 | | 0.01 |
| 390. BD ( 2) O 350 - C 355 | /***. BD*( 1) H 266 - O 267 | 0.34 | 1.04 | | 0.02 |
| 394. BD ( 1) C 355 - C 356 | /***. BD*( 1) H 266 - O 267 | 0.15 | 1.23 | | 0.01 |
| 924. LP ( 1) O 350 | /***. BD*( 1) H 266 - O 267 | 1.78 | 1.31 | | 0.04 |
| 925. LP ( 2) O 350 | /***. BD*( 1) H 266 - O 267 | 1.13 | 0.90 | | 0.03 |
| ***. BD*( 2) O 350 - C 355 | /***. BD*( 1) H 266 - O 267 | 0.09 | 0.45 | | 0.02 |
| **from unit 1 to unit 6** | | | | | |
| 296. BD ( 1) H 266 - O 267 | /***. BD*( 1) O 350 - C 355 | 0.14 | 1.69 | | 0.01 |
| 905. LP ( 2) O 267 | /***. BD*( 1) O 350 - C 355 | 0.07 | 1.58 | | 0.01 |
| **total interaction between unit 1 and 6** | | **3.75** | **11.64** | | **0.16** |
| from unit 1 to unit 7 | | | | | |
| 904. LP ( 1) O 267 | /***. RY*( 1) H 170 | **0.08** | **1.15** | | **0.01** |

^#^E(*i*) corresponds to Lewis type “filled” donor orbitals. E(*j*) corresponds to non-Lewis type “unfilled” acceptor orbitals. LP = lone-pair, LP* = anti-bonding lone pair, BD = 2-center bond, BD* = 2-center anti-bond, RY = Rydberg orbital, RY* = Rydberg anti-bond orbitals.

For each donor NBO (*i*) and acceptor NBO (*j*), the stabilization energy *E*(2) associated with *i* → *j* delocalization is determined as,

$$E\left( 2 \right)= E_{ij}^{(2)} =\frac{q_{i}F\left( i,j \right)^{2}}{{}_{j}-{}_{i}}$$

Where, *q*_i_: is the donor orbital occupancy.

*ε*_i_, *ε*_j_ are diagonal elements (orbital energies) of NBO Fock matrix.

*F*(*i*,*j*) is the off-diagonal NBO Fock matrix elements.

Table cells highlighted with yellow color suggest interactions between the water molecules.

**Table S10.** The intermolecular interaction energy of the interactions between the BGPHEOH derivatives. NBO calculation with BGPHEOH structures obtained at different temperatures from single crystal XRD. Second Order Perturbation Theory Analysis of Fock Matrix in NBO Basis. Threshold for printing: E(2)>0.50 kcal/mol.

| **Donor NBO’s (i)** | **Acceptor NBO’s (j)** | **E(2) (kcal/mol)** | **E(j) – E(i)**  **(a.u.)** | | **F(i, j)**  **(a.u.)** |
| --- | --- | --- | --- | --- | --- |
| **296K (Crystal 2)** | | | | | |
| Interaction between unit translated Molecule 1‘s | | | | | |
| **from unit 5 to unit 9** | | | | | |
| 162. BD ( 1) O 147 - C 148 | /***. RY*( 1) H 186 | 0.28 | 1.88 | | 0.02 |
| 182. BD ( 1) O 161 - H 162 | /***. RY*( 1) H 198 | 0.05 | 1.79 | | 0.01 |
| 188. BD ( 1) C 170 - H 171 | /***. RY*( 2) H 212 | 0.09 | 2.44 | | 0.01 |
| 192. BD ( 1) C 174 - C 182 | /***. RY*( 3) C 208 | 0.05 | 1.94 | | 0.01 |
| 192. BD ( 1) C 174 - C 182 | /***. RY*( 1) H 209 | 0.05 | 1.61 | | 0.01 |
| 193. BD ( 2) C 174 - C 182 | /***. BD*( 2) C 201 - C 208 | 0.25 | 0.39 | | 0.01 |
| 194. BD ( 1) C 176 - H 177 | /***. RY*( 2) H 213 | 0.08 | 2.46 | | 0.01 |
| 200. BD ( 1) C 182 - H 183 | /***. BD*( 2) C 201 - C 208 | 0.06 | 0.75 | | 0.01 |
| 886. LP ( 1) O 147 | /***. RY*( 1) H 186 | 0.14 | 1.64 | | 0.01 |
| 886. LP ( 1) O 147 | /***. RY*( 2) N 189 | 0.06 | 2.04 | | 0.01 |
| 886. LP ( 1) O 147 | /***. BD*( 1) H 186 - N 189 | 1.05 | 1.69 | | 0.04 |
| 886. LP ( 1) O 147 | /***. BD*( 1) N 189 - C 192 | 0.06 | 1.36 | | 0.01 |
| 887. LP ( 2) O 147 | /***. RY*( 1) H 204 | 0.05 | 1.13 | | 0.01 |
| 887. LP ( 2) O 147 | /***. BD*( 1) H 186 - N 189 | 0.07 | 1.23 | | 0.01 |
| 889. LP ( 2) O 161 | /***. RY*( 1) H 198 | 0.08 | 1.20 | | 0.01 |
| 889. LP ( 2) O 161 | /***. BD*( 1) C 197 - H 198 | 0.07 | 1.09 | | 0.01 |
| ***. BD*( 2) O 147 - C 148 | /***. RY*( 1) H 186 | 0.32 | 0.70 | | 0.03 |
| ***. BD*( 2) O 147 - C 148 | /***. RY*( 1) H 195 | 0.07 | 0.70 | | 0.02 |
| ***. BD*( 2) O 147 - C 148 | /***. RY*( 1) H 209 | 0.06 | 0.70 | | 0.02 |
| ***. BD*( 2) O 147 - C 148 | /***. BD*( 1) H 186 - N 189 | 0.13 | 0.75 | | 0.02 |
| ***. BD*( 2) C 174 - C 182 | /***. BD*( 2) C 201 - C 208 | 1.30 | 0.03 | | 0.01 |
| ***. BD*( 2) C 174 - C 182 | /***. BD*( 2) C 224 - C 228 | 0.11 | 0.02 | | 0.00 |
| ***. BD*( 2) C 180 - C 184 | /***. BD*( 2) C 224 - C 228 | 0.09 | 0.01 | | 0.00 |
| **from unit 9 to unit 5** | | | | | |
| 227. BD ( 2) C 201 - C 208 | /***. BD*( 2) C 174 - C 182 | 0.16 | 0.35 | | 0.01 |
| 232. BD ( 1) C 208 - H 209 | /***. RY*( 3) C 182 | 0.06 | 1.44 | | 0.01 |
| 235. BD ( 1) C 210 - H 212 | /***. RY*( 1) H 171 | 0.07 | 1.38 | | 0.01 |
| 236. BD ( 1) C 210 - H 213 | /***. RY*( 1) H 177 | 0.10 | 1.37 | | 0.01 |
| **Total interaction between hydrogen bonded molecule 1’s** | | **4.96** | **32.09** | | **0.33** |
| Interaction between unit translated Molecule 2‘s | | | | | |
| **from unit 7 to unit 8** | | | | | |
| 256. BD ( 1) O 233 - C 239 | /***. RY*( 1) H 495 | 0.23 | 1.98 | | 0.02 |
| 256. BD ( 1) O 233 - C 239 | /***. RY*( 2) H 495 | 0.08 | 3.28 | | 0.02 |
| 256. BD ( 1) O 233 - C 239 | /***. BD*( 1) H 495 - N 496 | 0.06 | 2.30 | | 0.01 |
| 274. BD ( 1) C 246 - C 262 | /***. RY*( 1) C 517 | 0.05 | 2.31 | | 0.01 |
| 274. BD ( 1) C 246 - C 262 | /***. RY*( 2) C 517 | 0.08 | 2.14 | | 0.01 |
| 275. BD ( 2) C 246 - C 262 | /***. BD*( 2) C 509 - C 517 | 0.36 | 0.37 | | 0.01 |
| 275. BD ( 2) C 246 - C 262 | /***. BD*( 2) C 514 - C 532 | 0.08 | 0.37 | | 0.01 |
| 293. BD ( 1) C 262 - H 263 | /***. BD*( 2) C 514 - C 532 | 0.05 | 0.74 | | 0.01 |
| 294. BD ( 1) C 264 - H 265 | /***. RY*( 2) H 537 | 0.07 | 2.56 | | 0.01 |
| 296. BD ( 1) C 264 - H 267 | /***. RY*( 1) H 495 | 0.14 | 1.44 | | 0.01 |
| 902. LP ( 1) O 233 | /***. RY*( 1) H 495 | 0.08 | 1.63 | | 0.01 |
| 902. LP ( 1) O 233 | /***. BD*( 1) H 495 - N 496 | 0.48 | 1.95 | | 0.03 |
| 902. LP ( 1) O 233 | /***. BD*( 1) N 496 - C 503 | 0.07 | 1.36 | | 0.01 |
| 903. LP ( 2) O 233 | /***. BD*( 1) H 495 - N 496 | 0.07 | 1.49 | | 0.01 |
| 903. LP ( 2) O 233 | /***. BD*( 1) C 506 - H 508 | 0.12 | 1.02 | | 0.01 |
| 904. LP ( 1) O 234 | /***. RY*( 1) H 495 | 0.05 | 1.46 | | 0.01 |
| ***. BD*( 2) C 245 - C 253 | /***. BD*( 2) C 509 - C 517 | 0.09 | 0.01 | | 0.00 |
| ***. BD*( 2) C 246 - C 262 | /***. BD*( 2) C 514 - C 532 | 1.65 | 0.02 | | 0.01 |
| ***. BD*( 2) C 250 - C 268 | /***. BD*( 2) C 509 - C 517 | 0.05 | 0.01 | | 0.00 |
| **from unit 8 to unit 7** | | | | | |
| 579. BD ( 2) C 514 - C 532 | /***. BD*( 2) C 246 - C 262 | 0.10 | 0.35 | | 0.01 |
| 581. BD ( 1) C 517 - C 532 | /***. RY*( 1) C 262 | 0.06 | 2.32 | | 0.01 |
| 581. BD ( 1) C 517 - C 532 | /***. RY*( 3) C 262 | 0.09 | 1.49 | | 0.01 |
| 593. BD ( 1) C 534 - H 536 | /***. RY*( 1) H 259 | 0.17 | 1.43 | | 0.01 |
| 593. BD ( 1) C 534 - H 536 | /***. RY*( 2) H 259 | 0.07 | 3.05 | | 0.01 |
| 594. BD ( 1) C 534 - H 537 | /***. RY*( 1) H 265 | 0.06 | 1.50 | | 0.01 |
| 594. BD ( 1) C 534 - H 537 | /***. RY*( 2) H 265 | 0.07 | 2.56 | | 0.01 |
| **Total interaction between hydrogen bonded molecule 2’s** | | **4.48** | **39.14** | | **0.27** |
| Interaction between Molecule 1 and 2 | | | | | |
| **from unit 5 to unit 6** | | | | | |
| 223. BD ( 1) C 200 - O 205 | /***. BD*( 1) C 512 - O 516 | 0.06 | 1.75 | | 0.01 |
| 231. BD ( 1) O 205 - H 206 | /***. RY*( 1) H 520 | 0.12 | 1.81 | | 0.01 |
| 231. BD ( 1) O 205 - H 206 | /***. BD*( 1) C 512 - O 516 | 0.12 | 1.70 | | 0.01 |
| **from unit 6 to unit 5** | | | | | |
| 560. BD ( 1) O 504 - C 512 | /***. BD*( 1) O 205 - H 206 | 0.11 | 2.02 | | 0.01 |
| 578. BD ( 1) C 514 - C 532 | /***. RY*( 1) H 216 | 0.06 | 1.52 | | 0.01 |
| 962. LP ( 1) O 516 | /***. RY*( 4) O 205 | 0.09 | 2.11 | | 0.01 |
| 962. LP ( 1) O 516 | /***. RY*( 1) H 206 | 0.10 | 2.25 | | 0.01 |
| 962. LP ( 1) O 516 | /***. BD*( 1) C 200 - O 205 | 0.09 | 1.28 | | 0.01 |
| 962. LP ( 1) O 516 | /***. BD*( 1) O 205 - H 206 | 3.51 | 1.73 | | 0.07 |
| 963. LP ( 2) O 516 | /***. RY*( 4) O 205 | 0.34 | 1.67 | | 0.02 |
| 963. LP ( 2) O 516 | /***. RY*( 1) H 206 | 0.23 | 1.81 | | 0.02 |
| 963. LP ( 2) O 516 | /***. RY*( 4) H 206 | 0.24 | 2.35 | | 0.02 |
| 963. LP ( 2) O 516 | /***. BD*( 1) C 200 - O 205 | 0.19 | 0.84 | | 0.01 |
| 963. LP ( 2) O 516 | /***. BD*( 1) O 205 - H 206 | 4.72 | 1.29 | | 0.07 |
| **Total interaction between hydrogen bonded molecule 1 and 2** | | **9.98** | **24.13** | | **0.31** |
| **305K (Crystal 2)** | | | | | |
| **Interaction between unit translated Molecule 1‘s** | | | | | |
| **from unit 9 to unit 5** | | | | | |
| 1. BD ( 1) H 1 - C 17 | /***. BD*( 1) H 453 - C 460 | 0.19 | 1.05 | | 0.01 |
| 32. BD ( 2) C 22 - C 27 | /***. BD*( 2) C 469 - C 482 | 0.09 | 0.36 | | 0.01 |
| 38. BD ( 1) C 25 - C 27 | /***. RY*( 1) C 482 | 0.06 | 2.34 | | 0.01 |
| 38. BD ( 1) C 25 - C 27 | /***. RY*( 3) C 482 | 0.08 | 1.49 | | 0.01 |
| 49. BD ( 1) C 41 - H 44 | /***. RY*( 1) H 479 | 0.06 | 1.52 | | 0.01 |
| 49. BD ( 1) C 41 - H 44 | /***. RY*( 2) H 479 | 0.07 | 2.57 | | 0.01 |
| **from unit 5 to unit 9** | | | | | |
| 500. BD ( 1) H 453 - C 460 | /***. BD*( 1) H 1 - C 17 | 0.15 | 1.05 | | 0.01 |
| 505. BD ( 1) H 458 - C 465 | /***. BD*( 1) H 5 - C 35 | 0.08 | 1.05 | | 0.01 |
| 512. BD ( 1) O 462 - C 463 | /999. RY*( 1) H 8 | 0.21 | 1.99 | | 0.02 |
| 512. BD ( 1) O 462 - C 463 | /***. RY*( 2) H 8 | 0.10 | 3.45 | | 0.02 |
| 524. BD ( 1) C 469 - C 482 | /***. RY*( 3) C 25 | 0.12 | 1.53 | | 0.01 |
| 525. BD ( 2) C 469 - C 482 | /***. BD*( 2) C 18 - C 25 | 0.35 | 0.38 | | 0.01 |
| 525. BD ( 2) C 469 - C 482 | /***. BD*( 2) C 22 - C 27 | 0.07 | 0.38 | | 0.01 |
| 536. BD ( 1) C 478 - H 479 | /***. RY*( 2) H 44 | 0.07 | 2.52 | | 0.01 |
| 538. BD ( 1) C 478 - H 481 | /999. RY*( 1) H 8 | 0.15 | 1.41 | | 0.01 |
| 947. LP ( 1) O 461 | /999. RY*( 1) H 8 | 0.05 | 1.43 | | 0.01 |
| 949. LP ( 1) O 462 | /999. RY*( 1) H 8 | 0.10 | 1.58 | | 0.01 |
| 949. LP ( 1) O 462 | /***. RY*( 2) N 10 | 0.05 | 1.97 | | 0.01 |
| 949. LP ( 1) O 462 | /***. BD*( 1) H 1 - C 17 | 0.27 | 1.27 | | 0.02 |
| 949. LP ( 1) O 462 | /***. BD*( 1) H 8 - N 10 | 0.86 | 1.70 | | 0.03 |
| 949. LP ( 1) O 462 | /***. BD*( 1) N 10 - C 14 | 0.06 | 1.35 | | 0.01 |
| 950. LP ( 2) O 462 | /***. BD*( 1) H 1 - C 17 | 0.45 | 0.82 | | 0.02 |
| 950. LP ( 2) O 462 | /***. BD*( 1) H 8 - N 10 | 0.12 | 1.25 | | 0.01 |
| ***. BD*( 2) C 467 - C 474 | /***. BD*( 2) C 18 - C 25 | 0.07 | 0.01 | | 0.00 |
| ***. BD*( 2) C 469 - C 482 | /***. BD*( 2) C 22 - C 27 | 1.58 | 0.02 | | 0.01 |
| **total interaction between hydrogen bonded two mol1** | | **5.46** | **34.49** | | **0.29** |
| **Interaction between unit translated Molecule 2‘s** | | | | | |
| **from unit 7 to unit 8** | | | | | |
| 58. BD ( 1) O 51 - C 52 | /***. RY*( 1) H 145 | 0.36 | 2.00 | | 0.02 |
| 58. BD ( 1) O 51 - C 52 | /***. RY*( 2) H 145 | 0.06 | 3.45 | | 0.01 |
| 82. BD ( 1) C 71 - H 72 | /***. RY*( 2) H 167 | 0.09 | 2.47 | | 0.01 |
| 87. BD ( 2) C 75 - C 83 | /***. BD*( 2) C 162 - C 178 | 0.24 | 0.38 | | 0.01 |
| 88. BD ( 1) C 77 - H 78 | /***. RY*( 2) H 166 | 0.10 | 2.46 | | 0.01 |
| 862. LP ( 1) O 51 | /***. RY*( 1) H 145 | 0.15 | 1.62 | | 0.01 |
| 862. LP ( 1) O 51 | /***. RY*( 2) N 147 | 0.09 | 1.98 | | 0.01 |
| 862. LP ( 1) O 51 | /***. BD*( 1) H 142 - C 152 | 0.08 | 1.29 | | 0.01 |
| 862. LP ( 1) O 51 | /***. BD*( 1) H 145 - N 147 | 1.07 | 1.69 | | 0.04 |
| 862. LP ( 1) O 51 | /***. BD*( 1) N 147 - C 149 | 0.06 | 1.36 | | 0.01 |
| 863. LP ( 2) O 51 | /***. BD*( 1) H 142 - C 152 | 0.11 | 0.84 | | 0.01 |
| 863. LP ( 2) O 51 | /***. BD*( 1) H 145 - N 147 | 0.05 | 1.24 | | 0.01 |
| 865. LP ( 2) O 63 | /***. RY*( 1) H 155 | 0.09 | 1.17 | | 0.01 |
| 865. LP ( 2) O 63 | /***. BD*( 1) C 154 - H 155 | 0.06 | 1.07 | | 0.01 |
| **from unit 8 to unit 7** | | | | | |
| 161. BD ( 1) N 147 - C 150 | /***. BD*( 1) O 51 - C 52 | 0.05 | 1.45 | | 0.01 |
| 182. BD ( 2) C 162 - C 178 | /***. BD*( 2) C 75 - C 83 | 0.15 | 0.36 | | 0.01 |
| 184. BD ( 1) C 164 - H 166 | /***. RY*( 1) H 78 | 0.06 | 1.40 | | 0.01 |
| 185. BD ( 1) C 164 - H 167 | /***. RY*( 1) H 72 | 0.09 | 1.36 | | 0.01 |
| 195. BD ( 1) C 178 - H 179 | /***. RY*( 3) C 83 | 0.05 | 1.42 | | 0.01 |
| **total interaction between hydrogen bonded two mol2** | | **3.01** | **29.01** | | **0.23** |
| Interaction between Molecule 1 and 2 | | | | | |
| **from unit 6 to unit 5** | | | | | |
| 18. BD ( 1) O 15 - C 19 | /***. BD*( 1) O 63 - H 64 | 0.13 | 2.05 | | 0.01 |
| 31. BD ( 1) C 22 - C 27 | /***. RY*( 1) H 79 | 0.05 | 1.59 | | 0.01 |
| 857. LP ( 1) O 36 | /***. RY*( 4) O 63 | 0.09 | 2.13 | | 0.01 |
| 857. LP ( 1) O 36 | /***. RY*( 1) H 64 | 0.08 | 2.27 | | 0.01 |
| 857. LP ( 1) O 36 | /***. BD*( 1) C 56 - O 63 | 0.09 | 1.26 | | 0.01 |
| 857. LP ( 1) O 36 | /***. BD*( 1) O 63 - H 64 | 3.41 | 1.71 | | 0.07 |
| 858. LP ( 2) O 36 | /***. RY*( 4) O 63 | 0.33 | 1.70 | | 0.02 |
| 858. LP ( 2) O 36 | /***. RY*( 1) H 64 | 0.23 | 1.85 | | 0.02 |
| 858. LP ( 2) O 36 | /***. RY*( 4) H 64 | 0.19 | 2.43 | | 0.02 |
| 858. LP ( 2) O 36 | /***. BD*( 1) C 56 - O 63 | 0.19 | 0.83 | | 0.01 |
| 858. LP ( 2) O 36 | /***. BD*( 1) O 63 - H 64 | 4.76 | 1.29 | | 0.07 |
| **from unit 5 to unit 6** | | | | | |
| 67. BD ( 1) C 56 - O 63 | /***. BD*( 1) C 19 - O 36 | 0.07 | 1.78 | | 0.01 |
| 75. BD ( 1) O 63 - H 64 | /984. RY*( 1) H 5 | 0.08 | 1.76 | | 0.01 |
| 75. BD ( 1) O 63 - H 64 | /***. BD*( 1) C 19 - O 36 | 0.12 | 1.73 | | 0.01 |
| **total interaction between hydrogen bonded mol1 and 2** | | **9.82** | **24.38** | | **0.30** |
| **315K (Crystal 2)** | | | | | |
| Interaction between unit translated Molecule 1‘s | | | | | |
| **from unit 5 to unit 9** | | | | | |
| 152. BD ( 1) H 139 - C 149 | /***. BD*( 1) H 453 - C 470 | 0.11 | 1.07 | | 0.01 |
| 161. BD ( 1) O 147 - C 153 | /***. RY*( 1) H 452 | 0.22 | 1.94 | | 0.02 |
| 161. BD ( 1) O 147 - C 153 | /***. RY*( 2) H 452 | 0.11 | 3.41 | | 0.02 |
| 176. BD ( 1) C 156 - C 170 | /***. RY*( 1) C 472 | 0.05 | 2.31 | | 0.01 |
| 176. BD ( 1) C 156 - C 170 | /***. RY*( 3) C 472 | 0.10 | 1.54 | | 0.01 |
| 177. BD ( 2) C 156 - C 170 | /***. BD*( 2) C 466 - C 472 | 0.35 | 0.37 | | 0.01 |
| 177. BD ( 2) C 156 - C 170 | /***. BD*( 2) C 474 - C 484 | 0.07 | 0.37 | | 0.01 |
| 193. BD ( 1) C 170 - H 171 | /***. BD*( 2) C 474 - C 484 | 0.05 | 0.73 | | 0.01 |
| 195. BD ( 1) C 175 - H 176 | /***. RY*( 2) H 493 | 0.07 | 2.55 | | 0.01 |
| 197. BD ( 1) C 175 - H 178 | /***. RY*( 1) H 452 | 0.14 | 1.40 | | 0.01 |
| 882. LP ( 1) O 147 | /***. RY*( 1) H 452 | 0.10 | 1.59 | | 0.01 |
| 882. LP ( 1) O 147 | /***. RY*( 2) N 457 | 0.05 | 1.97 | | 0.01 |
| 882. LP ( 1) O 147 | /***. BD*( 1) H 452 - N 457 | 0.92 | 1.70 | | 0.04 |
| 882. LP ( 1) O 147 | /***. BD*( 1) H 453 - C 470 | 0.26 | 1.30 | | 0.02 |
| 882. LP ( 1) O 147 | /***. BD*( 1) N 457 - C 464 | 0.06 | 1.36 | | 0.01 |
| 883. LP ( 2) O 147 | /***. BD*( 1) H 452 - N 457 | 0.12 | 1.25 | | 0.01 |
| 883. LP ( 2) O 147 | /***. BD*( 1) H 453 - C 470 | 0.45 | 0.85 | | 0.02 |
| 884. LP ( 1) O 148 | /***. RY*( 1) H 452 | 0.05 | 1.42 | | 0.01 |
| **from unit 9 to unit 5** | | | | | |
| 500. BD ( 1) H 453 - C 470 | /***. BD*( 1) H 139 - C 149 | 0.11 | 1.06 | | 0.01 |
| 530. BD ( 1) C 472 - C 484 | /***. RY*( 1) C 170 | 0.06 | 2.36 | | 0.01 |
| 530. BD ( 1) C 472 - C 484 | /***. RY*( 3) C 170 | 0.09 | 1.51 | | 0.01 |
| 534. BD ( 2) C 474 - C 484 | /***. BD*( 2) C 156 - C 170 | 0.09 | 0.36 | | 0.01 |
| 544. BD ( 1) C 490 - H 492 | /***. RY*( 1) H 167 | 0.16 | 1.43 | | 0.01 |
| 544. BD ( 1) C 490 - H 492 | /***. RY*( 2) H 167 | 0.07 | 3.06 | | 0.01 |
| 545. BD ( 1) C 490 - H 493 | /***. RY*( 1) H 176 | 0.06 | 1.49 | | 0.01 |
| 545. BD ( 1) C 490 - H 493 | /***. RY*( 2) H 176 | 0.06 | 2.58 | | 0.01 |
| **Total interactions between hydrogen-bonded mol 1's** | | **3.98** | **40.98** | | **0.31** |
| Interaction between unit translated Molecule 2‘s | | | | | |
| **from unit 8 to unit 7** | | | | | |
| 252. BD ( 1) H 229 - C 247 | /***. BD*( 2) C 378 - O 383 | 0.09 | 0.62 | | 0.01 |
| 264. BD ( 1) N 238 - C 240 | /***. BD*( 1) O 372 - C 374 | 0.07 | 1.30 | | 0.01 |
| 278. BD ( 2) C 245 - C 263 | /***. BD*( 2) C 392 - C 400 | 0.14 | 0.35 | | 0.01 |
| 280. BD ( 1) C 249 - H 251 | /***. RY*( 1) H 395 | 0.06 | 1.39 | | 0.01 |
| 281. BD ( 1) C 249 - H 252 | /***. RY*( 1) H 389 | 0.09 | 1.37 | | 0.01 |
| 291. BD ( 1) C 263 - H 264 | /***. RY*( 3) C 400 | 0.05 | 1.45 | | 0.01 |
| **from unit 7 to unit 8** | | | | | |
| 407. BD ( 1) H 370 - O 381 | /***. RY*( 1) H 227 | 0.06 | 1.60 | | 0.01 |
| 410. BD ( 1) O 372 - C 374 | /***. RY*( 1) H 234 | 0.38 | 1.96 | | 0.02 |
| 431. BD ( 1) C 388 - H 389 | /***. RY*( 2) H 252 | 0.09 | 2.46 | | 0.01 |
| 436. BD ( 2) C 392 - C 400 | /***. BD*( 1) H 232 - C 242 | 0.08 | 0.80 | | 0.01 |
| 436. BD ( 2) C 392 - C 400 | /***. BD*( 2) C 245 - C 263 | 0.25 | 0.39 | | 0.01 |
| 437. BD ( 1) C 394 - H 395 | /***. RY*( 2) H 251 | 0.09 | 2.44 | | 0.01 |
| 442. BD ( 1) C 400 - H 401 | /***. BD*( 2) C 245 - C 263 | 0.05 | 0.74 | | 0.01 |
| 930. LP ( 1) O 372 | /***. RY*( 1) H 234 | 0.19 | 1.73 | | 0.02 |
| 930. LP ( 1) O 372 | /***. RY*( 2) N 238 | 0.10 | 1.91 | | 0.01 |
| 930. LP ( 1) O 372 | /***. BD*( 1) H 234 - N 238 | 1.27 | 1.70 | | 0.04 |
| 930. LP ( 1) O 372 | /***. BD*( 1) N 238 - C 240 | 0.06 | 1.21 | | 0.01 |
| 931. LP ( 2) O 372 | /***. BD*( 1) H 232 - C 242 | 0.06 | 0.84 | | 0.01 |
| 934. LP ( 2) O 381 | /***. RY*( 1) H 227 | 0.07 | 1.13 | | 0.01 |
| 934. LP ( 2) O 381 | /***. BD*( 1) H 227 - C 244 | 0.11 | 0.87 | | 0.01 |
| ***. BD*( 2) O 372 - C 374 | /***. RY*( 1) H 232 | 0.06 | 0.68 | | 0.01 |
| ***. BD*( 2) O 372 - C 374 | /***. RY*( 1) H 234 | 0.32 | 0.78 | | 0.04 |
| ***. BD*( 2) O 372 - C 374 | /***. RY*( 1) H 264 | 0.07 | 0.71 | | 0.02 |
| ***. BD*( 2) O 372 - C 374 | /***. BD*( 1) H 234 - N 238 | 0.15 | 0.75 | | 0.02 |
| ***. BD*( 2) C 392 - C 400 | /***. BD*( 2) C 245 - C 263 | 1.66 | 0.02 | | 0.01 |
| ***. BD*( 2) C 402 - C 404 | /***. BD*( 2) C 267 - C 269 | 0.11 | 0.01 | | 0.00 |
| **Total interactions between hydrogen-bonded mol 2's** | | **5.73** | **29.21** | | **0.33** |
| Interaction between Molecule 1 and 2 | | | | | |
| **from unit 6 to unit 5** | | | | | |
| 170. BD ( 1) O 154 - C 160 | /***. BD*( 1) H 370 - O 381 | 0.29 | 1.62 | | 0.02 |
| 181. BD ( 1) C 160 - O 165 | /***. BD*( 1) H 370 - O 381 | 0.10 | 1.78 | | 0.01 |
| 674. CR ( 1) O 165 | /***. BD*( 1) H 370 - O 381 | 0.10 | 19.86 | | 0.04 |
| 888. LP ( 1) O 165 | /***. RY*( 4) H 370 | 0.14 | 2.57 | | 0.02 |
| 888. LP ( 1) O 165 | /***. RY*( 5) H 370 | 0.08 | 3.16 | | 0.01 |
| 888. LP ( 1) O 165 | /***. BD*( 1) H 370 - O 381 | 7.89 | 1.29 | | 0.09 |
| 889. LP ( 2) O 165 | /***. RY*( 1) H 370 | 0.09 | 2.01 | | 0.01 |
| 889. LP ( 2) O 165 | /***. RY*( 5) H 370 | 0.15 | 2.75 | | 0.02 |
| 889. LP ( 2) O 165 | /***. RY*( 4) O 381 | 0.32 | 1.97 | | 0.02 |
| 889. LP ( 2) O 165 | /***. BD*( 1) H 370 - O 381 | 13.99 | 0.89 | | 0.10 |
| 889. LP ( 2) O 165 | /***. BD*( 1) C 378 - O 381 | 0.10 | 0.86 | | 0.01 |
| **from unit 5 to unit 6** | | | | | |
| 407. BD ( 1) H 370 - O 381 | /***. BD*( 1) C 160 - O 165 | 0.28 | 1.60 | | 0.02 |
| 933. LP ( 1) O 381 | /***. BD*( 1) C 160 - O 165 | 0.06 | 1.37 | | 0.01 |
| **total interaction between hydrogen bonded mol1 and 2** | | **23.59** | **41.73** | | **0.39** |
| **325K (Crystal 2)** | | | | | |
| **Interaction between unit translated Molecule 1‘s** | | | | | |
| **from unit 5 to unit 9** | | | | | |
| 3. BD ( 1) H 3 - C 8 | /***. BD*( 1) H 406 - C 420 | 0.08 | 1.06 | | 0.01 |
| 8. BD ( 1) O 7 - C 10 | /***. RY*( 1) H 407 | 0.20 | 1.95 | | 0.02 |
| 8. BD ( 1) O 7 - C 10 | /***. RY*( 2) H 407 | 0.07 | 3.37 | | 0.01 |
| 22. BD ( 1) C 16 - C 26 | /***. RY*( 1) C 423 | 0.05 | 2.32 | | 0.01 |
| 22. BD ( 1) C 16 - C 26 | /***. RY*( 2) C 423 | 0.11 | 1.92 | | 0.01 |
| 23. BD ( 2) C 16 - C 26 | /***. BD*( 2) C 419 - C 423 | 0.34 | 0.38 | | 0.01 |
| 23. BD ( 2) C 16 - C 26 | /***. BD*( 2) C 435 - C 437 | 0.07 | 0.38 | | 0.01 |
| 42. BD ( 1) C 34 - H 35 | /***. RY*( 2) H 449 | 0.07 | 2.55 | | 0.01 |
| 44. BD ( 1) C 34 - H 37 | /***. RY*( 1) H 407 | 0.14 | 1.44 | | 0.01 |
| 851. LP ( 1) O 7 | /***. RY*( 1) H 407 | 0.07 | 1.62 | | 0.01 |
| 851. LP ( 1) O 7 | /***. BD*( 1) H 406 - C 420 | 0.44 | 1.30 | | 0.02 |
| 851. LP ( 1) O 7 | /***. BD*( 1) H 407 - N 411 | 0.77 | 1.70 | | 0.03 |
| 851. LP ( 1) O 7 | /***. BD*( 1) N 411 - C 415 | 0.08 | 1.37 | | 0.01 |
| 852. LP ( 2) O 7 | /***. BD*( 1) H 406 - C 420 | 0.59 | 0.84 | | 0.02 |
| 852. LP ( 2) O 7 | /***. BD*( 1) H 407 - N 411 | 0.17 | 1.24 | | 0.01 |
| ***. BD*( 2) O 7 - C 10 | /***. RY*( 1) H 407 | 0.06 | 0.76 | | 0.01 |
| ***. BD*( 2) C 16 - C 26 | /***. BD*( 2) C 435 - C 437 | 1.02 | 0.02 | | 0.01 |
| **from unit 9 to unit 5** | | | | | |
| 448. BD ( 1) H 406 - C 420 | /***. BD*( 1) H 3 - C 8 | 0.11 | 1.06 | | 0.01 |
| 472. BD ( 1) C 423 - C 437 | /***. RY*( 1) C 26 | 0.06 | 2.34 | | 0.01 |
| 472. BD ( 1) C 423 - C 437 | /***. RY*( 3) C 26 | 0.09 | 1.48 | | 0.01 |
| 487. BD ( 2) C 435 - C 437 | /***. BD*( 2) C 16 - C 26 | 0.09 | 0.36 | | 0.01 |
| 495. BD ( 1) C 446 - H 448 | /***. RY*( 1) H 21 | 0.16 | 1.42 | | 0.01 |
| 495. BD ( 1) C 446 - H 448 | /***. RY*( 2) H 21 | 0.08 | 3.06 | | 0.01 |
| 496. BD ( 1) C 446 - H 449 | /***. RY*( 1) H 35 | 0.06 | 1.49 | | 0.01 |
| 496. BD ( 1) C 446 - H 449 | /***. RY*( 2) H 35 | 0.06 | 2.60 | | 0.01 |
| **Total interactions between hydrogen-bonded mol 1's** | | **5.04** | **38.03** | | **0.31** |
| Interaction between unit translated Molecule 2‘s | | | | | |
| **from unit 8 to unit 7** | | | | | |
| 197. BD ( 1) H 177 - C 191 | /***. BD*( 1) H 322 - C 331 | 0.08 | 1.04 | | 0.01 |
| 198. BD ( 1) H 178 - C 197 | /***. BD*( 2) C 333 - O 339 | 0.07 | 0.63 | | 0.01 |
| 232. BD ( 1) C 205 - H 207 | /***. RY*( 1) H 351 | 0.06 | 1.38 | | 0.01 |
| 233. BD ( 1) C 205 - H 208 | /***. RY*( 1) H 343 | 0.10 | 1.36 | | 0.01 |
| 237. BD ( 1) C 213 - H 214 | /***. RY*( 3) C 358 | 0.05 | 1.44 | | 0.01 |
| 237. BD ( 1) C 213 - H 214 | /***. BD*( 2) C 358 - C 360 | 0.05 | 0.71 | | 0.01 |
| 239. BD ( 2) C 213 - C 215 | /***. BD*( 2) C 358 - C 360 | 0.25 | 0.36 | | 0.01 |
|  |  | 0.66 | 6.92 | | 0.05 |
| **from unit 7 to unit 8** | | | | | |
| 360. BD ( 1) O 327 - C 329 | /***. RY*( 1) H 180 | 0.20 | 1.89 | | 0.02 |
| 361. BD ( 2) O 327 - C 329 | /***. BD*( 1) H 180 - N 187 | 0.10 | 1.35 | | 0.01 |
| 374. BD ( 1) O 334 - H 335 | /***. RY*( 1) H 183 | 0.07 | 1.76 | | 0.01 |
| 377. BD ( 2) C 337 - C 340 | /***. BD*( 1) H 177 - C 191 | 0.05 | 0.79 | | 0.01 |
| 380. BD ( 1) C 340 - C 358 | /***. RY*( 3) C 213 | 0.05 | 1.83 | | 0.01 |
| 381. BD ( 1) C 342 - H 343 | /***. RY*( 2) H 208 | 0.08 | 2.47 | | 0.01 |
| 387. BD ( 1) C 350 - H 351 | /***. RY*( 2) H 207 | 0.09 | 2.45 | | 0.01 |
| 397. BD ( 2) C 358 - C 360 | /***. BD*( 2) C 213 - C 215 | 0.10 | 0.39 | | 0.01 |
| 921. LP ( 1) O 327 | /***. RY*( 1) H 180 | 0.08 | 1.55 | | 0.01 |
| 921. LP ( 1) O 327 | /***. BD*( 1) H 177 - C 191 | 0.06 | 1.30 | | 0.01 |
| 921. LP ( 1) O 327 | /***. BD*( 1) H 180 - N 187 | 0.55 | 1.68 | | 0.03 |
| 921. LP ( 1) O 327 | /***. BD*( 1) N 187 - C 188 | 0.06 | 1.38 | | 0.01 |
| 922. LP ( 2) O 327 | /***. RY*( 1) H 178 | 0.06 | 1.10 | | 0.01 |
| 922. LP ( 2) O 327 | /***. BD*( 1) H 177 - C 191 | 0.09 | 0.84 | | 0.01 |
| 925. LP ( 2) O 334 | /***. BD*( 1) H 183 - C 195 | 0.16 | 0.90 | | 0.01 |
| ***. BD*( 2) O 327 - C 329 | /***. RY*( 1) H 180 | 0.10 | 0.71 | | 0.02 |
| ***. BD*( 2) O 327 - C 329 | /***. BD*( 1) H 180 - N 187 | 0.06 | 0.84 | | 0.02 |
| ***. BD*( 2) C 337 - C 340 | /***. BD*( 2) C 213 - C 215 | 0.18 | 0.01 | | 0.00 |
|  |  | 2.14 | 23.24 | | 0.20 |
| **Total interactions between hydrogen-bonded mol 2's** | | **2.80** | **30.16** | | **0.25** |
| Interaction between Molecule 1 and 2 | | | | | |
| **from unit 5 to unit 6** | | | | | |
| 217. BD ( 1) C 192 - O 193 | /***. BD*( 1) O 433 - C 434 | 0.06 | 1.82 | | 0.01 |
| 221. BD ( 1) O 193 - H 194 | /***. RY*( 1) H 445 | 0.11 | 1.79 | | 0.01 |
| 221. BD ( 1) O 193 - H 194 | /***. BD*( 1) O 433 - C 434 | 0.11 | 1.79 | | 0.01 |
| **from unit 6 to unit 5** | | | | | |
| 477. BD ( 1) O 429 - C 434 | /***. BD*( 1) O 193 - H 194 | 0.12 | 2.05 | | 0.01 |
| 486. BD ( 1) C 435 - C 437 | /***. RY*( 1) H 211 | 0.06 | 1.53 | | 0.01 |
| 944. LP ( 1) O 433 | /***. RY*( 4) O 193 | 0.07 | 2.13 | | 0.01 |
| 944. LP ( 1) O 433 | /***. RY*( 1) H 194 | 0.09 | 2.16 | | 0.01 |
| 944. LP ( 1) O 433 | /***. BD*( 1) C 192 - O 193 | 0.10 | 1.23 | | 0.01 |
| 944. LP ( 1) O 433 | /***. BD*( 1) O 193 - H 194 | 2.79 | 1.71 | | 0.06 |
| 945. LP ( 2) O 433 | /***. RY*( 4) O 193 | 0.24 | 1.71 | | 0.02 |
| 945. LP ( 2) O 433 | /***. RY*( 1) H 194 | 0.14 | 1.74 | | 0.02 |
| 945. LP ( 2) O 433 | /***. RY*( 4) H 194 | 0.16 | 2.37 | | 0.02 |
| 945. LP ( 2) O 433 | /***. BD*( 1) C 192 - O 193 | 0.19 | 0.81 | | 0.01 |
| 945. LP ( 2) O 433 | /***. BD*( 1) O 193 - H 194 | 3.84 | 1.29 | | 0.07 |
| **Total interactions between hydrogen-bonded mol 1 and mol 2** | | **8.08** | **24.13** | | **0.28** |
| **335K (Crystal 2)** | | | | | |
| **Interaction between unit translated Molecule 1‘s** | | | | | |
| **from unit 5 to unit 9** | | | | | |
| 306. BD ( 1) O 277 - C 280 | /***. RY*( 1) H 319 | 0.22 | 2.01 | 0.02 | |
| 306. BD ( 1) O 277 - C 280 | /***. RY*( 2) H 319 | 0.10 | 3.46 | 0.02 | |
| 316. BD ( 1) C 282 - C 301 | /***. RY*( 1) C 340 | 0.05 | 2.34 | 0.01 | |
| 316. BD ( 1) C 282 - C 301 | /***. RY*( 2) C 340 | 0.09 | 2.11 | 0.01 | |
| 317. BD ( 2) C 282 - C 301 | /***. BD*( 2) C 325 - C 340 | 0.33 | 0.38 | 0.01 | |
| 317. BD ( 2) C 282 - C 301 | /***. BD*( 2) C 338 - C 347 | 0.07 | 0.38 | 0.00 | |
| 342. BD ( 1) C 306 - H 307 | /***. RY*( 2) H 358 | 0.07 | 2.55 | 0.01 | |
| 344. BD ( 1) C 306 - H 309 | /***. RY*( 1) H 319 | 0.16 | 1.43 | 0.01 | |
| 911. LP ( 1) O 276 | /***. RY*( 1) H 319 | 0.06 | 1.45 | 0.01 | |
| 913. LP ( 1) O 277 | /***. RY*( 1) H 319 | 0.11 | 1.62 | 0.01 | |
| 913. LP ( 1) O 277 | /***. BD*( 1) N 318 - H 319 | 0.90 | 1.70 | 0.04 | |
| 913. LP ( 1) O 277 | /***. BD*( 1) N 318 - C 324 | 0.07 | 1.36 | 0.01 | |
| 914. LP ( 2) O 277 | /***. BD*( 1) N 318 - H 319 | 0.14 | 1.25 | 0.01 | |
| 914. LP ( 2) O 277 | /***. BD*( 1) C 328 - H 330 | 0.12 | 1.02 | 0.01 | |
| ***. BD*( 2) C 282 - C 301 | /***. BD*( 2) C 338 - C 347 | 0.84 | 0.03 | 0.01 | |
| **from unit 9 to unit 5** | | | | | |
| 379. BD ( 2) C 338 - C 347 | /***. BD*( 2) C 282 - C 301 | 0.09 | 0.36 | 0.01 | |
| 381. BD ( 1) C 340 - C 347 | /***. RY*( 1) C 301 | 0.06 | 2.34 | 0.01 | |
| 381. BD ( 1) C 340 - C 347 | /***. RY*( 3) C 301 | 0.09 | 1.47 | 0.01 | |
| 395. BD ( 1) C 355 - H 357 | /***. RY*( 1) H 291 | 0.19 | 1.40 | 0.02 | |
| 395. BD ( 1) C 355 - H 357 | /***. RY*( 2) H 291 | 0.08 | 3.05 | 0.01 | |
| 396. BD ( 1) C 355 - H 358 | /***. RY*( 1) H 307 | 0.05 | 1.52 | 0.01 | |
| 396. BD ( 1) C 355 - H 358 | /***. RY*( 2) H 307 | 0.07 | 2.55 | 0.01 | |
| **Total interactions between hydrogen-bonded mol 1's** | | **3.96** | **35.78** | **0.27** | |
| **Interaction between unit translated Molecule 2‘s** | | | | | |
| **from unit 7 to unit 8** | | | | | |
| 107. BD ( 1) O 99 - C 102 | /***. RY*( 1) H 145 | 0.20 | 1.91 | 0.02 | |
| 108. BD ( 2) O 99 - C 102 | /***. BD*( 1) N 144 - H 145 | 0.09 | 1.38 | 0.01 | |
| 132. BD ( 1) C 116 - H 117 | /***. RY*( 2) H 175 | 0.08 | 2.47 | 0.01 | |
| 135. BD ( 1) C 120 - H 121 | /***. RY*( 2) H 174 | 0.09 | 2.46 | 0.01 | |
| 151. BD ( 2) C 136 - C 140 | /***. BD*( 2) C 178 - C 182 | 0.09 | 0.40 | 0.01 | |
| 876. LP ( 1) O 99 | /***. RY*( 1) H 145 | 0.08 | 1.56 | 0.01 | |
| 876. LP ( 1) O 99 | /***. BD*( 1) N 144 - H 145 | 0.54 | 1.69 | 0.03 | |
| 876. LP ( 1) O 99 | /***. BD*( 1) N 144 - C 146 | 0.06 | 1.39 | 0.01 | |
| 877. LP ( 2) O 99 | /***. RY*( 1) H 155 | 0.06 | 1.14 | 0.01 | |
| 880. LP ( 2) O 113 | /***. BD*( 1) C 168 - H 169 | 0.05 | 1.09 | 0.01 | |
| ***. BD*( 2) O 99 - C 102 | /***. RY*( 1) H 145 | 0.14 | 0.71 | 0.02 | |
| ***. BD*( 2) O 99 - C 102 | /***. BD*( 1) N 144 - H 145 | 0.07 | 0.84 | 0.02 | |
| ***. BD*( 2) C 115 - C 132 | /***. BD*( 2) C 178 - C 182 | 0.10 | 0.02 | 0.00 | |
| ***. BD*( 2) C 136 - C 140 | /***. BD*( 2) C 178 - C 182 | 2.07 | 0.02 | 0.01 | |
| **from unit 8 to unit 7** | | | | | |
| 190. BD ( 1) C 172 - H 174 | /***. RY*( 1) H 121 | 0.06 | 1.40 | 0.01 | |
| 191. BD ( 1) C 172 - H 175 | /***. RY*( 1) H 117 | 0.10 | 1.36 | 0.01 | |
| 196. BD ( 2) C 178 - C 182 | /***. BD*( 2) C 136 - C 140 | 0.24 | 0.36 | 0.01 | |
| 201. BD ( 1) C 182 - H 183 | /***. BD*( 2) C 136 - C 140 | 0.05 | 0.71 | 0.01 | |
| **Total interactions between hydrogen-bonded mol 2's** | | **4.17** | **20.91** | **0.20** | |
| **Interaction between Molecule 1 and 2** | | | | | |
| **from unit 5 to unit 6** | | | | | |
| 124. BD ( 1) C 112 - O 113 | /***. BD*( 1) C 349 - O 354 | 0.06 | 1.89 | 0.01 | |
| 128. BD ( 1) O 113 - H 114 | /***. RY*( 1) H 361 | 0.12 | 1.81 | 0.01 | |
| 128. BD ( 1) O 113 - H 114 | /***. BD*( 1) C 349 - O 354 | 0.10 | 1.84 | 0.01 | |
| **from unit 6 to unit 5** | | | | | |
| 385. BD ( 1) O 343 - C 349 | /***. BD*( 1) O 113 - H 114 | 0.12 | 1.96 | 0.01 | |
| 926. LP ( 1) O 354 | /***. RY*( 4) O 113 | 0.08 | 2.12 | 0.01 | |
| 926. LP ( 1) O 354 | /***. RY*( 1) H 114 | 0.09 | 2.07 | 0.01 | |
| 926. LP ( 1) O 354 | /***. BD*( 1) C 112 - O 113 | 0.10 | 1.23 | 0.01 | |
| 926. LP ( 1) O 354 | /***. BD*( 1) O 113 - H 114 | 2.72 | 1.68 | 0.06 | |
| 927. LP ( 2) O 354 | /***. RY*( 4) O 113 | 0.22 | 1.71 | 0.02 | |
| 927. LP ( 2) O 354 | /***. RY*( 1) H 114 | 0.10 | 1.66 | 0.01 | |
| 927. LP ( 2) O 354 | /***. RY*( 4) H 114 | 0.13 | 2.46 | 0.02 | |
| 927. LP ( 2) O 354 | /***. BD*( 1) C 112 - O 113 | 0.18 | 0.81 | 0.01 | |
| 927. LP ( 2) O 354 | /***. BD*( 1) O 113 - H 114 | 3.17 | 1.27 | 0.06 | |
| **Total interactions between hydrogen-bonded mol 1 and mol 2** | | **7.19** | **22.51** | **0.26** | |
| **337K (Crystal 3)** | | | | | |
| **Interaction between unit translated Molecule 1‘s** | | | | | |
| **from unit 5 to unit 9** | | | | | |
| 206. BD ( 1) C 186 - O 187 | /***. RY*( 1) H 363 | 0.21 | 2.00 | 0.02 | |
| 206. BD ( 1) C 186 - O 187 | /***. RY*( 2) H 363 | 0.09 | 3.44 | 0.02 | |
| 216. BD ( 1) C 191 - C 203 | /***. RY*( 1) C 377 | 0.05 | 2.36 | 0.01 | |
| 216. BD ( 1) C 191 - C 203 | /***. RY*( 2) C 377 | 0.11 | 1.94 | 0.01 | |
| 217. BD ( 2) C 191 - C 203 | /***. BD*( 2) C 369 - C 377 | 0.33 | 0.38 | 0.01 | |
| 217. BD ( 2) C 191 - C 203 | /***. BD*( 2) C 375 - C 392 | 0.06 | 0.39 | 0.00 | |
| 242. BD ( 1) C 215 - H 217 | /***. RY*( 2) H 401 | 0.06 | 2.56 | 0.01 | |
| 243. BD ( 1) C 215 - H 218 | /***. RY*( 1) H 363 | 0.16 | 1.44 | 0.01 | |
| 891. LP ( 1) O 185 | /***. RY*( 1) H 363 | 0.06 | 1.46 | 0.01 | |
| 893. LP ( 1) O 187 | /***. RY*( 1) H 363 | 0.11 | 1.62 | 0.01 | |
| 893. LP ( 1) O 187 | /***. BD*( 1) N 362 - H 363 | 0.85 | 1.70 | 0.03 | |
| 893. LP ( 1) O 187 | /***. BD*( 1) N 362 - C 365 | 0.07 | 1.37 | 0.01 | |
| 894. LP ( 2) O 187 | /***. BD*( 1) N 362 - H 363 | 0.16 | 1.25 | 0.01 | |
| 894. LP ( 2) O 187 | /***. BD*( 1) C 372 - H 374 | 0.12 | 1.02 | 0.01 | |
| ***. BD*( 2) C 191 - C 203 | /***. BD*( 2) C 375 - C 392 | 0.85 | 0.03 | 0.01 | |
| ***. BD*( 2) C 196 - C 213 | /***. BD*( 2) C 369 - C 377 | 0.05 | 0.01 | 0.00 | |
| **from unit 9 to unit 5** | | | | | |
| 421. BD ( 2) C 375 - C 392 | /***. BD*( 2) C 191 - C 203 | 0.09 | 0.35 | 0.01 | |
| 423. BD ( 1) C 377 - C 392 | /***. RY*( 1) C 203 | 0.06 | 2.36 | 0.01 | |
| 423. BD ( 1) C 377 - C 392 | /***. RY*( 3) C 203 | 0.09 | 1.48 | 0.01 | |
| 442. BD ( 1) C 398 - H 399 | /***. RY*( 1) H 209 | 0.14 | 1.37 | 0.01 | |
| 442. BD ( 1) C 398 - H 399 | /***. RY*( 2) H 209 | 0.07 | 3.06 | 0.01 | |
| 444. BD ( 1) C 398 - H 401 | /***. RY*( 1) H 217 | 0.06 | 1.49 | 0.01 | |
| 444. BD ( 1) C 398 - H 401 | /***. RY*( 2) H 217 | 0.06 | 2.56 | 0.01 | |
| 928. LP ( 1) N 362 | /***. RY*( 1) H 208 | 0.06 | 1.06 | 0.01 | |
| **Total interactions between hydrogen-bonded mol 1's** | | **3.97** | **36.70** | **0.27** | |
| Interaction between unit translated Molecule 2‘s | | | | | |
| **from unit 7 to unit 8** | | | | | |
| 57. BD ( 1) C 52 - O 55 | /***. RY*( 1) H 321 | 0.18 | 1.90 | 0.02 | |
| 58. BD ( 2) C 52 - O 55 | /***. BD*( 1) N 320 - H 321 | 0.11 | 1.38 | 0.01 | |
| 82. BD ( 1) O 70 - H 71 | /***. RY*( 1) H 330 | 0.05 | 1.81 | 0.01 | |
| 87. BD ( 1) C 77 - H 79 | /***. RY*( 2) H 341 | 0.07 | 2.45 | 0.01 | |
| 92. BD ( 1) C 83 - H 85 | /***. RY*( 2) H 343 | 0.07 | 2.47 | 0.01 | |
| 101. BD ( 2) C 91 - C 93 | /***. BD*( 2) C 354 - C 356 | 0.09 | 0.40 | 0.01 | |
| 866. LP ( 1) O 55 | /***. RY*( 1) H 321 | 0.09 | 1.57 | 0.01 | |
| 866. LP ( 1) O 55 | /***. BD*( 1) C 319 - N 320 | 0.06 | 1.39 | 0.01 | |
| 866. LP ( 1) O 55 | /***. BD*( 1) N 320 - H 321 | 0.53 | 1.69 | 0.03 | |
| 867. LP ( 2) O 55 | /***. RY*( 1) H 334 | 0.06 | 1.13 | 0.01 | |
| 869. LP ( 2) O 70 | /***. BD*( 1) C 329 - H 330 | 0.05 | 1.11 | 0.01 | |
| ***. BD*( 2) C 52 - O 55 | /***. RY*( 1) H 321 | 0.16 | 0.71 | 0.02 | |
| ***. BD*( 2) C 52 - O 55 | /***. BD*( 1) N 320 - H 321 | 0.08 | 0.83 | 0.02 | |
| ***. BD*( 2) C 69 - C 81 | /***. BD*( 2) C 354 - C 356 | 0.16 | 0.02 | 0.00 | |
| **from unit 8 to unit 7** | | | | | |
| 379. BD ( 1) C 340 - H 341 | /***. RY*( 1) H 79 | 0.06 | 1.33 | 0.01 | |
| 381. BD ( 1) C 340 - H 343 | /***. RY*( 1) H 85 | 0.09 | 1.31 | 0.01 | |
| 390. BD ( 1) C 354 - H 355 | /***. RY*( 3) C 91 | 0.05 | 1.44 | 0.01 | |
| 390. BD ( 1) C 354 - H 355 | /***. BD*( 2) C 91 - C 93 | 0.06 | 0.72 | 0.01 | |
| 392. BD ( 2) C 354 - C 356 | /***. BD*( 2) C 91 - C 93 | 0.26 | 0.37 | 0.01 | |
| **Total interactions between hydrogen-bonded mol 2's** | | **2.28** | **24.03** | **0.21** | |
| Interaction between Molecule 1 and 2 | | | | | |
| **from unit 5 to unit 6** | | | | | |
| 76. BD ( 1) C 68 - O 70 | /***. BD*( 1) O 212 - C 223 | 0.06 | 1.91 | 0.01 | |
| 82. BD ( 1) O 70 - H 71 | /***. RY*( 1) H 226 | 0.08 | 1.66 | 0.01 | |
| 82. BD ( 1) O 70 - H 71 | /***. BD*( 1) O 212 - C 223 | 0.09 | 1.84 | 0.01 | |
| 88. BD ( 1) C 77 - H 80 | /***. RY*( 1) H 214 | 0.06 | 1.43 | 0.01 | |
| 88. BD ( 1) C 77 - H 80 | /***. RY*( 2) H 214 | 0.06 | 2.94 | 0.01 | |
| 868. LP ( 1) O 70 | /***. RY*( 1) H 226 | 0.06 | 1.30 | 0.01 | |
| **from unit 6 to unit 5** | | | | | |
| 231. BD ( 1) O 205 - C 223 | /***. BD*( 1) O 70 - H 71 | 0.10 | 1.94 | 0.01 | |
| 239. BD ( 2) O 212 - C 223 | /***. BD*( 1) O 70 - H 71 | 0.06 | 1.39 | 0.01 | |
| 897. LP ( 1) O 212 | /***. RY*( 4) O 70 | 0.05 | 2.05 | 0.01 | |
| 897. LP ( 1) O 212 | /***. RY*( 1) H 71 | 0.06 | 1.98 | 0.01 | |
| 897. LP ( 1) O 212 | /***. BD*( 1) C 68 - O 70 | 0.10 | 1.25 | 0.01 | |
| 897. LP ( 1) O 212 | /***. BD*( 1) O 70 - H 71 | 2.02 | 1.67 | 0.05 | |
| 898. LP ( 2) O 212 | /***. RY*( 4) O 70 | 0.16 | 1.63 | 0.02 | |
| 898. LP ( 2) O 212 | /***. RY*( 1) H 71 | 0.05 | 1.56 | 0.01 | |
| 898. LP ( 2) O 212 | /***. RY*( 4) H 71 | 0.12 | 2.30 | 0.02 | |
| 898. LP ( 2) O 212 | /***. BD*( 1) C 68 - O 70 | 0.16 | 0.84 | 0.01 | |
| 898. LP ( 2) O 212 | /***. BD*( 1) O 70 - H 71 | 2.63 | 1.26 | 0.05 | |
| **Total interactions between hydrogen-bonded mol 1 and mol 2** | | **5.92** | **28.95** | **0.26** | |
| **339K (Crystal 3)** | | | | | |
| **Interaction between unit translated Molecule 1‘s** | | | | | |
| **from unit 5 to unit 9** | | | | | |
| 252. BD ( 1) H 230 - C 238 | /***. BD*( 1) H 409 - C 421 | 0.22 | 1.05 | 0.01 | |
| 259. BD ( 1) O 236 - C 239 | /***. RY*( 1) H 407 | 0.16 | 1.91 | 0.02 | |
| 260. BD ( 2) O 236 - C 239 | /***. BD*( 1) H 407 - N 411 | 0.09 | 1.43 | 0.01 | |
| 272. BD ( 2) C 241 - C 258 | /***. BD*( 2) C 417 - C 432 | 0.10 | 0.39 | 0.01 | |
| 274. BD ( 1) C 243 - C 258 | /***. RY*( 1) C 426 | 0.05 | 2.34 | 0.01 | |
| 274. BD ( 1) C 243 - C 258 | /***. RY*( 2) C 426 | 0.11 | 1.92 | 0.01 | |
| 288. BD ( 1) C 258 - H 259 | /***. BD*( 2) C 417 - C 432 | 0.05 | 0.75 | 0.01 | |
| 293. BD ( 1) C 263 - H 264 | /***. RY*( 2) H 446 | 0.06 | 2.54 | 0.01 | |
| 295. BD ( 1) C 263 - H 266 | /***. RY*( 1) H 407 | 0.17 | 1.40 | 0.01 | |
| 902. LP ( 1) O 236 | /***. RY*( 1) H 407 | 0.09 | 1.59 | 0.01 | |
| 902. LP ( 1) O 236 | /***. BD*( 1) H 407 - N 411 | 0.64 | 1.72 | 0.03 | |
| 902. LP ( 1) O 236 | /***. BD*( 1) H 409 - C 421 | 0.11 | 1.28 | 0.01 | |
| 902. LP ( 1) O 236 | /***. BD*( 1) N 411 - C 415 | 0.06 | 1.37 | 0.01 | |
| 902. LP ( 1) O 236 | /***. BD*( 1) C 416 - C 421 | 0.05 | 1.29 | 0.01 | |
| 903. LP ( 2) O 236 | /***. BD*( 1) H 407 - N 411 | 0.11 | 1.26 | 0.01 | |
| 903. LP ( 2) O 236 | /***. BD*( 1) H 409 - C 421 | 0.25 | 0.83 | 0.01 | |
| 904. LP ( 1) O 237 | /***. RY*( 1) H 407 | 0.05 | 1.42 | 0.01 | |
| ***. BD*( 2) O 236 - C 239 | /***. RY*( 1) H 407 | 0.15 | 0.72 | 0.02 | |
| ***. BD*( 2) O 236 - C 239 | /***. BD*( 1) H 407 - N 411 | 0.09 | 0.85 | 0.02 | |
| ***. BD*( 2) C 240 - C 243 | /***. BD*( 2) C 416 - C 426 | 1.34 | 0.01 | 0.01 | |
| ***. BD*( 2) C 240 - C 243 | /***. BD*( 2) C 417 - C 432 | 0.05 | 0.02 | 0.00 | |
| ***. BD*( 2) C 241 - C 258 | /***. BD*( 2) C 416 - C 426 | 1.35 | 0.01 | 0.01 | |
| ***. BD*( 2) C 241 - C 258 | /***. BD*( 2) C 417 - C 432 | 0.89 | 0.02 | 0.01 | |
| **from unit 9 to unit 5** | | | | | |
| 451. BD ( 1) H 409 - C 421 | /***. BD*( 1) H 230 - C 238 | 0.29 | 1.04 | 0.02 | |
| 464. BD ( 2) C 416 - C 426 | /***. BD*( 2) C 240 - C 243 | 0.11 | 0.36 | 0.01 | |
| 477. BD ( 1) C 426 - C 432 | /***. RY*( 1) C 258 | 0.06 | 2.36 | 0.01 | |
| 477. BD ( 1) C 426 - C 432 | /***. RY*( 3) C 258 | 0.09 | 1.48 | 0.01 | |
| 493. BD ( 1) C 443 - H 445 | /***. RY*( 1) H 247 | 0.18 | 1.41 | 0.01 | |
| 493. BD ( 1) C 443 - H 445 | /***. RY*( 2) H 247 | 0.08 | 3.05 | 0.01 | |
| 494. BD ( 1) C 443 - H 446 | /***. RY*( 1) H 264 | 0.06 | 1.50 | 0.01 | |
| 494. BD ( 1) C 443 - H 446 | /***. RY*( 2) H 264 | 0.06 | 2.57 | 0.01 | |
| 937. LP ( 1) N 411 | /***. RY*( 1) H 248 | 0.05 | 1.08 | 0.01 | |
| **Total interactions between hydrogen-bonded mol 1's** | | **7.22** | **40.97** | **0.36** | |
| Interaction between unit translated Molecule 2‘s | | | | | |
| **from unit 7 to unit 8** | | | | | |
| 360. BD ( 1) O 327 - C 328 | /***. RY*( 1) H 374 | 0.20 | 1.93 | 0.02 | |
| 361. BD ( 2) O 327 - C 328 | /***. BD*( 1) N 373 - H 374 | 0.09 | 1.38 | 0.01 | |
| 377. BD ( 2) C 338 - C 343 | /***. BD*( 1) H 365 - C 377 | 0.09 | 0.78 | 0.01 | |
| 379. BD ( 1) C 339 - H 340 | /***. RY*( 2) H 395 | 0.10 | 2.45 | 0.01 | |
| 384. BD ( 1) C 345 - H 346 | /***. RY*( 2) H 396 | 0.08 | 2.47 | 0.01 | |
| 395. BD ( 2) C 356 - C 358 | /***. BD*( 2) C 398 - C 404 | 0.09 | 0.40 | 0.01 | |
| 921. LP ( 1) O 327 | /***. RY*( 1) H 374 | 0.09 | 1.55 | 0.01 | |
| 921. LP ( 1) O 327 | /***. BD*( 1) C 372 - N 373 | 0.06 | 1.37 | 0.01 | |
| 921. LP ( 1) O 327 | /***. BD*( 1) N 373 - H 374 | 0.53 | 1.69 | 0.03 | |
| 922. LP ( 2) O 327 | /***. RY*( 1) H 364 | 0.05 | 1.11 | 0.01 | |
| 922. LP ( 2) O 327 | /***. BD*( 1) H 364 - C 379 | 0.08 | 0.82 | 0.01 | |
| 922. LP ( 2) O 327 | /***. BD*( 1) H 365 - C 377 | 0.06 | 0.83 | 0.01 | |
| 925. LP ( 2) O 337 | /***. BD*( 1) H 368 - C 380 | 0.05 | 0.90 | 0.01 | |
| ***. BD*( 2) O 327 - C 328 | /***. RY*( 1) H 374 | 0.12 | 0.70 | 0.02 | |
| ***. BD*( 2) O 327 - C 328 | /***. BD*( 1) N 373 - H 374 | 0.06 | 0.84 | 0.02 | |
| ***. BD*( 2) C 338 - C 343 | /***. BD*( 1) H 365 - C 377 | 0.06 | 0.42 | 0.01 | |
| ***. BD*( 2) C 338 - C 343 | /***. BD*( 2) C 398 - C 404 | 0.11 | 0.02 | 0.00 | |
| ***. BD*( 2) C 356 - C 358 | /***. BD*( 2) C 398 - C 404 | 2.94 | 0.01 | 0.01 | |
| **from unit 8 to unit 7** | | | | | |
| 401. BD ( 1) H 364 - C 379 | /***. BD*( 2) C 334 - O 353 | 0.07 | 0.59 | 0.01 | |
| 402. BD ( 1) H 365 - C 377 | /***. BD*( 1) H 318 - C 332 | 0.05 | 1.04 | 0.01 | |
| 437. BD ( 1) C 393 - H 395 | /***. RY*( 1) H 340 | 0.07 | 1.39 | 0.01 | |
| 438. BD ( 1) C 393 - H 396 | /***. RY*( 1) H 346 | 0.09 | 1.35 | 0.01 | |
| 439. BD ( 1) C 398 - H 399 | /***. BD*( 2) C 356 - C 358 | 0.06 | 0.71 | 0.01 | |
| 441. BD ( 2) C 398 - C 404 | /***. BD*( 2) C 356 - C 358 | 0.25 | 0.37 | 0.01 | |
| **Total interactions between hydrogen-bonded mol 2's** | | **5.45** | **25.12** | **0.24** | |
| Interaction between Molecule 1 and 2 | | | | | |
| **from unit 5 to unit 6** | | | | | |
| 406. BD ( 1) H 369 - O 381 | /***. RY*( 1) O 438 | 0.12 | 2.10 | 0.01 | |
| 406. BD ( 1) H 369 - O 381 | /***. RY*( 1) H 449 | 0.13 | 1.62 | 0.01 | |
| 406. BD ( 1) H 369 - O 381 | /***. BD*( 1) C 437 - O 438 | 0.24 | 1.65 | 0.02 | |
| 421. BD ( 1) C 378 - O 381 | /***. RY*( 1) H 449 | 0.05 | 1.88 | 0.01 | |
| **from unit 6 to unit 5** | | | | | |
| 466. BD ( 1) C 417 - C 432 | /***. RY*( 1) H 385 | 0.06 | 1.54 | 0.01 | |
| 485. BD ( 1) O 436 - C 437 | /***. BD*( 1) H 369 - O 381 | 0.15 | 1.52 | 0.01 | |
| 487. BD ( 2) C 437 - O 438 | /***. BD*( 1) H 369 - O 381 | 0.17 | 0.96 | 0.01 | |
| 944. LP ( 1) O 438 | /***. RY*( 2) H 369 | 0.06 | 2.58 | 0.01 | |
| 944. LP ( 1) O 438 | /***. BD*( 1) H 369 - O 381 | 5.06 | 1.24 | 0.07 | |
| 944. LP ( 1) O 438 | /***. BD*( 1) C 378 - O 381 | 0.07 | 1.27 | 0.01 | |
| 945. LP ( 2) O 438 | /***. RY*( 4) O 381 | 0.10 | 1.51 | 0.01 | |
| 945. LP ( 2) O 438 | /***. BD*( 1) H 369 - O 381 | 3.56 | 0.83 | 0.05 | |
| 945. LP ( 2) O 438 | /***. BD*( 1) C 378 - O 381 | 0.15 | 0.86 | 0.01 | |
| ***. BD*( 2) C 437 - O 438 | /***. BD*( 1) H 369 - O 381 | 0.08 | 0.37 | 0.02 | |
| **Total interactions between hydrogen-bonded mol 1 and mol 2** | | **10.00** | **19.93** | **0.27** | |
| **340K (Crystal 3)** | | | | | |
| Interaction between unit translated Molecule 1‘s | | | | | |
| **from unit 5 to unit 9** | | | | | |
| 99. BD ( 1) H 89 - C 96 | /***. BD*( 1) C 331 - H 333 | 0.08 | 1.23 | 0.01 | |
| 106. BD ( 1) O 94 - C 95 | /***. RY*( 1) H 317 | 0.11 | 1.88 | 0.01 | |
| 107. BD ( 2) O 94 - C 95 | /***. BD*( 1) H 317 - N 319 | 0.10 | 1.45 | 0.01 | |
| 115. BD ( 1) C 98 - C 115 | /***. RY*( 1) C 329 | 0.06 | 2.36 | 0.01 | |
| 115. BD ( 1) C 98 - C 115 | /***. RY*( 2) C 329 | 0.09 | 2.07 | 0.01 | |
| 116. BD ( 2) C 98 - C 115 | /***. BD*( 2) C 323 - C 329 | 0.33 | 0.38 | 0.01 | |
| 116. BD ( 2) C 98 - C 115 | /***. BD*( 2) C 327 - C 344 | 0.06 | 0.39 | 0.00 | |
| 142. BD ( 1) C 125 - H 126 | /***. RY*( 2) H 349 | 0.06 | 2.55 | 0.01 | |
| 144. BD ( 1) C 125 - H 128 | /***. RY*( 1) H 317 | 0.18 | 1.39 | 0.01 | |
| 871. LP ( 1) O 94 | /***. RY*( 1) H 317 | 0.07 | 1.58 | 0.01 | |
| 871. LP ( 1) O 94 | /***. BD*( 1) H 317 - N 319 | 0.52 | 1.70 | 0.03 | |
| 871. LP ( 1) O 94 | /***. BD*( 1) N 319 - C 321 | 0.06 | 1.36 | 0.01 | |
| 872. LP ( 2) O 94 | /***. BD*( 1) H 317 - N 319 | 0.09 | 1.24 | 0.01 | |
| 872. LP ( 2) O 94 | /***. BD*( 1) C 331 - H 333 | 0.11 | 1.02 | 0.01 | |
| ***. BD*( 2) O 94 - C 95 | /***. RY*( 1) H 317 | 0.23 | 0.66 | 0.03 | |
| ***. BD*( 2) O 94 - C 95 | /***. RY*( 1) H 333 | 0.07 | 0.87 | 0.02 | |
| ***. BD*( 2) O 94 - C 95 | /***. BD*( 1) H 317 - N 319 | 0.13 | 0.78 | 0.02 | |
| ***. BD*( 2) C 98 - C 115 | /***. BD*( 2) C 327 - C 344 | 0.89 | 0.03 | 0.01 | |
| ***. BD*( 2) C 101 - C 118 | /***. BD*( 2) C 323 - C 329 | 0.07 | 0.01 | 0.00 | |
| **from unit 9 to unit 5** | | | | | |
| 372. BD ( 2) C 327 - C 344 | /***. BD*( 2) C 98 - C 115 | 0.09 | 0.36 | 0.01 | |
| 374. BD ( 1) C 329 - C 344 | /***. RY*( 1) C 115 | 0.06 | 2.37 | 0.01 | |
| 374. BD ( 1) C 329 - C 344 | /***. RY*( 3) C 115 | 0.09 | 1.49 | 0.01 | |
| 376. BD ( 1) C 331 - H 333 | /***. BD*( 1) H 89 - C 96 | 0.14 | 1.09 | 0.01 | |
| 387. BD ( 1) C 346 - H 348 | /***. RY*( 1) H 109 | 0.19 | 1.41 | 0.02 | |
| 387. BD ( 1) C 346 - H 348 | /***. RY*( 2) H 109 | 0.08 | 3.04 | 0.01 | |
| 388. BD ( 1) C 346 - H 349 | /***. RY*( 1) H 126 | 0.06 | 1.51 | 0.01 | |
| 388. BD ( 1) C 346 - H 349 | /***. RY*( 2) H 126 | 0.06 | 2.56 | 0.01 | |
| 919. LP ( 1) N 319 | /***. RY*( 1) H 110 | 0.06 | 1.08 | 0.01 | |
| **Total interactions between hydrogen-bonded mol 1's** | | **4.14** | **37.86** | **0.33** | |
| Interaction between unit translated Molecule 2‘s | | | | | |
| **from unit 7 to unit 8** | | | | | |
| 9. BD ( 1) H 9 - O 31 | /***. RY*( 1) H 411 | 0.05 | 1.60 | 0.01 | |
| 12. BD ( 1) O 11 - C 12 | /***. RY*( 1) H 409 | 0.40 | 2.02 | 0.03 | |
| 12. BD ( 1) O 11 - C 12 | /***. RY*( 2) H 409 | 0.07 | 3.50 | 0.01 | |
| 28. BD ( 2) C 19 - C 29 | /***. BD*( 1) H 413 - C 421 | 0.08 | 0.79 | 0.01 | |
| 30. BD ( 1) C 21 - H 22 | /***. RY*( 2) H 440 | 0.08 | 2.47 | 0.01 | |
| 33. BD ( 1) C 25 - H 26 | /***. RY*( 2) H 439 | 0.09 | 2.46 | 0.01 | |
| 48. BD ( 2) C 41 - C 43 | /***. BD*( 2) C 441 - C 443 | 0.08 | 0.41 | 0.01 | |
| 852. LP ( 1) O 11 | /***. RY*( 1) H 409 | 0.18 | 1.65 | 0.02 | |
| 852. LP ( 1) O 11 | /***. RY*( 2) N 419 | 0.09 | 1.96 | 0.01 | |
| 852. LP ( 1) O 11 | /***. BD*( 1) H 406 - C 422 | 0.05 | 1.29 | 0.01 | |
| 852. LP ( 1) O 11 | /***. BD*( 1) H 409 - N 419 | 1.25 | 1.66 | 0.04 | |
| 852. LP ( 1) O 11 | /***. BD*( 1) H 413 - C 421 | 0.06 | 1.29 | 0.01 | |
| 853. LP ( 2) O 11 | /***. RY*( 1) H 406 | 0.05 | 1.16 | 0.01 | |
| 853. LP ( 2) O 11 | /***. BD*( 1) H 406 - C 422 | 0.11 | 0.84 | 0.01 | |
| 853. LP ( 2) O 11 | /***. BD*( 1) H 413 - C 421 | 0.08 | 0.83 | 0.01 | |
| 856. LP ( 2) O 31 | /***. BD*( 1) H 411 - C 425 | 0.07 | 0.88 | 0.01 | |
| ***. BD*( 2) C 19 - C 29 | /***. BD*( 2) C 441 - C 443 | 0.13 | 0.02 | 0.00 | |
| **from unit 8 to unit 7** | | | | | |
| 448. BD ( 1) H 406 - C 422 | /***. BD*( 2) C 18 - O 40 | 0.07 | 0.60 | 0.01 | |
| 455. BD ( 1) H 413 - C 421 | /***. BD*( 1) H 5 - C 15 | 0.07 | 1.04 | 0.01 | |
| 465. BD ( 1) N 419 - C 420 | /***. BD*( 1) O 11 - C 12 | 0.06 | 1.41 | 0.01 | |
| 486. BD ( 1) C 437 - H 439 | /***. RY*( 1) H 26 | 0.07 | 1.40 | 0.01 | |
| 487. BD ( 1) C 437 - H 440 | /***. RY*( 1) H 22 | 0.10 | 1.33 | 0.01 | |
| 488. BD ( 1) C 441 - H 442 | /***. BD*( 2) C 41 - C 43 | 0.05 | 0.72 | 0.01 | |
| 490. BD ( 2) C 441 - C 443 | /***. BD*( 2) C 41 - C 43 | 0.25 | 0.37 | 0.01 | |
| **Total interactions between hydrogen-bonded mol 2's** | | **3.59** | **31.70** | **0.26** | |
| Interaction between Molecule 1 and 2 | | | | | |
| **from unit 5 to unit 6** | | | | | |
| 9. BD ( 1) H 9 - O 31 | /***. RY*( 1) O 350 | 0.09 | 2.14 | 0.01 | |
| 9. BD ( 1) H 9 - O 31 | /***. RY*( 3) O 350 | 0.06 | 2.12 | 0.01 | |
| 9. BD ( 1) H 9 - O 31 | /***. RY*( 1) H 358 | 0.14 | 1.63 | 0.01 | |
| 9. BD ( 1) H 9 - O 31 | /***. BD*( 1) O 350 - C 355 | 0.25 | 1.68 | 0.02 | |
| 24. BD ( 1) C 18 - O 31 | /***. RY*( 1) H 358 | 0.06 | 1.88 | 0.01 | |
| **from unit 6 to unit 5** | | | | | |
| 384. BD ( 1) O 343 - C 355 | /***. BD*( 1) H 9 - O 31 | 0.20 | 1.49 | 0.02 | |
| 389. BD ( 1) O 350 - C 355 | /***. RY*( 1) H 9 | 0.05 | 2.58 | 0.01 | |
| 389. BD ( 1) O 350 - C 355 | /***. RY*( 4) H 9 | 0.05 | 3.44 | 0.01 | |
| 389. BD ( 1) O 350 - C 355 | /***. BD*( 1) H 9 - O 31 | 0.06 | 1.80 | 0.01 | |
| 390. BD ( 2) O 350 - C 355 | /***. BD*( 1) H 9 - O 31 | 0.12 | 0.95 | 0.01 | |
| 924. LP ( 1) O 350 | /***. BD*( 1) H 9 - O 31 | 6.40 | 1.22 | 0.08 | |
| 924. LP ( 1) O 350 | /***. BD*( 1) C 18 - O 31 | 0.06 | 1.24 | 0.01 | |
| 925. LP ( 2) O 350 | /***. RY*( 4) O 31 | 0.15 | 1.51 | 0.01 | |
| 925. LP ( 2) O 350 | /***. BD*( 1) H 9 - O 31 | 5.08 | 0.82 | 0.06 | |
| 925. LP ( 2) O 350 | /***. BD*( 1) C 18 - O 31 | 0.15 | 0.84 | 0.01 | |
| **Total interactions between hydrogen-bonded mol 1 and mol 2** | | **12.92** | **25.34** | **0.29** | |

^#^E(*i*) corresponds to Lewis type “filled” donor orbitals. E(*j*) corresponds to non-Lewis type “unfilled” acceptor orbitals. LP = lone-pair, LP* = anti-bonding lone pair, BD = 2-center bond, BD* = 2-center anti-bond, RY = Rydberg orbital, RY* = Rydberg anti-bond orbitals.

For each donor NBO (*i*) and acceptor NBO (*j*), the stabilization energy *E*(2) associated with *i* → *j* delocalization is determined as,

$$E\left( 2 \right)= E_{ij}^{(2)} =\frac{q_{i}F\left( i,j \right)^{2}}{{}_{j}-{}_{i}}$$

Where, *q*_i_: is the donor orbital occupancy.

*ε*_i_, *ε*_j_ are diagonal elements (orbital energies) of NBO Fock matrix.

*F*(*i*,*j*) is the off-diagonal NBO Fock matrix elements.

**Table S11.** The volume of the void available for solvent-accessible surface within the unit cell of BGPHEOH at different temperatures.

| Temperature | 296K_2 | 305K_2 | 315K_2 | 325K_2 | 335K_2 | 337K_3 | 339K_3 | 340K_3 |
| --- | --- | --- | --- | --- | --- | --- | --- | --- |
| Solvent accessible surface | | | | | | | | |
| Probe radius (Å) | 0.96 | 0.96 | 0.96 | 0.96 | 0.96 | 0.96 | 0.96 | 0.96 |
| Approximate Grid spacing (Å) | 0.1 | 0.1 | 0.1 | 0.1 | 0.1 | 0.1 | 0.1 | 0.1 |
| Volume % of the unit cell | 0.5 | 0.5 | 0.5 | 0.6 | 0.6 | 0.6 | 0.5 | 0.5 |
| Volume of the void (Å^3^) | 9.03 | 7.80 | 8.04 | 9.79 | 10.65 | 10.45 | 7.80 | 7.98 |
